# Exploring the Feasibility of Multi-Site Flow Cytometric Processing of Gut Associated Lymphoid Tissue with Centralized Data Analysis for Multi-Site Clinical Trials

**DOI:** 10.1371/journal.pone.0126454

**Published:** 2015-05-26

**Authors:** Ian McGowan, Peter A. Anton, Julie Elliott, Ross D. Cranston, Kathryn Duffill, Andrew D. Althouse, Kevin L. Hawkins, Stephen C. De Rosa

**Affiliations:** 1 University of Pittsburgh School of Medicine, Pittsburgh, Pennsylvania, United States of America; 2 Department of Medicine and the AIDS Institute, David Geffen School of Medicine at University of California Los Angeles, Los Angeles, California, United States of America; 3 Magee-Womens Research Institute, Pittsburgh, Pennsylvania, United States of America; 4 Vaccine and Infectious Disease Division, Fred Hutchinson Cancer Research Center, Seattle, Washington, United States of America; 5 Department of Laboratory Medicine, University of Washington, Seattle, Washington, United States of America; University of Cape Town, SOUTH AFRICA

## Abstract

The purpose of this study was to determine whether the development of a standardized approach to the collection of intestinal tissue from healthy volunteers, isolation of gut associated lymphoid tissue mucosal mononuclear cells (MMC), and characterization of mucosal T cell phenotypes by flow cytometry was sufficient to minimize differences in the normative ranges of flow parameters generated at two trial sites. Forty healthy male study participants were enrolled in Pittsburgh and Los Angeles. MMC were isolated from rectal biopsies using the same biopsy acquisition and enzymatic digestion protocols. As an additional comparator, peripheral blood mononuclear cells (PBMC) were collected from the study participants. For quality control, cryopreserved PBMC from a single donor were supplied to both sites from a central repository (qPBMC). Using a jointly optimized standard operating procedure, cells were isolated from tissue and blood and stained with monoclonal antibodies targeted to T cell phenotypic markers. Site-specific flow data were analyzed by an independent center which analyzed all data from both sites. Ranges for frequencies for overall CD4+ and CD8+ T cells, derived from the qPBMC samples, were equivalent at both UCLA and MWRI. However, there were significant differences across sites for the majority of T cell activation and memory subsets in qPBMC as well as PBMC and MMC. Standardized protocols to collect, stain, and analyze MMC and PBMC, including centralized analysis, can reduce but not exclude variability in reporting flow data within multi-site studies. Based on these data, centralized processing, flow cytometry, and analysis of samples may provide more robust data across multi-site studies. Centralized processing requires either shipping of fresh samples or cryopreservation and the decision to perform centralized versus site processing needs to take into account the drawbacks and restrictions associated with each method.

## Introduction

There is increasing interest in characterizing and quantifying T cell populations in lymphoid tissue as a component of translational studies focused on HIV pathogenesis and/or the evaluation of novel strategies to treat or prevent HIV infection [1–4]. The cervicovaginal and rectal mucosae are the primary routes of sexually acquired HIV infection. HIV vaccines and antiretroviral microbicide products are being developed to prevent mucosal HIV infection [5;6]. A fundamental research question within HIV prevention science is whether the use of vaccines or microbicides could induce local immune responses that might modulate the risk of HIV acquisition. In order to address this question, many Phase 1 vaccine and microbicide trials incorporate collection of mucosal samples with isolation of mucosal mononuclear cells (MMCs) whose phenotypic changes can then be characterized using flow cytometry [3;4].

In multi-site studies involving flow cytometric evaluation of MMCs, investigators can ship mucosal samples to a central processing and analysis facility or process and evaluate samples locally with subsequent compilation of site-acquired/analyzed data. However, it is unclear whether MMC flow data generated at one site can subsequently be compared with data generated at a second or third site. Differences in study populations, tissue acquisition, and T cell isolation may prevent direct comparison between data sets. The problem is further exacerbated by use of different flow cytometer platforms and/or gating strategies.

In contrast, there is a well-established process to monitor inter- and intra-laboratory performance of peripheral blood mononuclear cell (PBMC) flow cytometry in multi-site trials through the use of standardized flow cytometry staining panels and use of cryopreserved aliquots of the same PBMC sample for quality control [7]. Repeated evaluation of laboratories conducting PBMC flow cytometry has also been shown to improve the overall proficiency of the laboratories [8]. Unfortunately, a similar capability does not exist for laboratories conducting MMC flow cytometry.

The purpose of this pilot study was to evaluate a standardized approach to the collection of intestinal tissue, isolation of gut associated lymphoid tissue (GALT) MMCs, and characterization of T cell phenotypes (activation and memory) at two participating sites: the McGowan laboratory at the Magee-Womens Research Institute (MWRI) at the University of Pittsburgh School of Medicine and the Anton laboratory at the David Geffen School of Medicine at UCLA.

## Materials and Methods

The protocol for this study is available as supporting information (S1 File).

### Ethics statement

This study was approved by the University of Pittsburgh School of Medicine Institutional Review Board (IRB# PRO10090390) and UCLA Office of Human Research Protection Institutional Review Board (IRB# 11–000666) with all participants providing written informed consent.

### Objectives

The primary objective of this study was to determine whether development of a standardized approach to the collection of intestinal tissue from healthy volunteers, isolation of GALT MMC, and flow cytometric characterization of T cell populations was sufficient to minimize differences in the normative ranges generated by multiple sites. A second goal was to assess the need for fluorescence-minus-one (FMO) controls to be performed on all tissue types on all samples [9].

### Study subjects

To minimize study population heterogeneity only male participants were enrolled into the study. Exclusion criteria included positive HIV-1 serology or evidence of rectal infection with *Chlamydia trachomatis* or *Neisseria gonorrhea* as well as any other gastrointestinal disorders or chronic systemic conditions (details in Protocol). At the start of the study we excluded participants with positive serology for either herpes simplex virus type 1 (HSV-1) or HSV-2. UCLA found it difficult to enroll HSV-1/2 negative participants and so in order to expedite study recruitment the HSV serology exclusion criteria was dropped. Seven of the UCLA participants were HSV-1 seropositive and none of the participants were HSV-2 seropositive.

### Preparation of qPBMC for staining

The HIV Vaccine Trials Network (HVTN) laboratory (University of Washington, Seattle, WA) provided each study site with 45 frozen PBMC aliquots each containing 25 x 10^6^ PBMC obtained from a single donor. These were obtained from a single leukapheresis. PBMC were isolated by density centrifugation with Histopaque (Sigma-Aldrich, St. Louis, MO) and were cryopreserved in aliquots. These cells are referred to as qPBMC to differentiate them from the study participants’ PBMC and were included as a quality control to be used with each participant’s MMC and PBMC sample. As required, each vial of frozen qPBMCs was placed in a 37°C water bath after removal from the -80°C freezer. The vial of cells was then transferred to 50 ml conical tubes containing 8 ml of cRPMI medium. The cells were centrifuged at 1600 rpm (515 x g) for 10 minutes. After decanting the supernatant fluid, the volume of remaining medium and cells was adjusted to 1 ml. Cell counts were obtained using a hemocytometer.

### PBMC collection and isolation of lymphocyte populations

Ten ml of heparinized whole blood was collected from each participant at the time of endoscopy. Whole blood was diluted with an equal volume of Dulbecco's Phosphate-Buffered Saline (D-PBS; Life Technologies, Grand Island, NY) and placed in a 50 ml conical tube. Lymphocytes were isolated using differential centrifugation with Histopaque 1077 (Sigma-Aldrich, St. Louis, MO). The mononuclear cell layer (interface) was removed carefully with a Pasteur pipette and placed in a clean, sterile 50 ml conical tube and washed with 50 ml of D-PBS. The cells were pelleted by centrifugation for 5 min at 2000 rpm (800 x g) and washed twice with 50 ml D-PBS. The cell pellet was resuspended in approximately 5 ml D-PBS. Cell counts were obtained using a hemocytometer.

### Mucosal sampling and isolation of mucosal mononuclear cells

Flexible sigmoidoscopy was performed with collection of 15 rectal biopsies acquired at approximately 15 cm from the anal verge. Biopsies (8 mm x 2 mm x 1 mm from large-cup, endoscopic biopsy forceps; Microvasive Radial Jaw #1589, outside diameter 3.3 mm, Boston Scientific, Marlborough, MA) were collected and immediately placed into 15 ml of tissue culture medium (RPMI 1640, Irvine Scientific, Santa Ana, CA). Mucosal mononuclear cells were isolated from rectal biopsies using a combination of mechanical and enzyme digestion as previously described [10]. Generally, only one subject was biopsied each day at either site. Thus, each experiment consisted of data for the mucosal biopsy and the PBMC from a single participant along with the data for the qPBMC thawed for that experiment.

### Preparation of qPBMC, PBMC, and MMC for flow cytometry

Immunophenotyping by flow cytometry was performed by pre-staining with a viability dye followed by staining with one of two different eight-color study panels (Table 1). Panel 1 was stained for cell-surface antigens that characterize a memory phenotype (CD45, CD3, CD4, CD8, CD45RA, CCR5, and CD27). Panel 2 was stained for surface antigens indicative of an activation phenotype (CD45, CD3, CD4, CD8, HLA-DR, CD38, and CD69). Fluorescent conjugates of antibody reagents to these antigens were obtained from Becton Dickinson (BD), San Jose, CA, or eBiosciences, San Diego, CA (Table 1). FMO controls were included to define the negative gates for selected populations in Panel 1 (CD45RA, CCR5, and CD27) and Panel 2 (HLA-DR, CD38, and CD69). Cell viability was assessed using the LIVE/DEAD Fixable Dead Cell Stain Kit (Invitrogen, Grand Island, NY). Antibody volumes were determined by performing titration on PBMC at one site laboratory prior to beginning the study. Reagents where then purchased in bulk so that the same lots were used for cell isolation at both sites throughout the study.

**Table 1:** Monoclonal antibody reagents used in the study.

Marker	Catalogue number	Clone
**T Cell Memory Panel**		
CD45 PerCP	BD# 340665	2D1
CD3 Pac Blue	BD# 558117	UCHT1
CD4 PE-Cy7	BD # 557852	SK3
CD8 APC-H7	BD# 557834	SK1
CD45RA FITC	BD# 347723	HI100
CCR5 PE	BD# 555993	2D7/CCR5
CD27 APC	eBioscience#17–0279	0323
**T Cell Activation Panel**		
CD45 PerCP	BD# 340665	2D1
CD3 Pac Blue	BD# 558117	UCHT1
CD4 PE-Cy7	BD # 557852	SK3
CD8 APC-H7	BD# 557834	SK1
HLA-DR FITC	BD # 347363	L243
CD38 PE	BD # 342371	HB7
CD69 APC	BD # 340560	L78

### Flow cytometer compensation

Compensation was set individually for each of the fluorochromes in the panels. One set of singly-stained compensation samples was prepared for each flow cytometry run, using BD positive and BD negative compensation beads (Anti-Mouse Ig, κ/Negative Control (FBS) Compensation Particles Set, #552843, BD Biosciences, San Jose, CA). Compensation staining for the Aqua fluorescent dye was performed separately using the ArC Amine Reactive Compensation Bead Kit (Invitrogen Catalog #A10346, Invitrogen Corporation, Carlsbad, CA).

### Standardization of flow cytometers at each site

Flow cytometric analysis was performed independently at both sites. The BD LSRFortessa cytometer (BD Biosciences, San Jose, CA) was used at the MWRI site and the BD LSRII cytometer (BD Biosciences, San Jose, CA) was used at the UCLA site. Each site prepared a baseline report using the same lot number of BD Cytometer Set Up & Tracking beads (CS&T) (BD Biosciences, San Jose, CA). The baseline report provided information of the cytometer’s performance by measuring key factors, such as determining a target median fluorescent intensity (MFI) and a linear range for each parameter. Application settings were calculated using both instruments to provide consistent photomultiplier tube (PMT) target MFI values for reproducible cytometer settings across the two flow cytometers at the two sites over the course of the study. This was achieved using unstained qPBMCs supplied for this study and adjusting the voltages for each detector to set the robust standard deviation of the electronic noise (rSDEN) of the instrument 2.5–3.0 above the value provided on the baseline report. Once the voltages were determined, a stained sample labeled with all the reagents used in the study was run to ensure the brightest populations for each parameter were within the linear max as defined on the cytometer baseline report. These settings were saved as application settings for the entire study. Using the newly created application settings, that lot of CS&T beads was collected on the cytometer and recorded. This procedure was performed on the cytometer at MWRI. These values were exported to a flash drive and were provided to the UCLA site where they were imported onto the LSRII. UCLA, using the same lot of the CS&T beads, adjusted the voltages so that the MFI in each channel matched the established targets MFI’s established at MWRI. These were, recorded and saved as application settings on that instrument. Each time the samples were run, application settings at both sites were used to yield consistent results.

### Running the cells and compensation beads

Approximately 5,000 compensation beads per tube were acquired. Approximately 20,000 CD8+ positive events from the cell samples were recorded per immunophenotyping tube.

### Analysis of flow cytometric data

**Fig 1:**
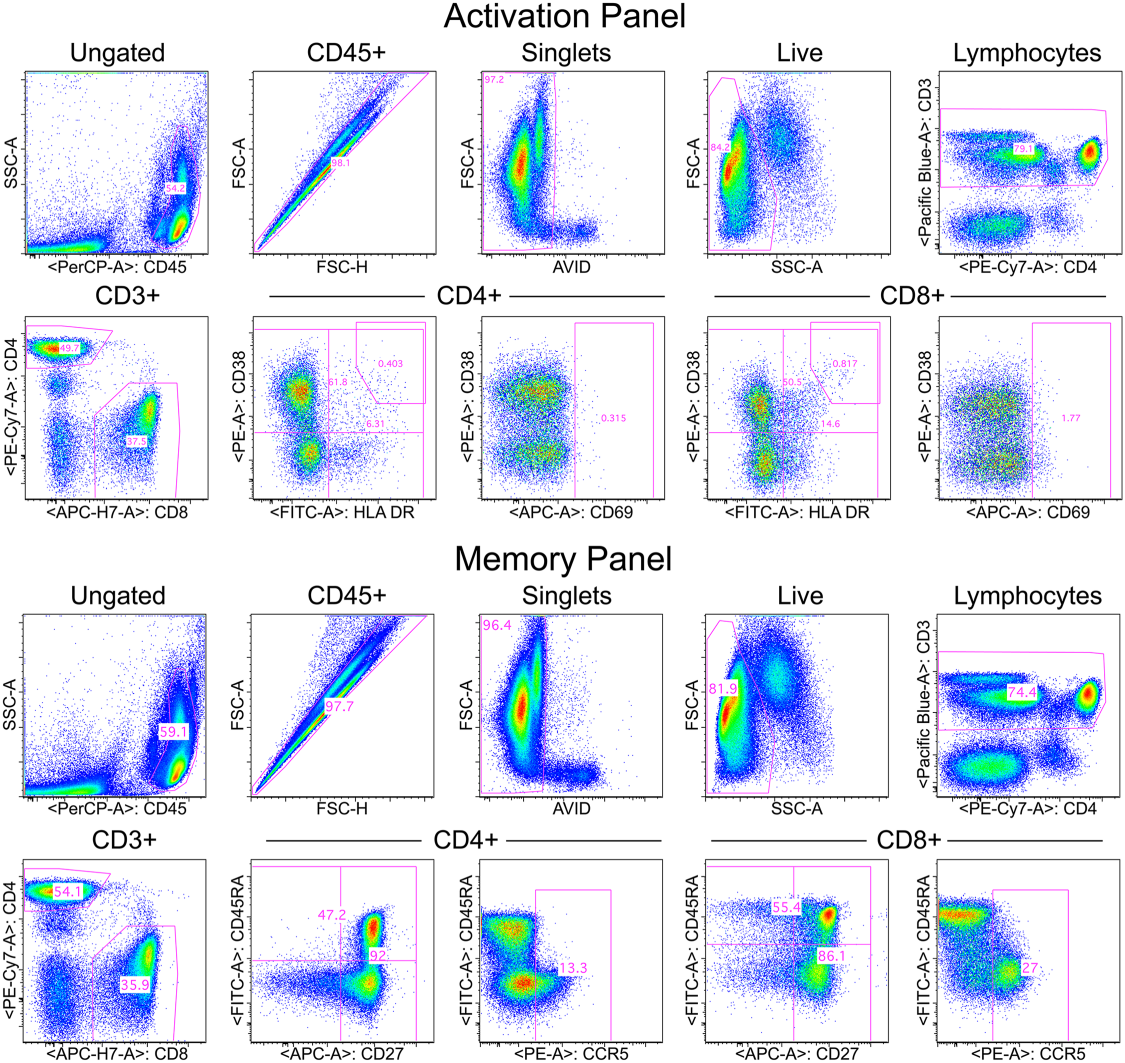
Gating strategy for activation panel (upper) and memory panel (lower). The sequential gating to CD3+ T cells is the same for both panels as shown in the upper row for each panel. Gating on CD4+ and CD8+ T cells is shown in the lower left graph in each panel. Markers of interest for each panel for CD4+ and CD8+ T cells are shown in the remaining plots.

Flow cytometry standard (FCS) files generated by both sites were analyzed at each site and also transferred to the HVTN laboratory where they were analyzed by a single investigator using FlowJo software version 9 (Tree Star, Inc., Ashland, OR). The hierarchical gating strategy used to identify cell populations of interest is illustrated in Fig 1.

### Statistics

All flow cytometric data are percentages. CD4+ and CD8+ refer to the percent of CD3+ T cells expressing CD4 or CD8. The memory and activation markers refer to the percent of CD4+ or CD8+ T cells expressing (or not expressing) the indicated markers. All statistical testing for the manuscript was performed by a statistician at the MWRI site (AA). For each of the cell populations of interest (6 from the activation panel and 12 from the memory panel), we present the mean and standard deviation (SD) for the entire study population and for each of the laboratories (MWRI and UCLA) as well as boxplots showing the median, quartiles, and range for each cell population. To test for between-laboratory differences in measurement, we compared MWRI measurements to UCLA measurements using both the two-sample t-test and the non-parametric Wilcoxon rank-sum test. The t-test is the most common test used to compare the distribution of a continuous variable in two independent samples, but given the presence of outliers and the relatively small sample size, we also present Wilcoxon rank-sum tests, which are more robust and less influenced by outliers.

A significant result for one marker on both the t-test and the Wilcoxon rank-sum test is strong evidence of a difference between labs in that particular marker. The spread of the respective distributions was also compared using a Folded F-test to compare the variances for the MWRI lab versus the UCLA lab; a significant result indicates that one distribution had significantly greater variability than the other (regardless of the center of the respective distributions). The data are displayed graphically using box-and-whisker plots; the p-values reported on these figures correspond to the Wilcoxon rank-sum test for each marker.

The primary between-laboratory comparisons were conducted using the qPBMC data. All of the same methods described above were also used to analyze the PBMC data and MMC data but these should be interpreted with caution as they are taken on different participant samples at the respective sites and thus, significant differences may not be indicative of between-laboratory differences in measurement, but differences in the participant populations recruited at the two sites. In addition, Levey-Jennings plots were prepared separately for qPBMC data from each site and visually show the mean and +/- 3 SD from the mean. Values beyond 3 SD were considered outliers and reasons for potential assay irregularities were investigated for those flow data sets with outliers. All statistical analyses were performed using SAS version 9.4 (SAS Institute, Cary, NC). P-values less than 0.05 were considered statistically significant; no adjustments were made for multiple comparisons.

## Results

### Participant demographics

A total of 77 participants were screened (34 at MWRI and 43 at UCLA) for the study to enable enrollment of 20 participants at both sites (Table 2). The mean age for the participants was 28.9 (± 9.3) and 35.4 (± 12.5) years at MWRI and UCLA, respectively. The HSV status of participants by study site is presented in Table 2. One participant at MWRI had incomplete flow data and was excluded from the comparative data analysis.

**Table 2:** Study Populations.

	UCLA	MWRI
**Mean Age (± SD)**	35.4 (12.5)	28.9 (9.3)
**HSV-1 positivity**	7/20 (35%)	0/20 (0%)
**HSV-2 positivity**	0/20 (0%)	0/20 (0%)

### Standardized flow cytometric gating of cell populations

Because of the assay standardization and the standardization of the instrument settings, it was possible to apply a standard gating template to nearly all the data and this template was almost identical for the data from the two sites (Fig 1 shows the gating hierarchy). One objective of this study was to assess the need to perform FMO controls within each experiment and on each sample type in order to properly place gates for those cell markers that did not have distinct separation between positive and negative cells. The markers of concern were CD69, CD38 and HLA-DR in the activation panel and CCR5, CD27 and CD45RA in the memory panel. FMOs were initially performed for these markers for the qPBMC, PBMC and MMC samples.

**Fig 2:**
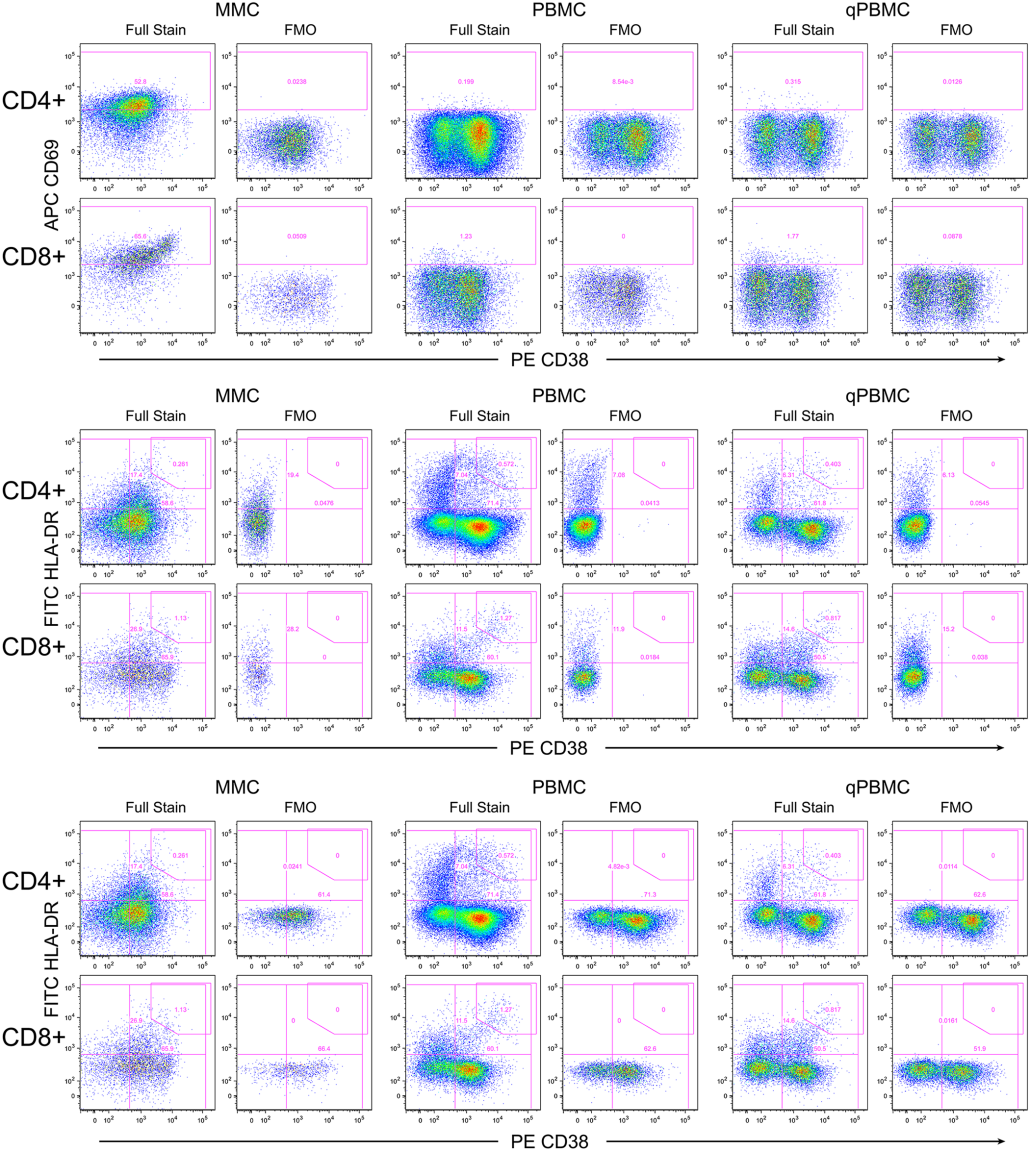
Fluorescence-minus-one (FMO) controls for the activation panel. Shown is an example from one experiment at one site. The upper graphs show the FMO for APC CD69, the middle for PE CD38, and the lower for FITC HLA-DR. The three specimen types are shown with MMC on the left, PBMC in the middle and qPBMC on the right. For each, graphs are paired with the full stain on the left and the FMO on the right. Note that the FMO defines the lower limit of the gate; often the gate is placed higher.

Interim analysis of the flow data plots showed that the placement of gates based on FMOs were the same for all three specimen types (examples: Fig 2 for activation panel, S1 Fig for memory panel). Based on these findings, it was determined that performing the FMOs on one specimen type would determine the gates for all specimen types. FMOs were only performed on the qPBMC for participants enrolled later in the study. This finding also helped conserve the more limited MMC specimen for the fully-stained panels. At the end of the study, comparison of FMOs between different flow panel data sets revealed that the optimal FMO-defined gates did not change substantially between samples. This indicated that it was not necessary to perform any FMOs in each experiment. Rather, a single FMO performed at the beginning of the study on one sample type such as qPBMC was sufficient to determine optimal gate placement.

Since gates were slightly different between sites, it is recommended to perform one FMO for each site. It should be noted that the extensive standardization in this study enabled template gating to analyze flow data from the study samples. Without such standardization, it may be necessary to perform selected FMOs in each experiment. But even in that case, FMOs on only one specimen type would be needed. It is important to note that FMOs determine the lower limit for a gate; higher gates can be used if appropriate as for the CD38+HLA-DR+ gate since these cells are typically gated for bright co-expression of these two markers.

### Quality assurance and trending of control PBMC over time

**Fig 3:**
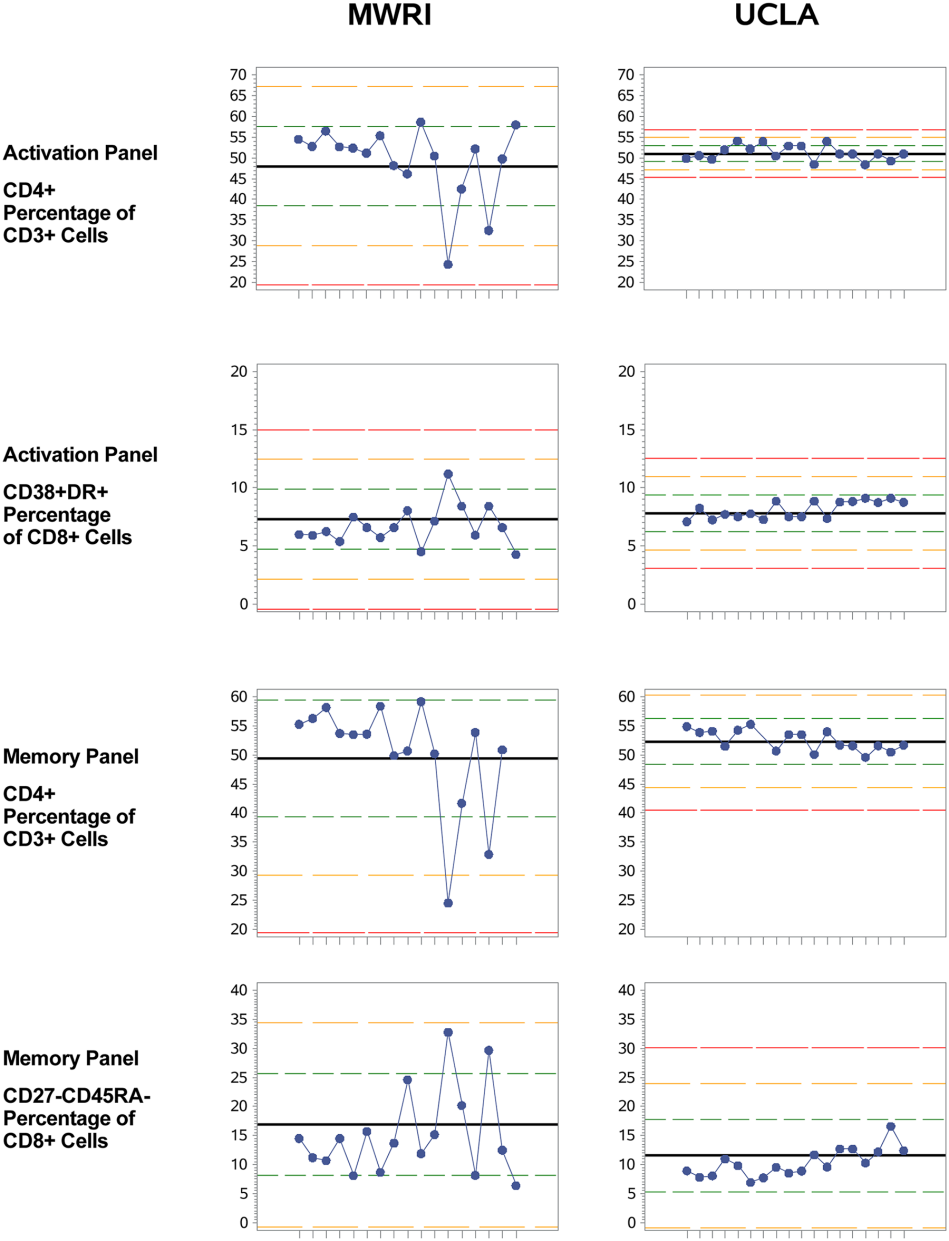
qPBMC Levey-Jennings plots for the CD4+ percentage of CD3+ cells and the CD8+ CD38+DR+ percentage of CD8+ cell populations from the activation panel and the CD4+ percentage of CD3+ cells and the CD27-CD45RA- percentage of CD8+ cell populations from the memory panel. Each experiment (subject) is shown on the x-axis. The bold black line shows the mean, and the dotted red line shows +/- 3 SD from the mean. Green and yellow lines are for 1 and 2 SD.

Because qPBMC were thawed and analyzed in each experiment along with the participant PBMC and mucosal samples, results for the qPBMC sample could be trended over time to assess consistency in the measurement of the various cell populations identified with the two flow cytometric staining panels. Levey-Jennings plots of qPBMC data were created for all data from each site over time (Fig 3 shows representative examples for the CD4+ and CD8+ CD38+DR+ cell populations from the activation panel and the CD4+ and CD8+ CD27-CD45RA- cell populations from the memory panel; Levey-Jennings plots for all 18 cell populations can be found in S2 Fig). Examination of these plots revealed a few flow data sets with outliers, i.e., data beyond three standard deviations from the site mean. Based on this, two samples from MWRI (subject MWRI-002, due to high percentage HLA-DR+ cells, and MWRI-012, due to low CD27+, on the Levey-Jennings plots) and two samples from UCLA (subjects UCLA-042, high CD69+, and UCLA-060, low CD38+, low CD27+, on the Levey-Jennings plots) were excluded from further analysis due to suspicions of poor assay quality. In all cases of outliers identified through the Levey-Jennings plots, the raw flow data were examined to ensure that the reason for the unusually low or high value was not simply due to the position of the gate. Gate placement was not the cause for these outliers.

### Control PBMC comparison between sites

In addition to trending data within one site over time, the qPBMC data can be used to compare data between different sites. The primary between-laboratory results are summarized in Table 3. There were no significant between-laboratory differences in the mean or the median for percentages for CD4+ and CD8+ T cells as determined with the activation panel; the same is true for the memory panel. However, it should be noted that the variances for CD4+ and CD8+ T cell percentages were significantly higher in the MWRI measurements than in the UCLA measurements (but always with a consistent, similar intra-laboratory profile for both labs, p<0.01 for both CD4+ and CD8+ T cells on both the activation panel and the memory panel), indicating greater spread in the MWRI laboratory’s measurements than the UCLA laboratory’s measurements for these markers. Notably, the viability of the qPBMC samples was significantly greater at UCLA than at MWRI for both the activation panel and the memory panel; this could account for the differences in means and the lower variances between laboratories although the extent of the difference was minor since viabilities for both sites were excellent (means >93%).

**Table 3:** qPBMC Comparisons.

Characteristic	Total	MWRI	UCLA	t-test	Wilcoxon	Folded F
	(N = 35)	(N = 17)	(N = 18)	p-value	p-value	test
	(Mean ± SD)					(Variance)
**Activation Panel**						
Viability of CD3+ cells	94.7 ± 3.2	93.5 ± 3.9	95.9 ± 1.7	0.012	0.018	<.001
CD4+ % of CD3+ cells	50.3 ± 6.4	49.3 ± 9.0	51.3 ± 1.8	0.389	0.779	<.001
CD38+DR+ % of CD4+ cells	1.7 ± 0.4	1.4 ± 0.2	2.0 ± 0.3	<.001	<.001	0.24
CD69+ % of CD4+ cells	0.7 ± 0.4	0.5 ± 0.1	0.9 ± 0.6	0.009	0.089	<.001
CD8+ % of CD3+ cells	38.8 ± 3.9	39.7 ± 5.2	38.0 ± 1.7	0.202	0.498	<.001
CD38+DR+ % of CD8+ cells	7.5 ± 1.4	6.7 ± 1.7	8.1 ± 0.7	0.004	0.001	0.002
CD69+ % of CD8+ cells	3.0 ± 0.8	3.0 ± 0.5	3.1 ± 1.1	0.825	0.855	0.004
**Memory Panel**						
Viability of CD3+ cells	94.9 ± 3.0	93.7 ± 3.7	96.0 ± 1.6	0.013	0.020	<.001
CD4+ % of CD3+ cells	51.9 ± 7.0	50.8 ± 9.5	53.1 ± 2.9	0.352	0.947	<.001
CCR5+ % of CD4+ cells	10.6 ± 4.1	8.7 ± 2.8	12.5 ± 4.4	0.004	0.004	0.078
CD27+CD45RA+% of CD4+ cells	43.0 ± 5.3	41.1 ± 4.6	44.7 ± 5.5	0.043	0.024	0.475
CD27+CD45RA- % of CD4+ cells	42.0 ± 6.4	40.1 ± 6.6	43.8 ± 5.7	0.089	0.477	0.578
CD27-CD45RA+ % of CD4+ cells	0.4 ± 0.4	0.4 ± 0.4	0.4 ± 0.4	0.699	0.753	0.976
CD27-CD45RA- % of CD4+ cells	14.6 ± 7.4	18.3 ± 8.9	11.1 ± 2.8	0.005	0.010	<.001
CD8+ % of CD3+ cells	37.6 ± 5.1	38.8 ± 6.5	36.5 ± 3.0	0.191	0.428	0.003
CCR5+ % of CD8+ cells	29.3 ± 4.2	28.3 ± 3.9	30.3 ± 4.4	0.160	0.209	0.656
CD27+CD45RA+ % of CD8+ cells	42.1 ± 7.2	38.7 ± 8.5	45.3 ± 3.5	0.007	0.004	<.001
CD27+CD45RA- % of CD8+ cells	34.6 ± 5.0	33.6 ± 5.1	35.5 ± 4.8	0.257	0.632	0.759
CD27-CD45RA % of CD8+ cells	10.6 ± 4.2	12.4 ± 5.1	8.8 ± 1.8	0.012	0.006	<.001
CD27-CD45RA- % of CD8+ cells	12.7 ± 6.0	15.2 ± 7.5	10.3 ± 2.4	0.019	0.026	<.001

**Fig 4:**
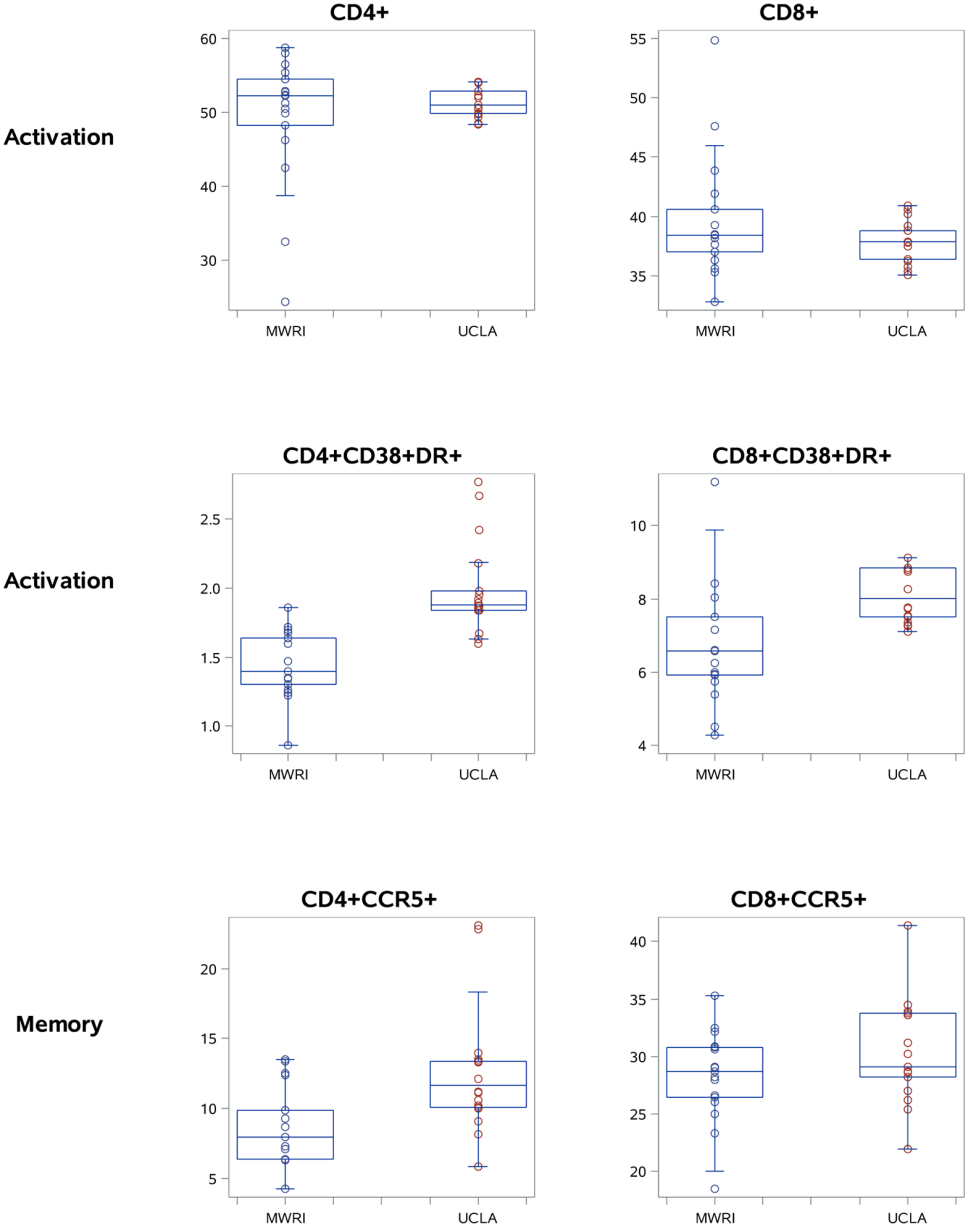
Selected qPBMC data for CD4+, CD8+, CD4+CCR5+, CD8+CCR5+, CD4+CD38+DR+, and CD8+CD38DR+ T cells (boxplots showing the median, quartiles, and range for each cell population).

Moving beyond the CD4+ and CD8+ T cell percentages, there were significant between-laboratory differences in the means in qPBMCs for two cell populations from the activation panel (CD4+CD38+DR+ and CD8+CD38+DR+) and seven cell populations from the memory panel (CD4+CCR5+, CD4+CD27+CD45RA+, CD4+CD27-CD45RA-, CD8+CD27+CD45RA+, CD8+CD27-CD45RA+, and CD8+CD27-CD45RA-). There were also significant between-laboratory differences in variance for five populations from the activation panel and seven populations from the memory panel, all indicating stable but greater variance in the MWRI measurements than the UCLA measurements. Fig 4 provides a graphical representation of the mean and spread at each laboratory for 6 selected populations in the qPBMC data (CD4+, CD8+, CD4+CD38+DR+, CD8+CD38+DR+, CD4+CCR5+, and CD8+CCR5+).

### Participant PBMC and MMC: between site comparison results

Using data from subjects not excluded by the above-mentioned criteria, 17 paired PBMC-MMC data sets from MWRI and 18 data sets from UCLA were compared in the centralized analysis. In the participant PBMC data (Table 4), there were significant between-laboratory differences in the mean for one cell population from the activation panel and three populations from the memory panel, plus significant differences in variance between sites for three populations from the activation panel and two populations from the memory panel.

**Table 4:** PBMC Comparisons.

Characteristic	Total	MWRI	UCLA	t-test	Wilcoxon	Folded F
	(N = 35)	(N = 17)	(N = 18)	p-value	p-value	test
	(Mean ± SD)					(Variance)
**Activation Panel**						
Viability of CD3+ cells	99.0 ± 1.3	98.9 ± 1.4	99.0 ± 1.3	0.837	0.204	0.398
CD4+ % of CD3+ cells	65.2 ± 10.6	67.8 ± 8.8	62.8 ± 11.7	0.161	0.192	0.269
CD38+DR+ % of CD4+ cells	2.0 ± 2.1	1.1 ± 0.6	2.8 ± 2.7	0.020	<.001	<.001
CD69+ % of CD4+ cells	3.1 ± 4.9	2.0 ± 2.9	4.2 ± 6.0	0.180	0.018	0.006
CD8+ % of CD3+ cells	28.7 ± 8.8	26.6 ± 7.8	30.6 ± 9.5	0.175	0.160	0.440
CD38+DR+ % of CD8+ cells	6.1 ± 4.8	4.6 ± 2.9	7.6 ± 5.7	0.056	0.067	0.011
CD69+ % of CD8+ cells	7.1 ± 7.6	6.4 ± 5.9	7.7 ± 9.0	0.625	0.679	0.096
**Memory Panel**						
Viability of CD3+ cells	98.7 ± 1.7	98.9 ± 1.3	98.6 ± 2.1	0.512	0.310	0.031
CD4+ % of CD3+ cells	66.4 ± 10.2	68.8 ± 8.4	64.2 ± 11.5	0.187	0.203	0.213
CCR5+ % of CD4+ cells	9.8 ± 8.4	6.7 ± 5.8	12.6 ± 9.5	0.040	0.021	0.064
CD27+CD45RA+% of CD4+ cells	44.9 ± 16.4	41.0 ± 15.0	48.8 ± 17.2	0.167	0.255	0.582
CD27+CD45RA- % of CD4+ cells	43.9 ± 13.4	48.8 ± 12.5	39.0 ± 12.8	0.030	0.058	0.943
CD27-CD45RA+ % of CD4+ cells	0.9 ± 1.5	0.4 ± 0.5	1.4 ± 1.9	0.052	0.535	<.001
CD27-CD45RA- % of CD4+ cells	10.3 ± 5.2	9.8 ± 4.0	10.8 ± 6.3	0.593	0.958	0.083
CD8+ % of CD3+ cells	27.0 ± 8.8	25.1 ± 7.4	28.8 ± 9.8	0.225	0.306	0.254
CCR5+ % of CD8+ cells	24.9 ± 12.6	24.0 ± 10.3	25.7 ± 14.6	0.709	1.000	0.181
CD27+CD45RA+ % of CD8+ cells	45.3 ± 18.0	42.0 ± 17.9	48.5 ± 17.9	0.297	0.408	0.995
CD27+CD45RA- % of CD8+ cells	25.7 ± 9.9	30.2 ± 9.3	21.2 ± 8.5	0.006	0.008	0.724
CD27-CD45RA % of CD8+ cells	15.9 ± 12.1	14.7 ± 11.8	17.0 ± 12.7	0.596	0.679	0.762
CD27-CD45RA- % of CD8+ cells	13.1 ± 7.8	13.0 ± 8.1	13.2 ± 7.7	0.956	0.849	0.836

The MMC data (Table 5) had significant between-laboratory differences in the mean for five populations from the activation panel and three populations from the memory panel, plus significant differences in variance between sites for three populations from the memory panel.

**Table 5:** MMC Comparisons.

Characteristic	Total	MWRI	UCLA	t-test	Wilcoxon	Folded F
	(N = 35)	(N = 17)	(N = 18)	p-value	p-value	test
	(Mean ± SD)					(Variance)
**Activation Panel**						
Viability of CD3+ cells	80.8 ± 15.6	77.0 ± 17.3	84.3 ± 13.4	0.143	0.040	0.276
CD4+ % of CD3+ cells	62.2 ± 11.9	66.5 ± 11.5	58.1 ± 11.0	0.033	0.010	0.851
CD38+DR+ % of CD4+ cells	9.2 ± 5.9	8.3 ± 4.5	10.1 ± 7.0	0.382	0.728	0.077
CD69+ % of CD4+ cells	60.3 ± 15.7	68.3 ± 11.6	52.7 ± 15.5	0.002	0.002	0.248
CD8+ % of CD3+ cells	30.4 ± 8.5	25.5 ± 6.6	35.0 ± 7.6	<.001	0.001	0.563
CD38+DR+ % of CD8+ cells	20.5 ± 10.0	16.4 ± 8.4	24.4 ± 10.0	0.015	0.023	0.466
CD69+ % of CD8+ cells	70.5 ± 14.9	75.9 ± 11.9	65.5 ± 16.0	0.037	0.028	0.246
**Memory Panel**						
Viability of CD3+ cells	79.6 ± 13.4	74.7 ± 20.2	84.3 ± 13.0	0.083	0.144	0.064
CD4+ % of CD3+ cells	61.8 ± 11.6	67.4 ± 9.3	56.5 ± 11.3	0.004	0.002	0.442
CCR5+ % of CD4+ cells	68.9 ± 14.7	65.6 ± 13.8	71.7 ± 15.2	0.233	0.173	0.703
CD27+CD45RA+% of CD4+ cells	3.5 ± 6.1	1.6 ± 1.3	5.5 ± 8.3	0.079	0.814	<.001
CD27+CD45RA- % of CD4+ cells	20.3 ± 10.6	22.7 ± 12.0	17.8 ± 8.5	0.187	0.304	0.199
CD27-CD45RA+ % of CD4+ cells	1.2 ± 1.6	0.6 ± 0.6	1.8 ± 2.1	0.038	0.026	<.001
CD27-CD45RA- % of CD4+ cells	75.0 ± 13.7	75.1 ± 11.9	74.9 ± 15.8	0.957	0.614	0.279
CD8+ % of CD3+ cells	31.3 ± 8.7	26.9 ± 6.9	35.5 ± 8.3	0.002	0.004	0.473
CCR5+ % of CD8+ cells	81.3 ± 13.0	81.3 ± 13.9	81.3 ± 12.5	0.992	1.000	0.678
CD27+CD45RA+ % of CD8+ cells	3.7 ± 4.1	3.5 ± 3.4	4.0 ± 4.8	0.741	0.732	0.183
CD27+CD45RA- % of CD8+ cells	17.7 ± 11.1	19.6 ± 12.7	15.7 ± 9.1	0.325	0.417	0.207
CD27-CD45RA % of CD8+ cells	3.4 ± 6.5	2.2 ± 1.7	4.6 ± 9.1	0.314	0.652	<.001
CD27-CD45RA- % of CD8+ cells	75.2 ± 15.6	74.7 ± 15.6	75.7 ± 16.1	0.861	0.787	0.903

**Fig 5:**
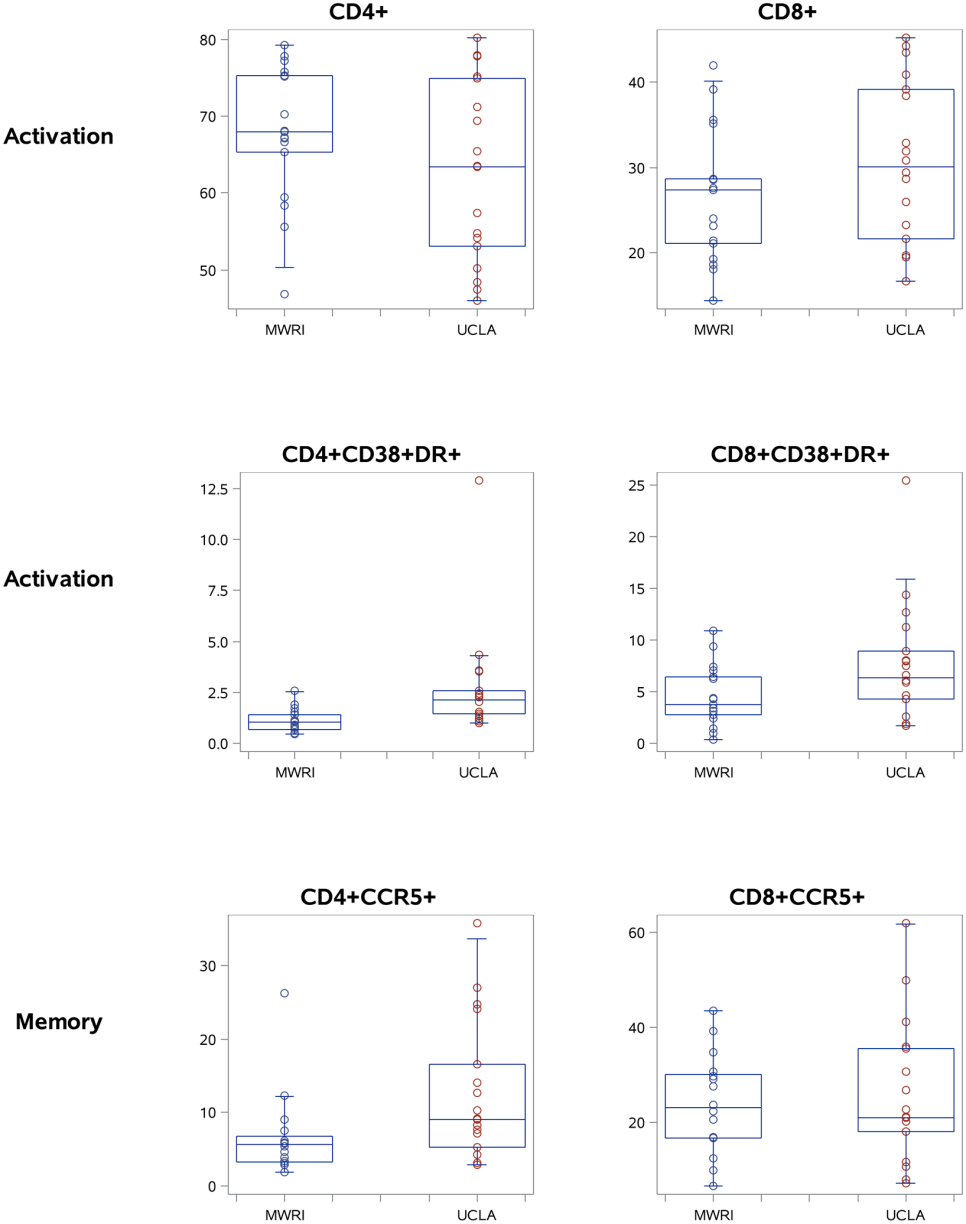
Selected PBMC data for CD4+, CD8+, CD4+CCR5+, CD8+CCR5+, CD4+CD38+DR+, and CD8+CD38DR+ T cells (boxplots showing the median, quartiles, and range for each cell population).

**Fig 6:**
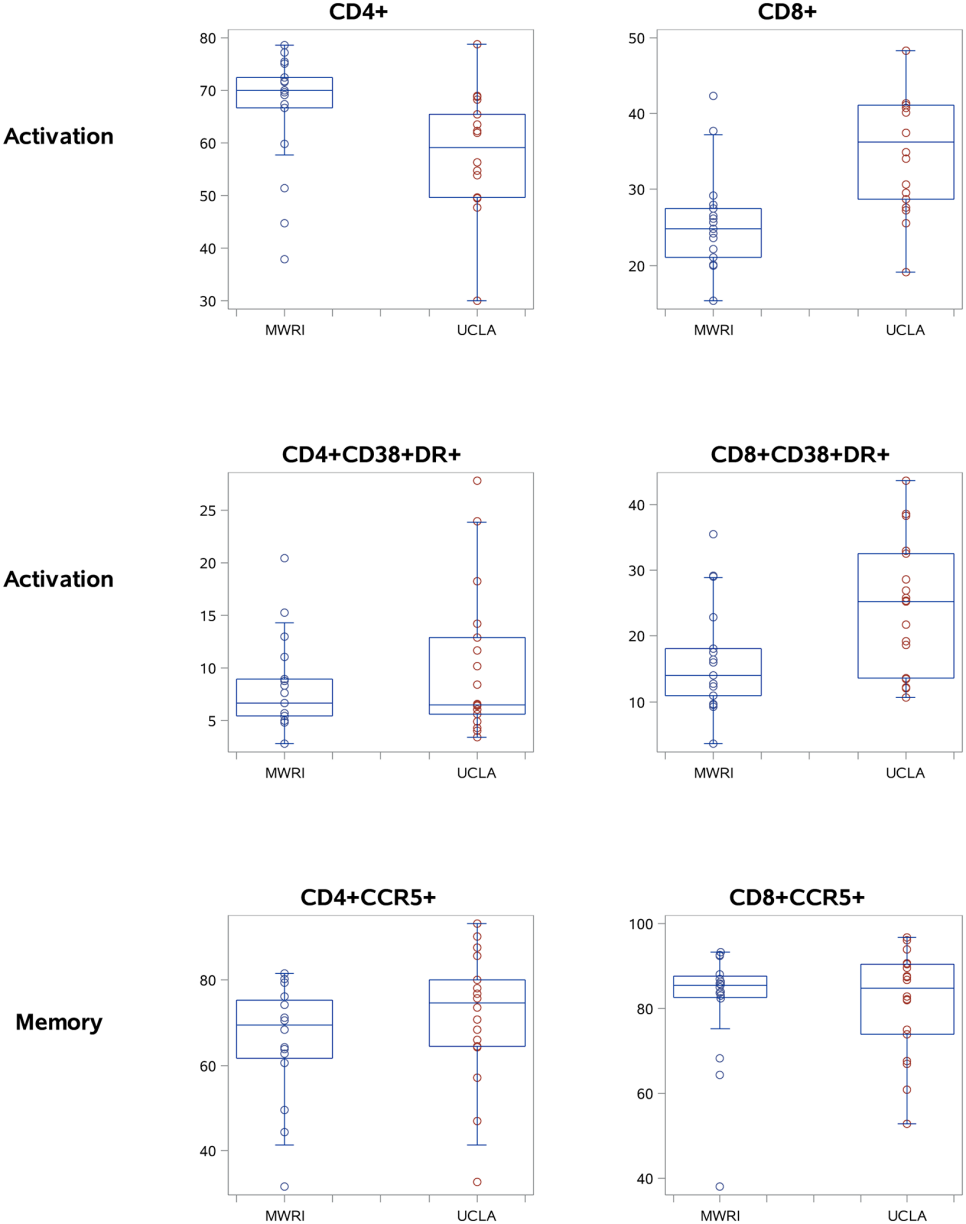
Selected MMC data for: CD4+, CD8+, CD4+CCR5+, CD8+CCR5+, CD4+CD38+DR+, and CD8+CD38DR+ T cells (boxplots showing the median, quartiles, and range for each cell population).

Graphical representations of 6 selected populations are shown in Fig 5 for PBMC and Fig 6 for MMC, respectively.

### Influence of HSV status on PBMC and MMC phenotype

As mentioned above, 6 of the 18 participants at UCLA were HSV-1 positive. A number of significant differences were noted in the activation and memory phenotype of PBMC and MMC in the HSV-1 positive group (N = 6) compared to the HSV-1 negative group (N = 12), These differences included the percentage of CD4+ or CD8+ PBMC expressing the CD27-/CD45RA+ phenotype. Differential expression of CD4+ or CD8+ MMC expressing the CD27+/CD45RA+, CD27+/CD45RA-, CD27-/CD45RA-, CD27-/CD45RA+, and CD38+/DR+ (CD4+ MMC only) was also seen. These differences are summarized in S1 and S2 Tables.

## Discussion

In this study we developed standardized protocols for cell isolation, cell staining, and flow cytometry acquisition using instruments with similar configurations. This approach resulted in data that were consistent over time within each of two laboratories allowing for template gating to be used. Flow cytometry data across the laboratories were very similar with only minor modifications needed in the gating template that was shared between the sites. Centralized analysis was used to avoid potential variability introduced by site-specific gating of the data, a structure that enabled within study identification of differences between the laboratories. Control qPBMC from a single subject were included in each experiment and provided a method for trend analysis and allowed for comparisons between sites.

Results showed that although overall CD3+, CD4+ and CD8+ T cell frequencies were comparable between sites, many of the memory and activation subsets were not. This suggests that even with careful standardization, there is limited ability to attain comparable data when samples are processed, stained and acquired at different sites. This places emphasis on the need to either arrange for centralized processing/flow cytometric acquisition or to perform concordance testing with iterative remediation to ensure data from multiple sites are comparable.

Our study clearly demonstrated that FMO controls are not necessary in each experiment or for each specimen type as long as standardized protocols are used. These standardized protocols allowed for template gates to be used and FMOs performed at the beginning of the study can be used to establish the gates that will be used throughout the study. This finding is especially important in mucosal studies since it avoids the loss of precious tissue-derived cells in FMO-tubes.

A major aim of this study was to determine whether standardized procedures performed at individual sites with centralized analysis of flow data would allow generation of PBMC and MMC data from healthy controls that were similar across two trial sites. For this pilot study, our goal was to recruit similar participants at both sites; however, differences in the study participants at each site could be the reason for statistical differences in some cell populations for PBMC and MMC. The Los Angeles participants were slightly older than the Pittsburgh participants (35.4 ± 12.5 versus 28.9 ± 9.3 years) although this difference was not significant. Additionally, the original goal of the study was to enroll a population that was seronegative for both HIV-1 and HSV-1/2. Unfortunately, this lead to a high screen/enrollment ratio at UCLA and a decision was made to drop the HSV-1/2 exclusion criterion. As a consequence, the UCLA participants included seven individuals who were HSV-1 seropositive. It is uncertain as to whether asymptomatic HSV-1 seropositivity might account for some of the observed flow cytometric differences between the two groups of participants as none of the MWRI participants were HSV-1/2 seropositive. HSV-2 activation can be associated with accumulation of CD4+/CCR5+ T cells in affected genital tissue [11]. As HSV-1 infection can be associated with proctitis in HIV-1 seronegative men who have sex with men [12], it is possible that it may also be associated with perturbation of T cell subsets in rectal tissue. When the flow cytometric data from the UCLA site were stratified by HSV-1 status there were significant differences in a number of memory and activation phenotypes. These findings emphasize the challenges associated with comparing PBMC and MMC data from participants at separate clinical trial sites.

In future studies it would be appropriate to obtain PBMC and MMC samples, divide the samples and distribute these to sites for staining and flow cytometric analysis. With this approach the inherent variability associated with study participants would be removed and the study could focus on differences that might be associated with technical aspects of the procedures. Overnight shipping of fresh mucosal specimens can be confounded by weather-associated delays, and there is also concern about the changes in cell phenotype that might be introduced by cryopreservation [13]. Further work will be needed to evaluate the impact of cryopreservation on the phenotype of cells isolated from various tissue compartments. Thus, despite the differences we observed between sites, local processing may be optimal and may even be necessary depending on whether the marker or function of interest is stable. In this case, it will be necessary to determine whether the anticipated change in the marker or function of interest in response to the intervention tested in the clinical trial is large enough to exceed the inter-site variation we observed.

There were no significant differences in the mean/median qPBMC CD4+ and CD8+ T cell percentage data between the two sites although there was significant (but stable within-laboratory) variance between the two sites (Table 3). It is reassuring that there were no significant differences between the mean/median percentages of CD4+ and CD8+ T cells between the two sites since initiatives such as the National Institute of Allergy and Infectious Diseases Division of AIDS (NIAID DAIDS) Immunology Quality Assessment (IQA) Program have demonstrated the ability of multiple North American laboratories to demonstrate proficiency in lymphocyte subset phenotyping [8]. CD4+ and CD8+ T cells are easily identified by flow cytometry, with clear separation between positive and negative cells.

We observed significant differences in qPBMC subsets of CD4+ and CD8+T cells, subsets such as CD38+, HLA-DR+, CCR5+, CD27+, and CD45RA+. These markers do not have clear separation between positive and negative cells, and thus percentage of positive cells is highly dependent on the position of the gate. These markers have not routinely been evaluated in the IQA Program, perhaps because of the more challenging gating.

Although the qPBMC inherently serve as the best standard control sample across the sites, there may be site-based variability in maintaining cryopreservation during/post shipping, storage, or thawing that may lead to qPBMC sample heterogeneity. Additionally, there may be site-specific technical variables that are not entirely accounted for in the standardized protocols such as performance of flow cytometers, isolation of cell populations, and flow cytometry staining technique. These latter variables could influence the qPBMC, PBMC, and MMC data. Thus, perhaps with additional optimization across sites and more extensive concordance testing, it may be possible to achieve improved performance across other T cell subsets. Small comparative studies with iterative protocol modifications, perhaps using only qPBMC, may be needed to achieve concordance between sites. This likely requires a major investment in time and resources. Our study only included two sites already expert in isolating, staining, gating and analyzing colorectal mucosal biopsy samples. Harmonizing sites with less experience of mucosal flow cytometry would be even more complex.

The use of qPBMC within any one site effectively tracks performance over time and highlights samples producing outlier results with likely invalid data. In this study, further investigation into excluded data exposed irregularities in some aspect of the cell staining or collection on the cytometer demonstrating that the use of standard cells included in each experiment and trended over time is a useful quality-control measure to identify flow data that may be compromised. This type of control should be considered in any type of longitudinal study, and pre-established acceptability criteria can use used to exclude potentially unreliable data. Standardized procedures and instrument set-up allow for template gating and remove the need to perform FMO staining controls within each experiment. If the expectation is to perform processing/data acquisition at multiple sites, then it is important to plan for sufficient time and effort needed to achieve concordance between sites. Alternatively, cryopreservation techniques may need to be developed, distinct from those for PBMC, and especially designed for mucosal samples (MMC) to allow for shipment to a centralized testing facility without loss or shift in cellular phenotype. As the number of multi-center trials collecting cellular samples is likely to increase there is an urgent need to optimize the techniques used to analyze these samples.

## Supporting Information

S1 Fig Fluorescence-minus-one (FMO) controls for the memory panel. Shown is an example from one experiment at one site. The upper graphs show the FMO for PE CCR5, the middle for APC CD27, and the lower for FITC CD45RA. The three specimen types are shown with MMC on the left, PBMC in the middle and qPBMC on the right. For each, graphs are paired with the full stain on the left and the FMO on the right. Note that the FMO defines the lower limit of the gate; often the gate is placed higher.
(PDF)

S2 Fig Levey-Jennings plots for all cellular subsets. The bold black line shows the mean, and the dotted red line shows +/- 3 SD from the mean. Green and yellow lines are for 1 and 2 SD.
(PDF)

S1 File Study protocol. (PDF)

S1 Table PBMC comparisons stratified by HSV-1 status. (DOCX)

S2 Table MMC comparisons stratified by HSV. (DOCX)
