# Supplementary figures and images for: Exploring the Feasibility of Multi-Site Flow Cytometric Processing of Gut Associated Lymphoid Tissue with Centralized Data Analysis for Multi-Site Clinical Trials
Source: PLoS One. 2015 May 26;10(5):e0126454. doi: 10.1371/journal.pone.0126454 (PMC4444258; doi:10.1371/journal.pone.0126454)

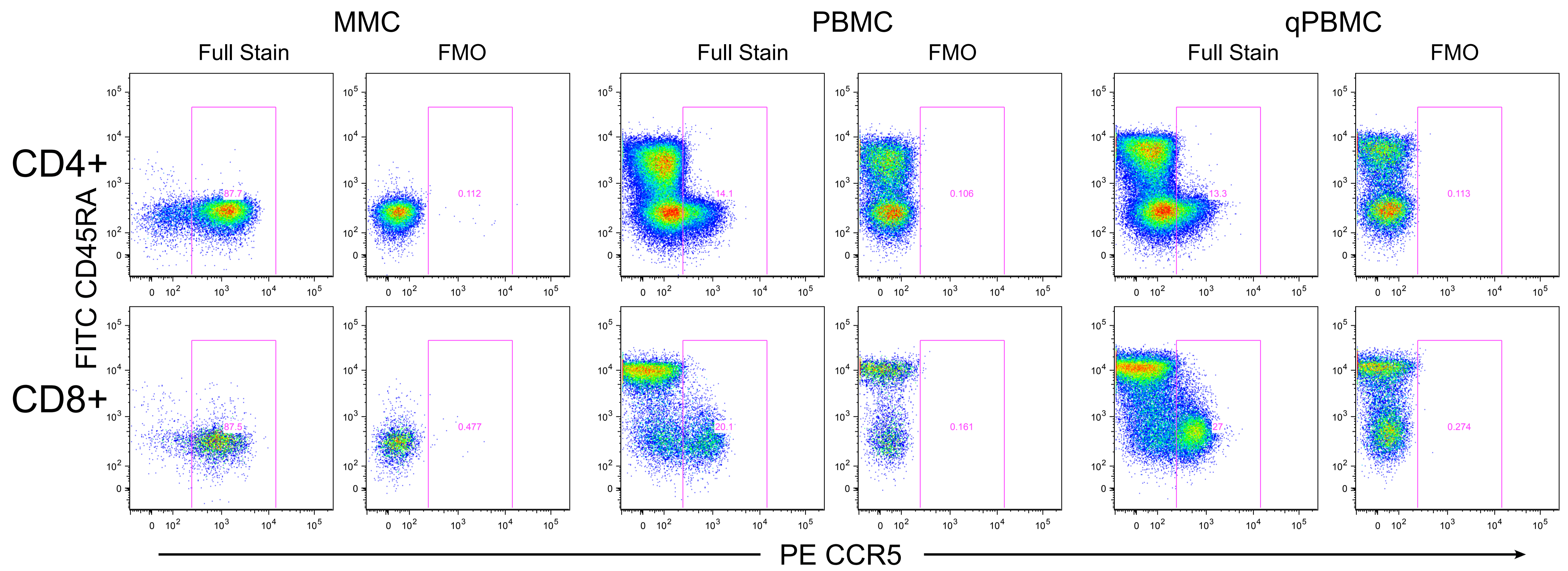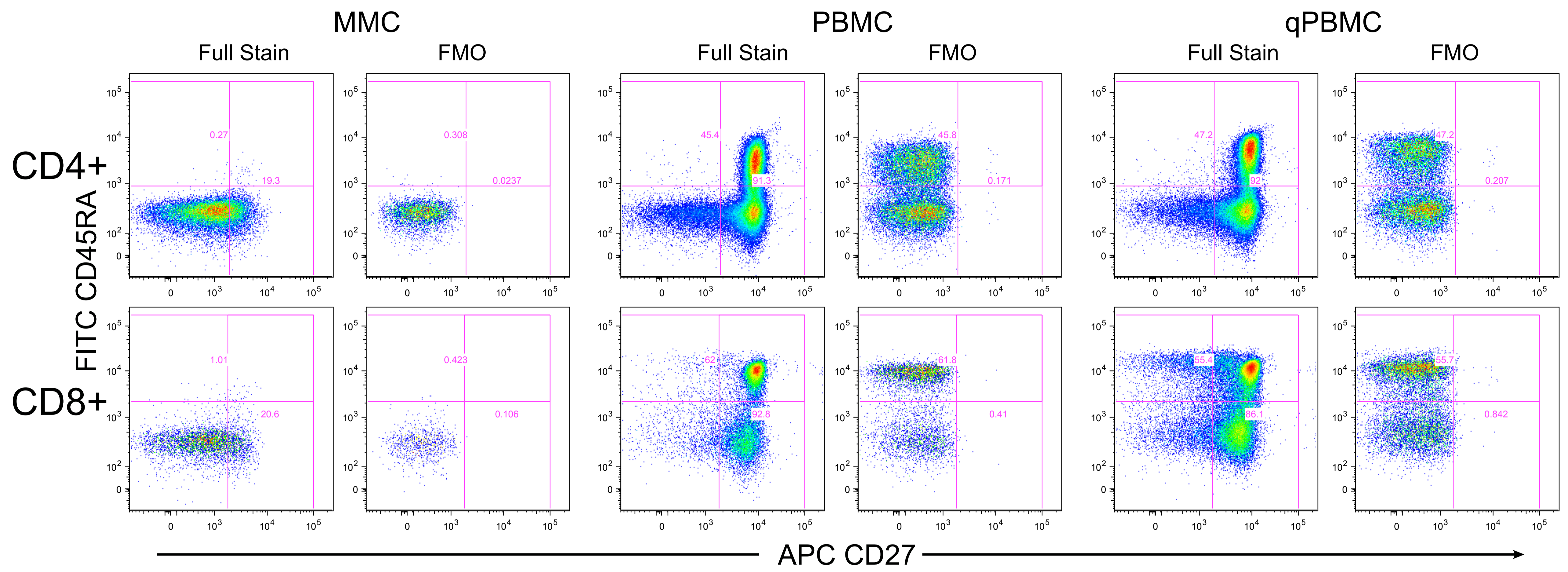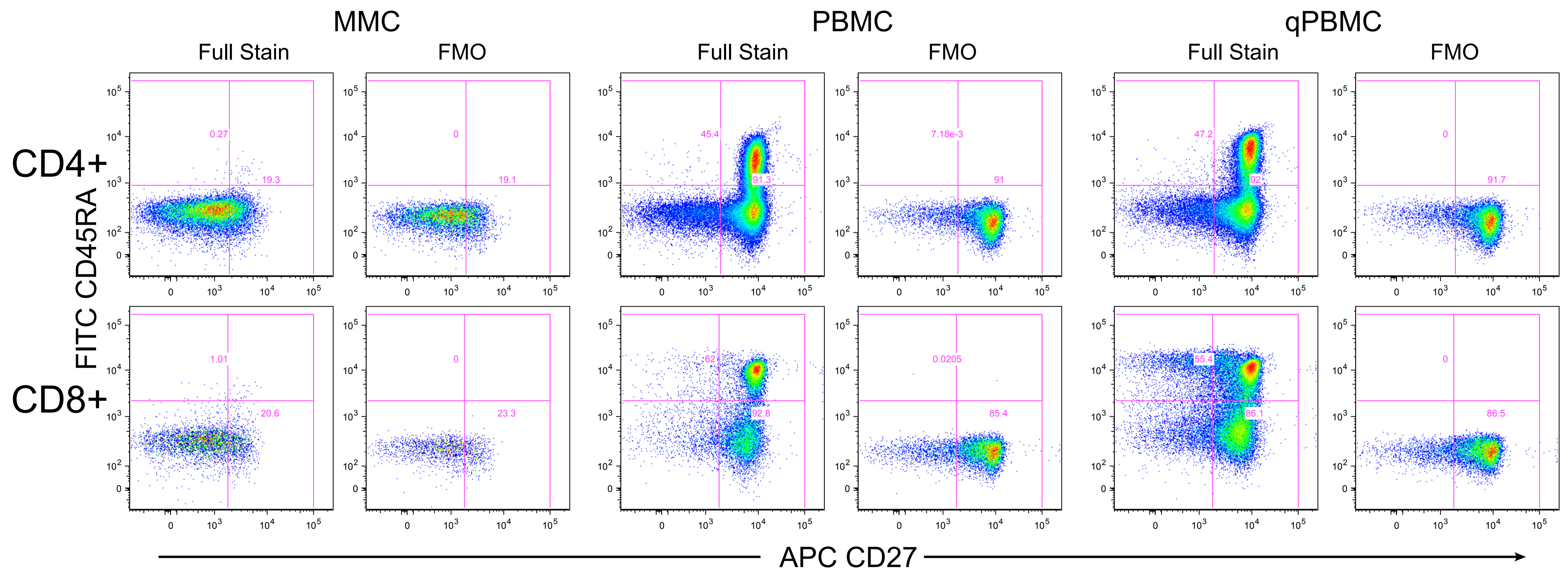

Supplement: S1 Fig — Shown is an example from one experiment at one site. The upper graphs show the FMO for PE CCR5, the middle for APC CD27, and the lower for FITC CD45RA. The three specimen types are shown with MMC on the left, PBMC in the middle and qPBMC on the right. For each, graphs are paired with the full stain on the left and the FMO on the right. Note that the FMO defines the lower limit of the gate; often the gate is placed higher. (PDF) [file pone.0126454.s001.pdf]
