# Supplementary material for: Exploring the Feasibility of Multi-Site Flow Cytometric Processing of Gut Associated Lymphoid Tissue with Centralized Data Analysis for Multi-Site Clinical Trials
Source: PLoS One. 2015 May 26;10(5):e0126454. doi: 10.1371/journal.pone.0126454 (PMC4444258; doi:10.1371/journal.pone.0126454)
Supplement: S2 Fig — The bold black line shows the mean, and the dotted red line shows +/- 3 SD from the mean. Green and yellow lines are for 1 and 2 SD. (PDF) [file pone.0126454.s002.pdf]

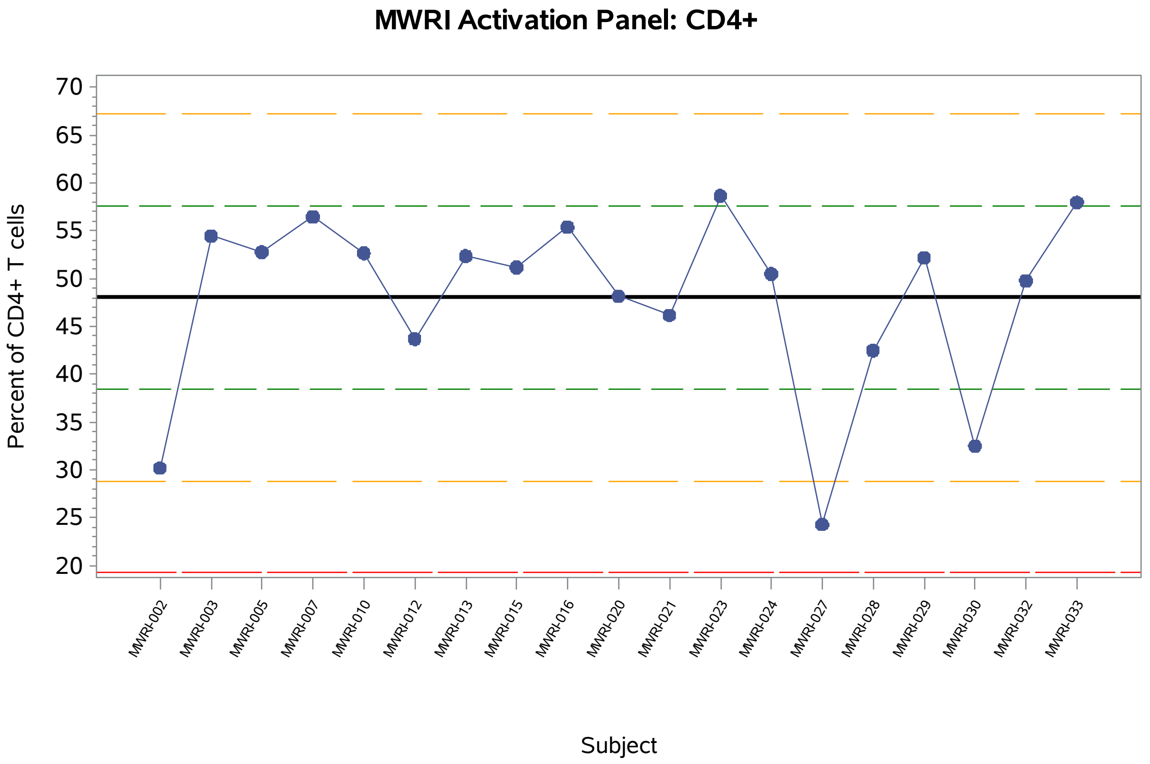
MWRI Activation Panel: CD4+
Percent of CD4+ T cells
70
65
60
55
50
45
40
35
30
25
20
MWRI-002
MWRI-003
MWRI-005
MWRI-007
MWRI-010
MWRI-012
MWRI-013
MWRI-015
MWRI-016
MWRI-020
MWRI-021
MWRI-023
MWRI-024
MWRI-027
MWRI-028
MWRI-029
MWRI-030
MWRI-032
MWRI-033
Subject

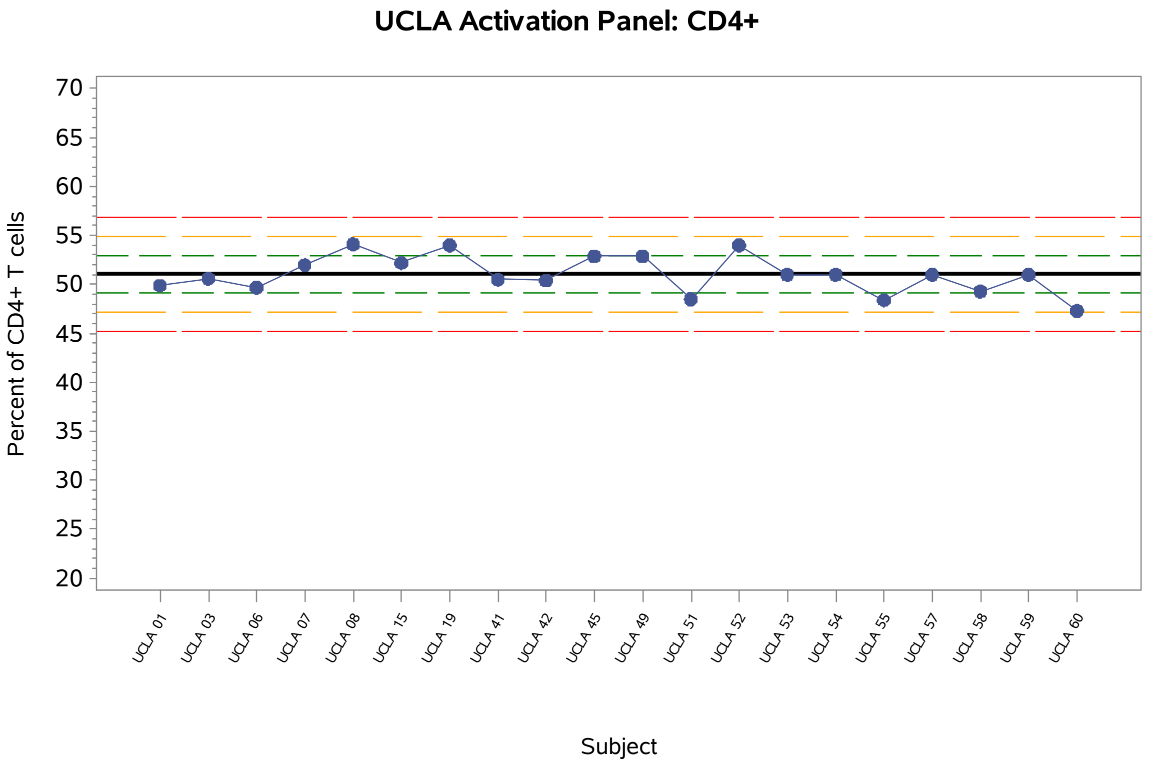
UCLA Activation Panel: CD4+
Percent of CD4+ T cells
70
65
60
55
50
45
40
35
30
25
20
UCLA 01
UCLA 03
UCLA 06
UCLA 07
UCLA 08
UCLA 15
UCLA 19
UCLA 41
UCLA 42
UCLA 45
UCLA 49
UCLA 51
UCLA 52
UCLA 53
UCLA 54
UCLA 55
UCLA 57
UCLA 58
UCLA 59
UCLA 60
Subject

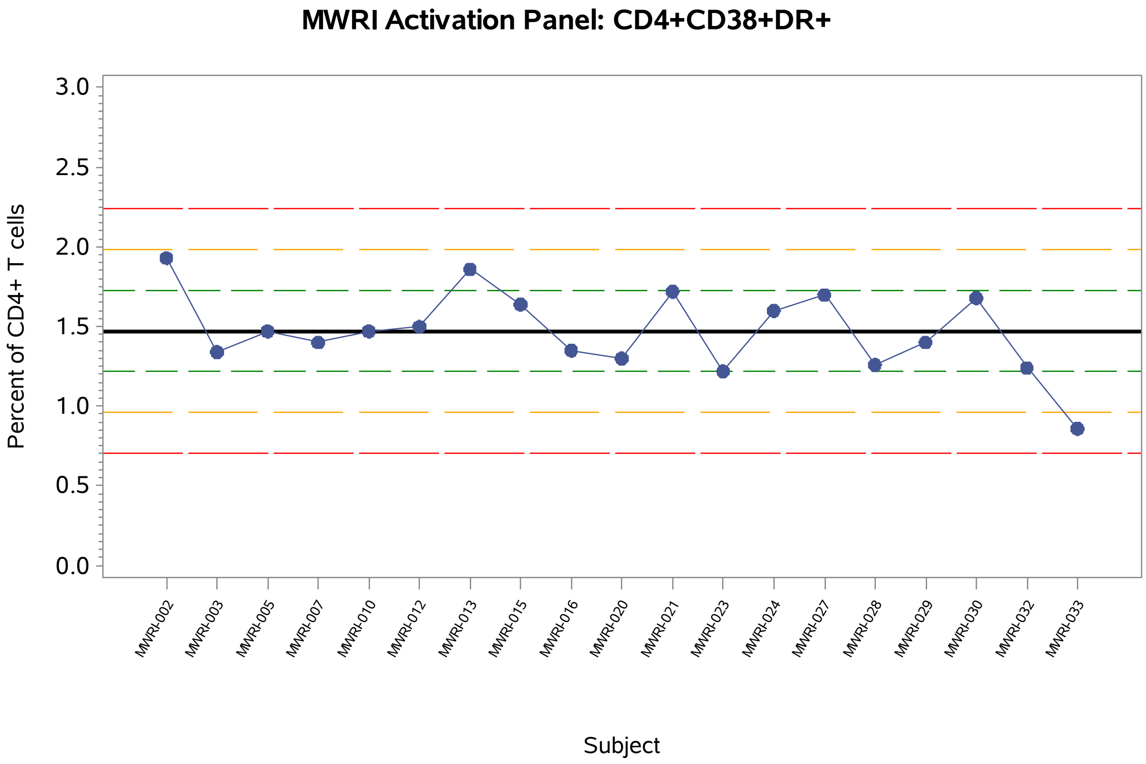
MWRI Activation Panel: CD4+CD38+DR+
Percent of CD4+ T cells
3.0
2.5
2.0
1.5
1.0
0.5
0.0
MWRI-002
MWRI-003
MWRI-005
MWRI-007
MWRI-010
MWRI-012
MWRI-013
MWRI-015
MWRI-016
MWRI-020
MWRI-021
MWRI-023
MWRI-024
MWRI-027
MWRI-028
MWRI-029
MWRI-030
MWRI-032
MWRI-033
Subject

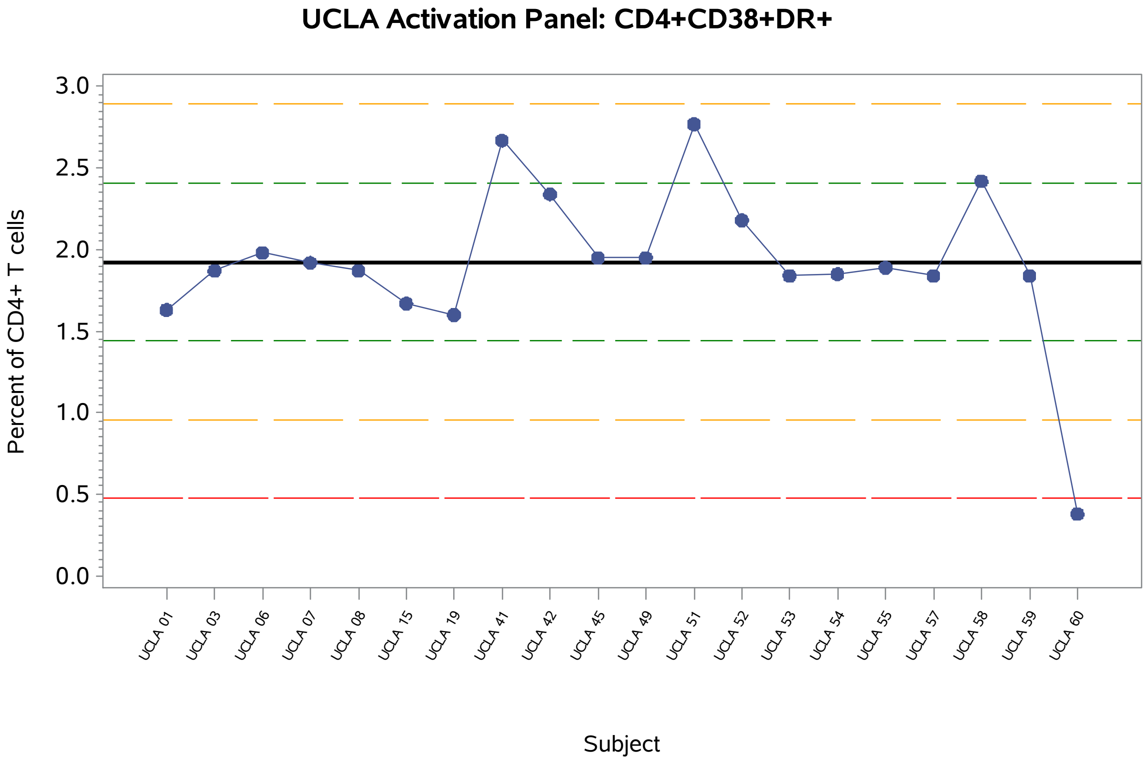
UCLA Activation Panel: CD4+CD38+DR+
Percent of CD4+ T cells
3.0
2.5
2.0
1.5
1.0
0.5
0.0
UCLA 01
UCLA 03
UCLA 06
UCLA 07
UCLA 08
UCLA 15
UCLA 19
UCLA 41
UCLA 42
UCLA 45
UCLA 49
UCLA 51
UCLA 52
UCLA 53
UCLA 54
UCLA 55
UCLA 57
UCLA 58
UCLA 59
UCLA 60
Subject

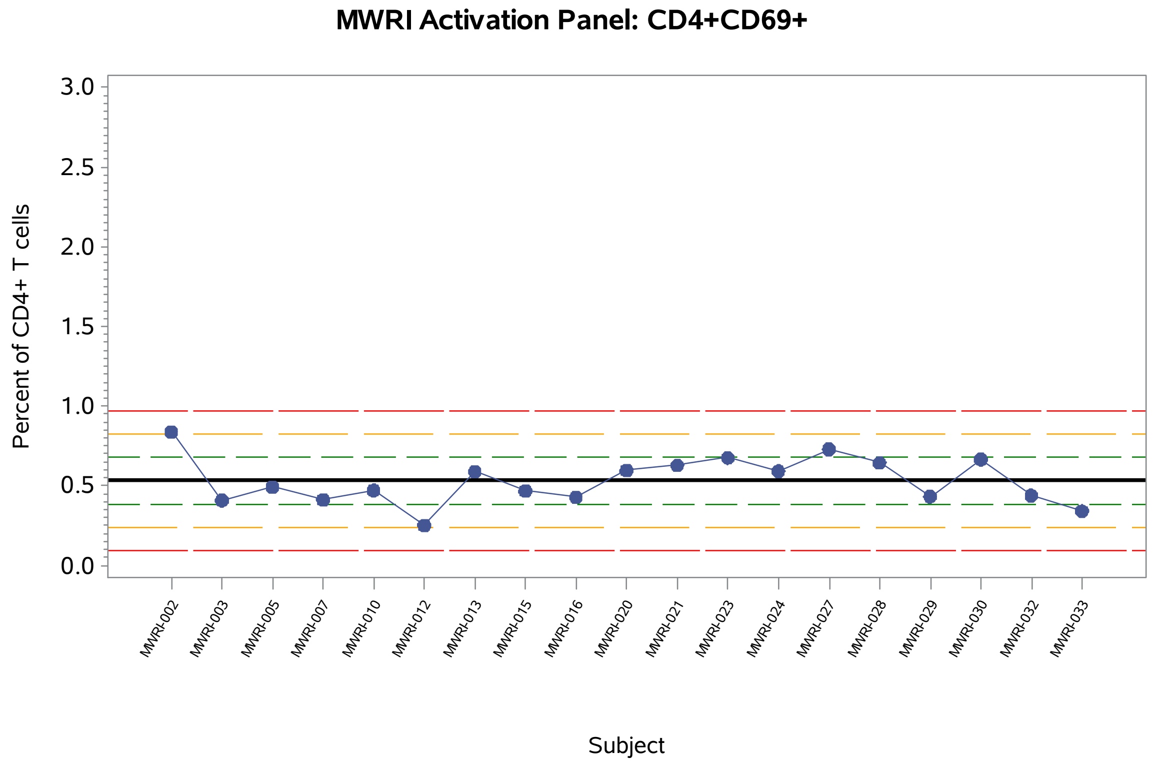
MWRI Activation Panel: CD4+CD69+
Percent of CD4+ T cells
3.0
2.5
2.0
1.5
1.0
0.5
0.0
MWRI-002
MWRI-003
MWRI-005
MWRI-007
MWRI-010
MWRI-012
MWRI-013
MWRI-015
MWRI-016
MWRI-020
MWRI-021
MWRI-023
MWRI-024
MWRI-027
MWRI-028
MWRI-029
MWRI-030
MWRI-032
MWRI-033
Subject

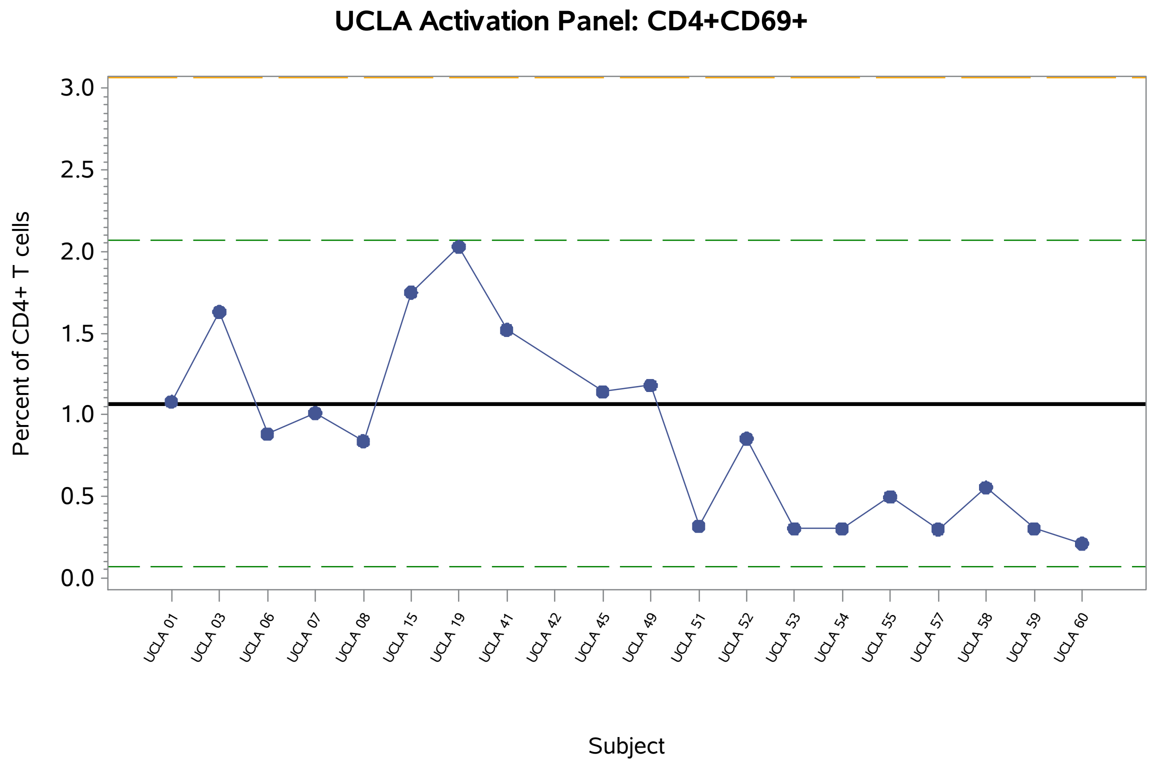
UCLA Activation Panel: CD4+CD69+
Percent of CD4+ T cells
3.0
2.5
2.0
1.5
1.0
0.5
0.0
UCLA 01
UCLA 03
UCLA 06
UCLA 07
UCLA 08
UCLA 15
UCLA 19
UCLA 41
UCLA 42
UCLA 45
UCLA 49
UCLA 51
UCLA 52
UCLA 53
UCLA 54
UCLA 55
UCLA 57
UCLA 58
UCLA 59
UCLA 60
Subject

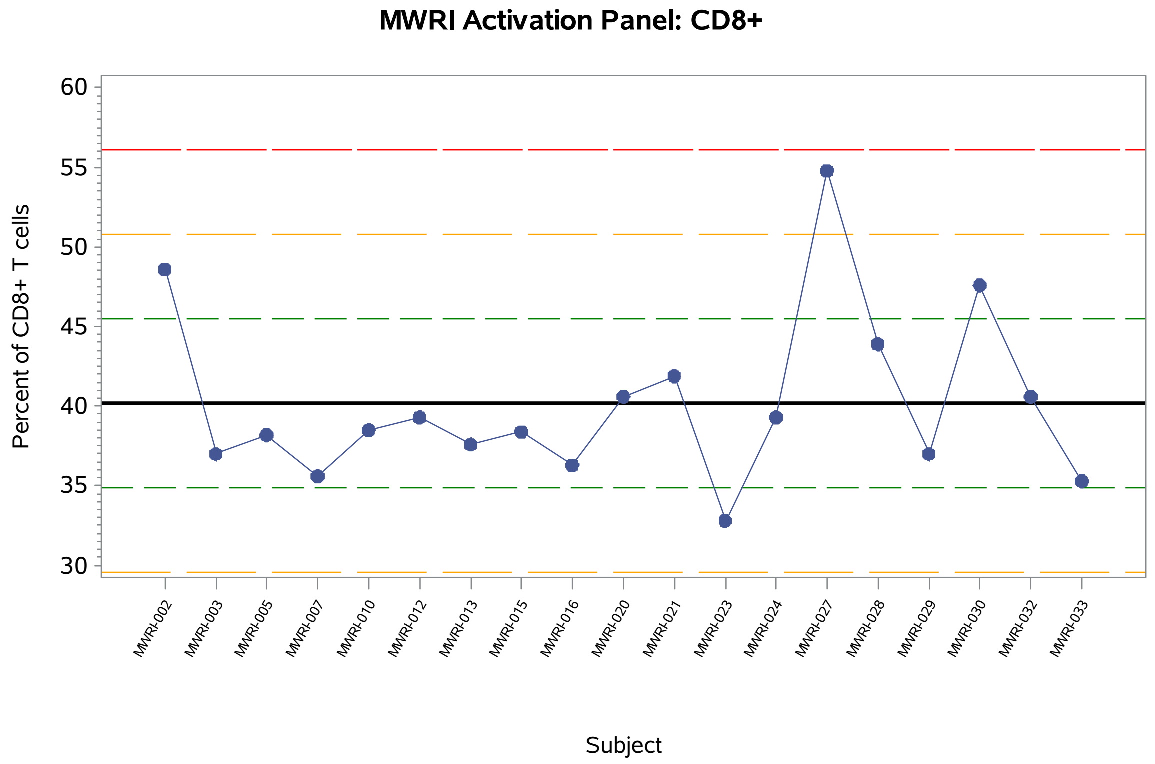
MWRI Activation Panel: CD8+
Percent of CD8+ T cells
60
55
50
45
40
35
30
MWRI-002
MWRI-003
MWRI-005
MWRI-007
MWRI-010
MWRI-012
MWRI-013
MWRI-015
MWRI-016
MWRI-020
MWRI-021
MWRI-023
MWRI-024
MWRI-027
MWRI-028
MWRI-029
MWRI-030
MWRI-032
MWRI-033
Subject

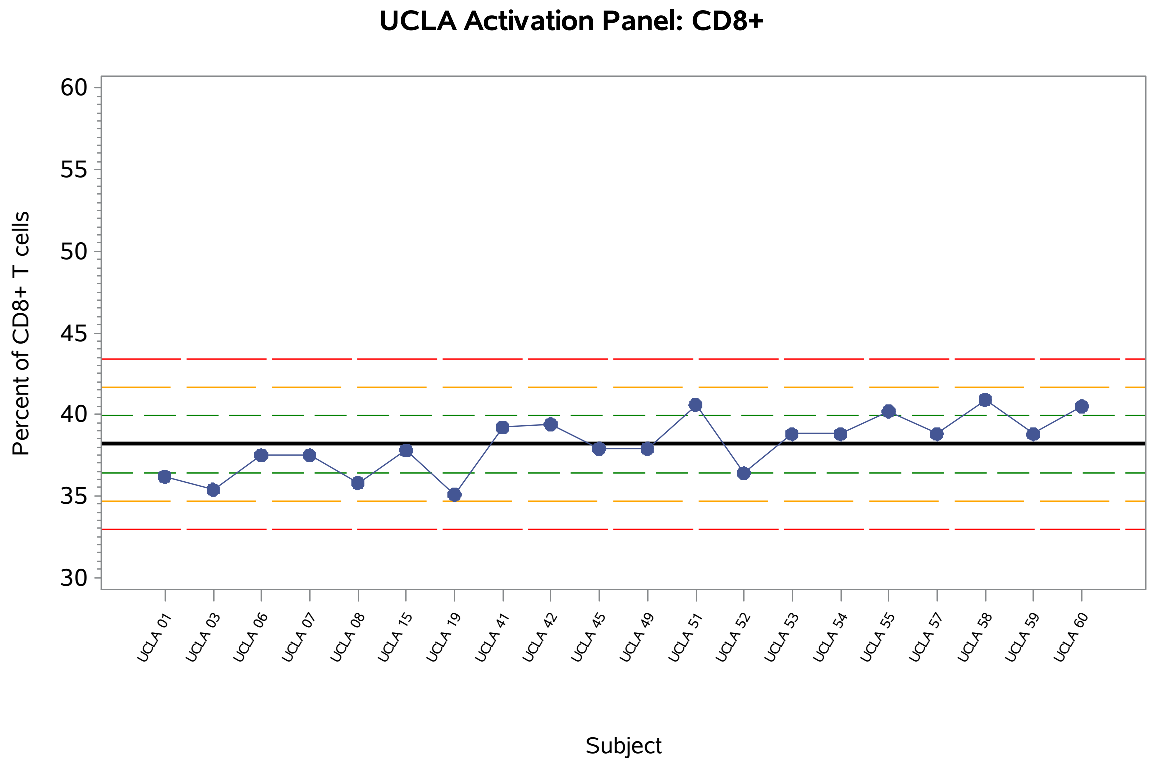
UCLA Activation Panel: CD8+
Percent of CD8+ T cells
60
55
50
45
40
35
30
UCLA 01
UCLA 03
UCLA 06
UCLA 07
UCLA 08
UCLA 15
UCLA 19
UCLA 41
UCLA 42
UCLA 45
UCLA 49
UCLA 51
UCLA 52
UCLA 53
UCLA 54
UCLA 55
UCLA 57
UCLA 58
UCLA 59
UCLA 60
Subject

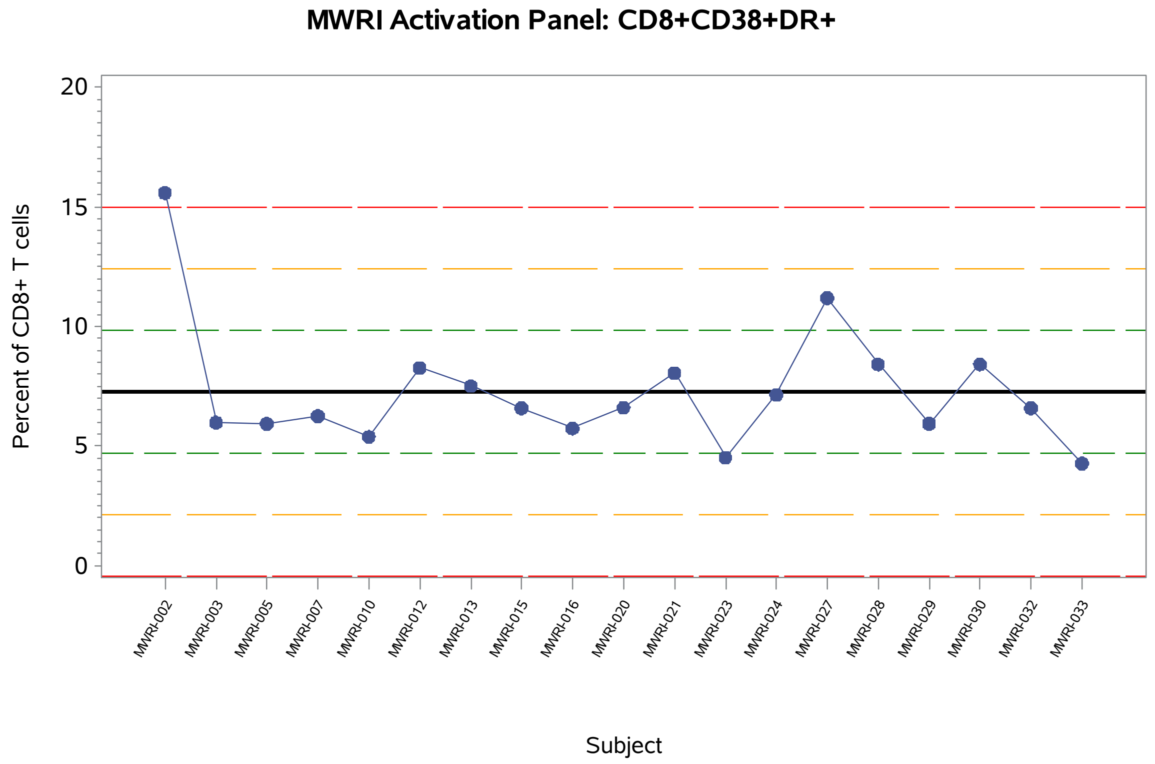
MWRI Activation Panel: CD8+CD38+DR+
Percent of CD8+ T cells
20
15
10
5
0
MWRI-002
MWRI-003
MWRI-005
MWRI-007
MWRI-010
MWRI-012
MWRI-013
MWRI-015
MWRI-016
MWRI-020
MWRI-021
MWRI-023
MWRI-024
MWRI-027
MWRI-028
MWRI-029
MWRI-030
MWRI-032
MWRI-033
Subject

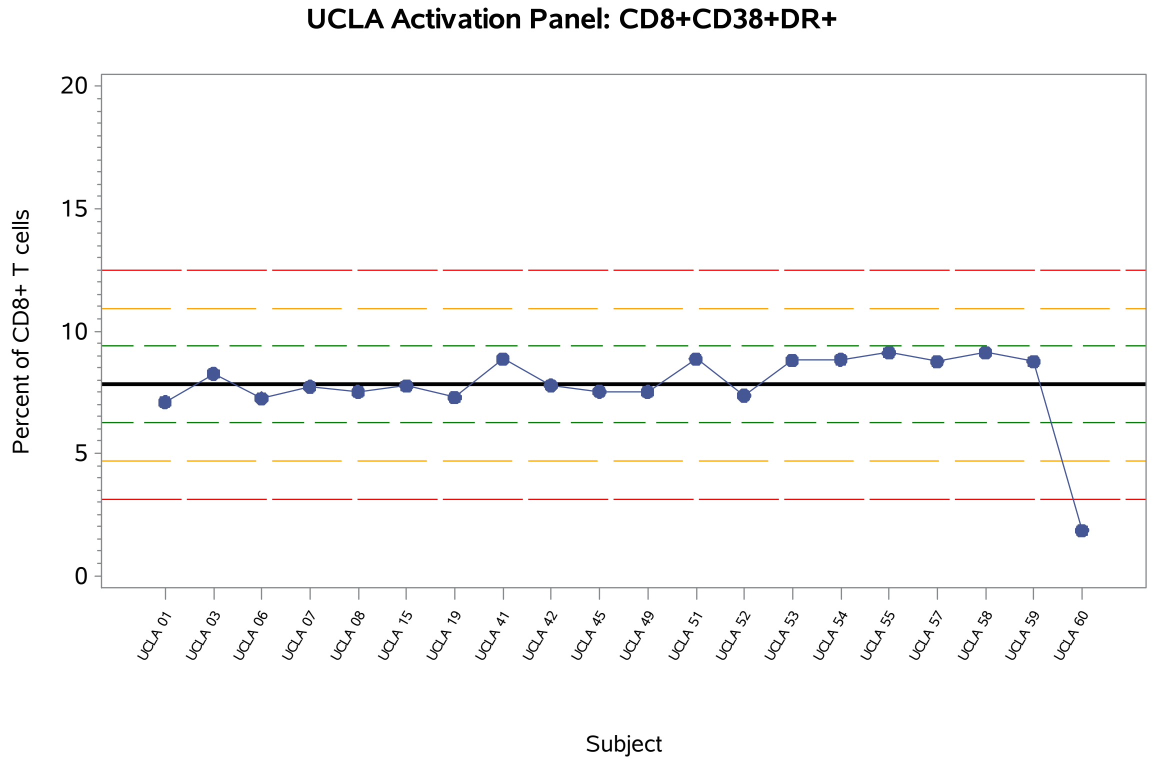
UCLA Activation Panel: CD8+CD38+DR+
Percent of CD8+ T cells
20
15
10
5
0
UCLA 01
UCLA 03
UCLA 06
UCLA 07
UCLA 08
UCLA 15
UCLA 19
UCLA 41
UCLA 42
UCLA 45
UCLA 49
UCLA 51
UCLA 52
UCLA 53
UCLA 54
UCLA 55
UCLA 57
UCLA 58
UCLA 59
UCLA 60
Subject

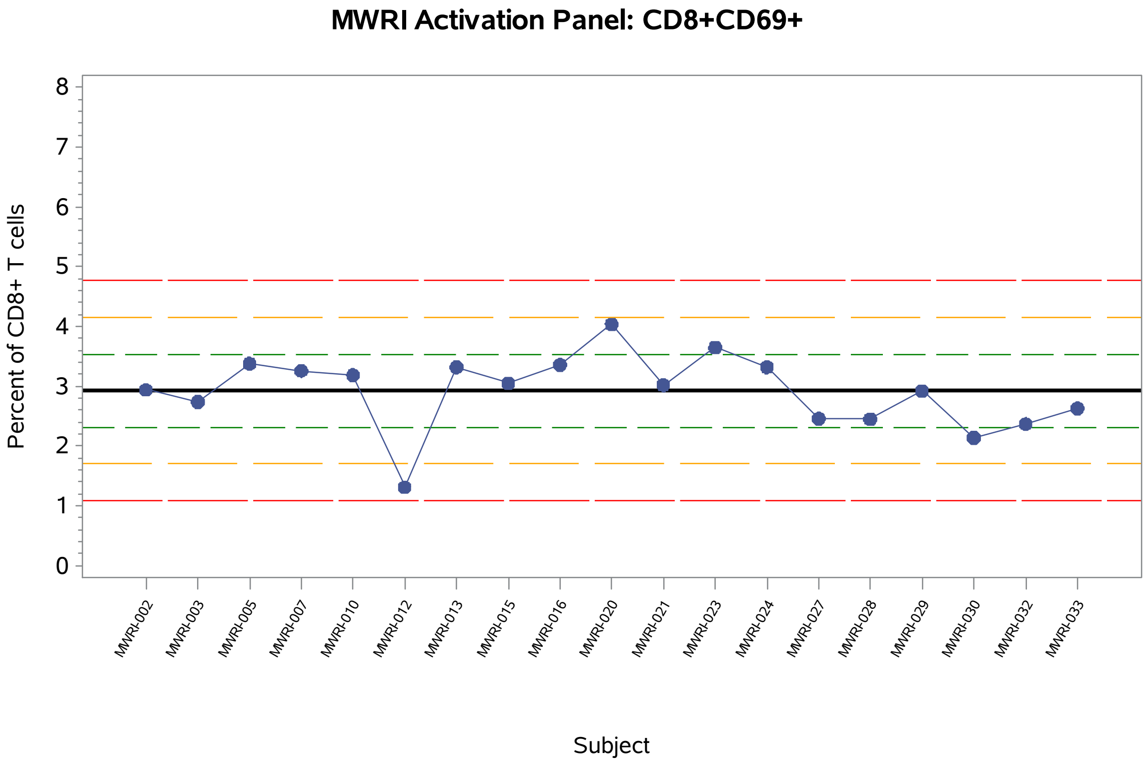
MWRI Activation Panel: CD8+CD69+
Percent of CD8+ T cells
0
1
2
3
4
5
6
7
8
MWRI-002
MWRI-003
MWRI-005
MWRI-007
MWRI-010
MWRI-012
MWRI-013
MWRI-015
MWRI-016
MWRI-020
MWRI-021
MWRI-023
MWRI-024
MWRI-027
MWRI-028
MWRI-029
MWRI-030
MWRI-032
MWRI-033
Subject

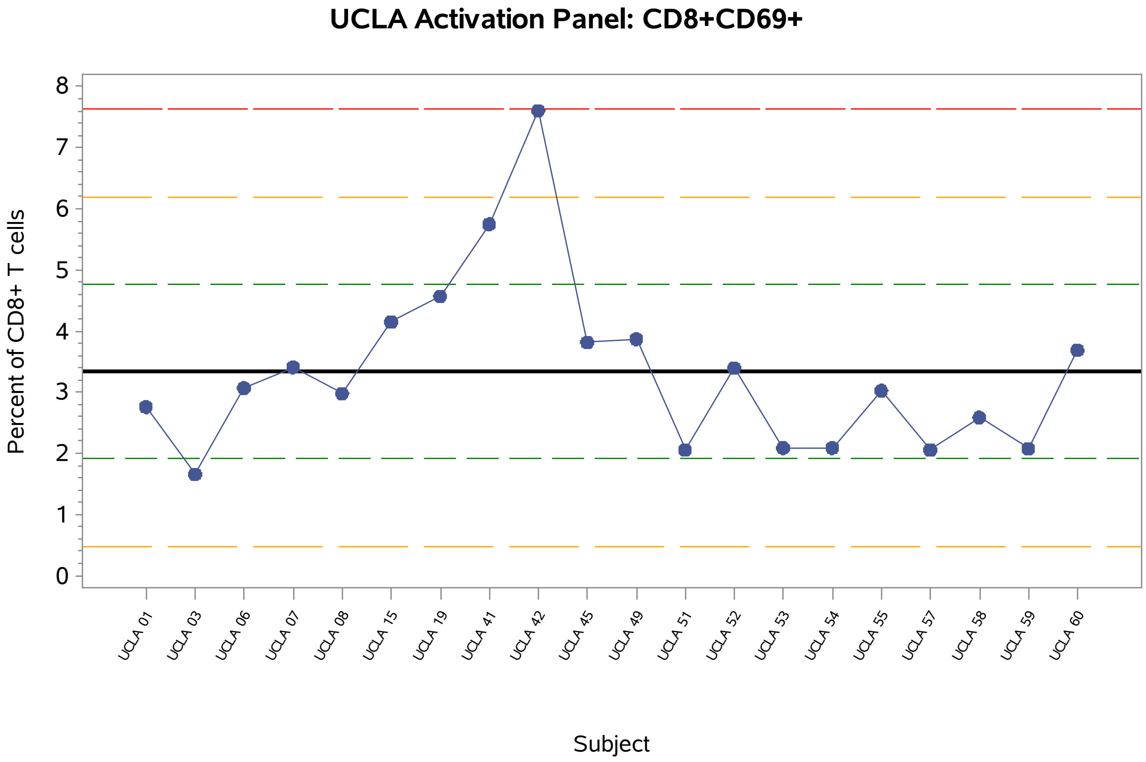
UCLA Activation Panel: CD8+CD69+
Percent of CD8+ T cells
0
1
2
3
4
5
6
7
8
UCLA 01
UCLA 03
UCLA 06
UCLA 07
UCLA 08
UCLA 15
UCLA 19
UCLA 41
UCLA 42
UCLA 45
UCLA 49
UCLA 51
UCLA 52
UCLA 53
UCLA 54
UCLA 55
UCLA 57
UCLA 58
UCLA 59
UCLA 60
Subject

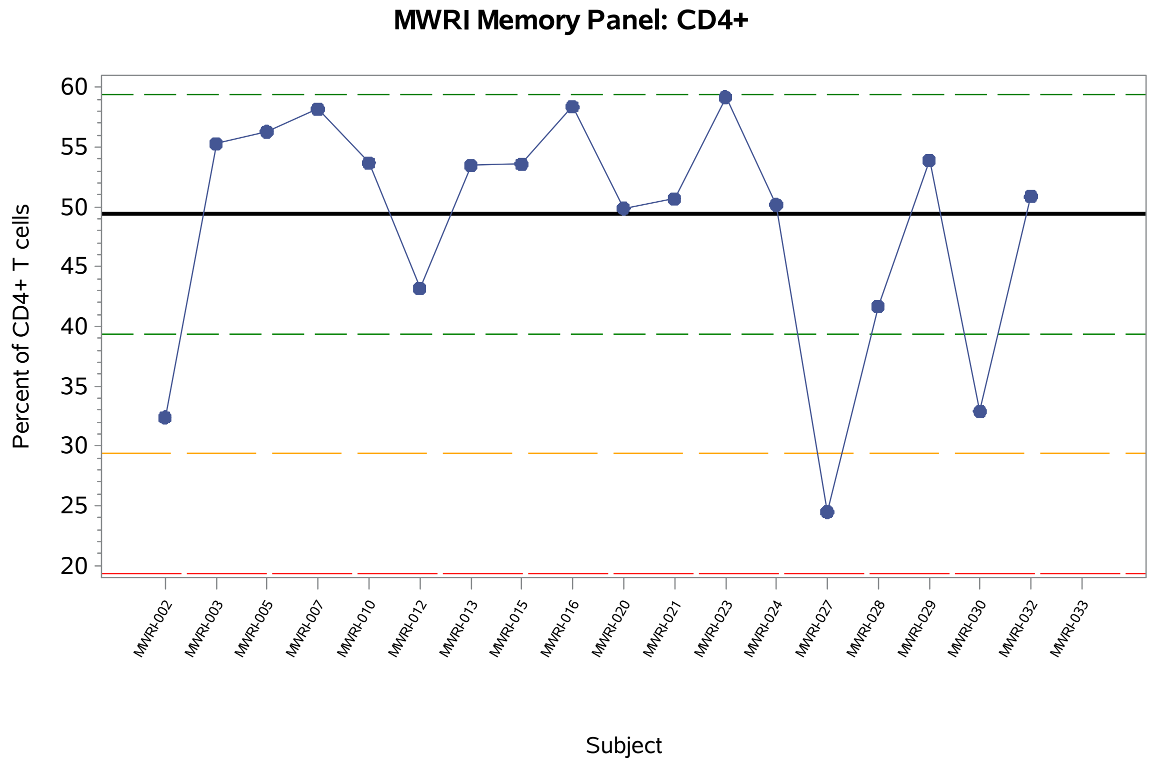
MWRI Memory Panel: CD4+
Percent of CD4+ T cells
60
55
50
45
40
35
30
25
20
MWRI-002
MWRI-003
MWRI-005
MWRI-007
MWRI-010
MWRI-012
MWRI-013
MWRI-015
MWRI-016
MWRI-020
MWRI-021
MWRI-023
MWRI-024
MWRI-027
MWRI-028
MWRI-029
MWRI-030
MWRI-032
MWRI-033
Subject

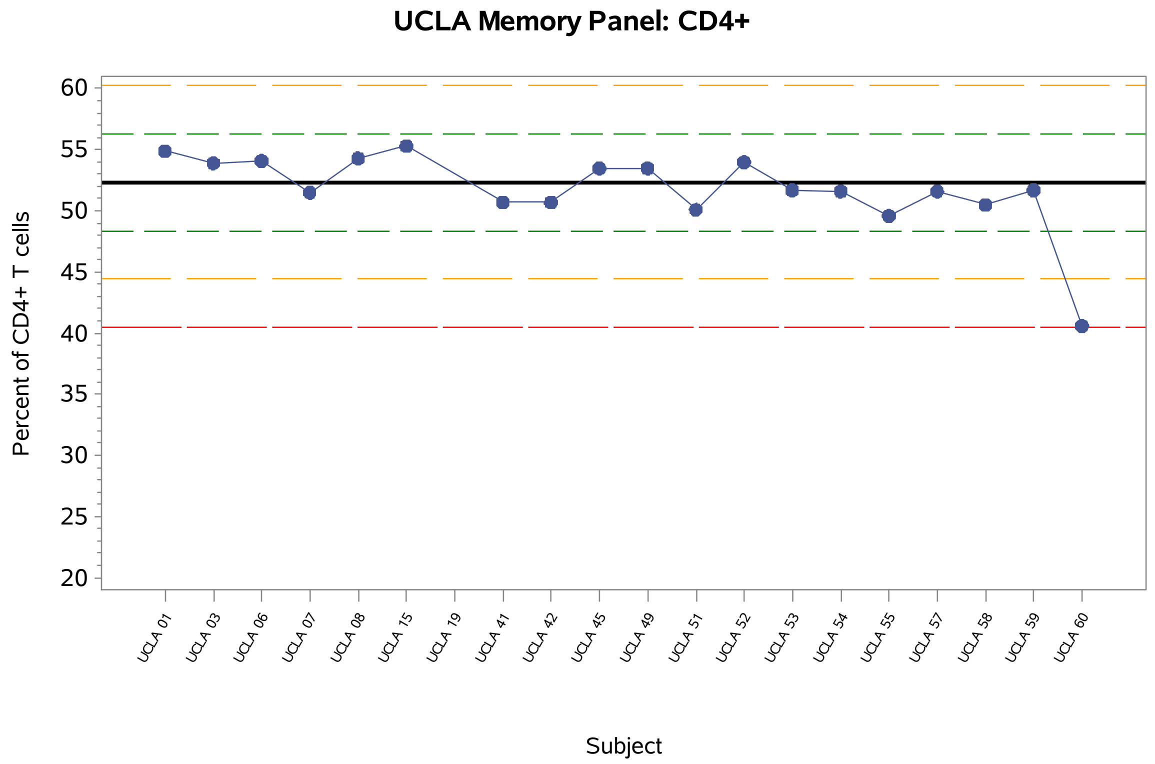
UCLA Memory Panel: CD4+
Percent of CD4+ T cells
60
55
50
45
40
35
30
25
20
UCLA 01
UCLA 03
UCLA 06
UCLA 07
UCLA 08
UCLA 15
UCLA 19
UCLA 41
UCLA 42
UCLA 45
UCLA 49
UCLA 51
UCLA 52
UCLA 53
UCLA 54
UCLA 55
UCLA 57
UCLA 58
UCLA 59
UCLA 60
Subject

MWRI Memory Panel: CD4+CCR5+

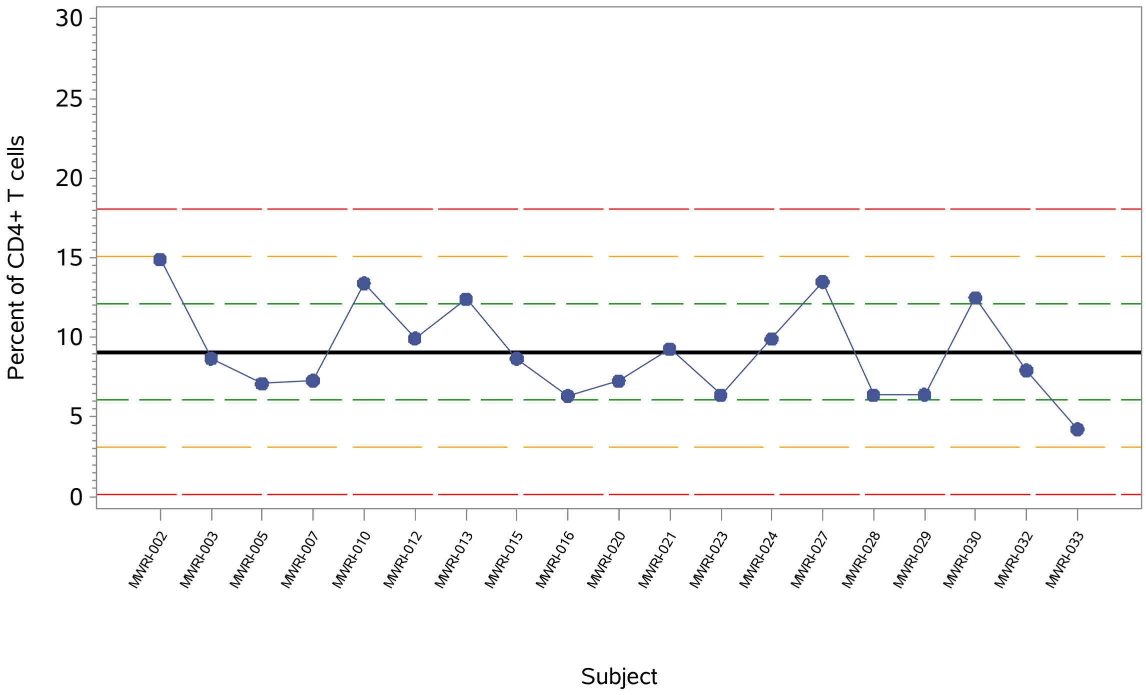

UCLA Memory Panel: CD4+CCR5+

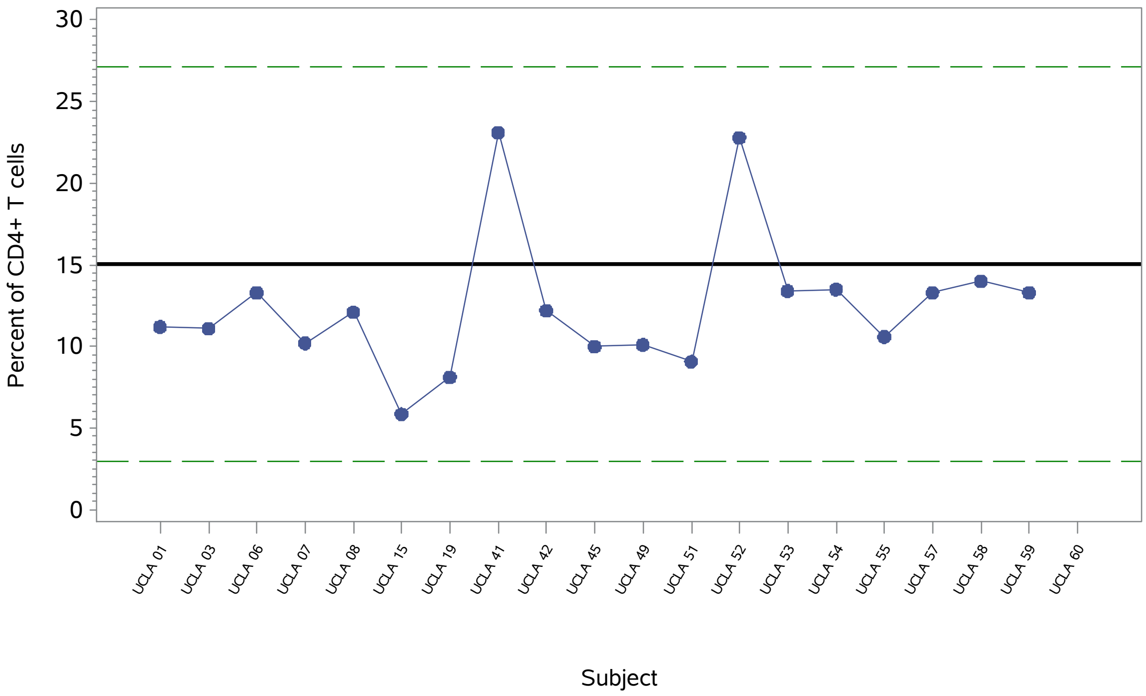

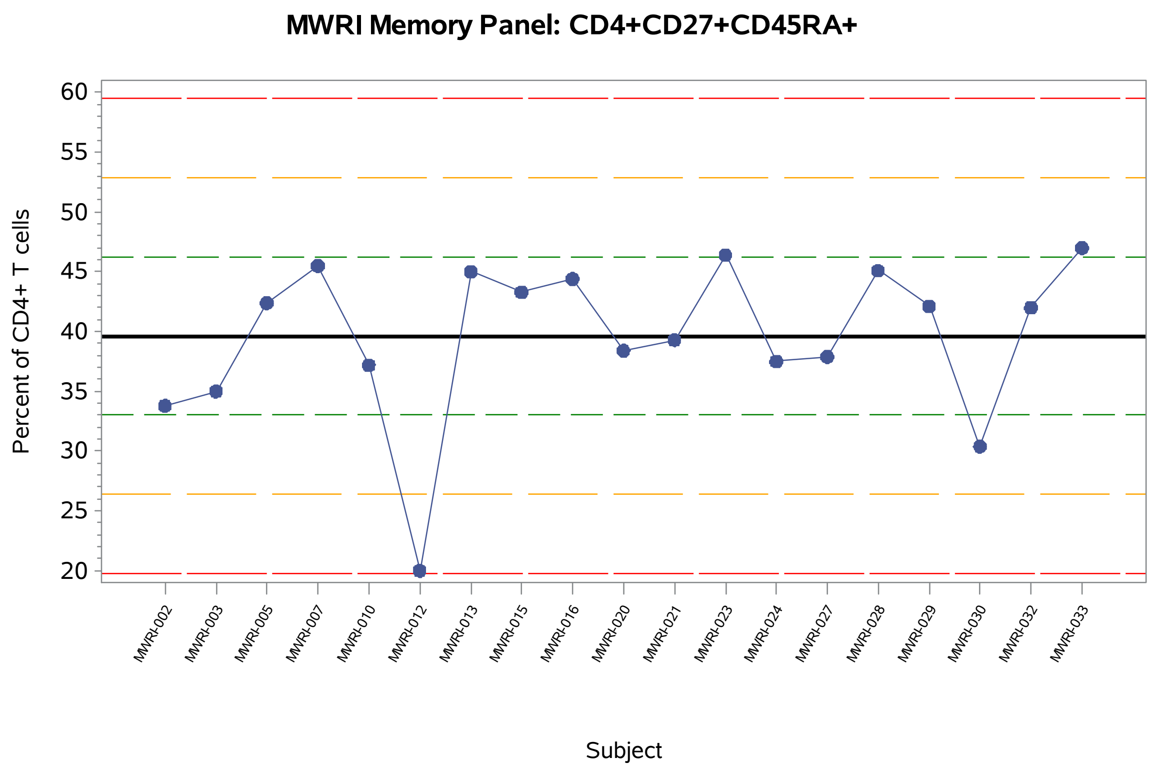
MWRI Memory Panel: CD4+CD27+CD45RA+
Percent of CD4+ T cells
60
55
50
45
40
35
30
25
20
MWRI-002
MWRI-003
MWRI-005
MWRI-007
MWRI-010
MWRI-012
MWRI-013
MWRI-015
MWRI-016
MWRI-020
MWRI-021
MWRI-023
MWRI-024
MWRI-027
MWRI-028
MWRI-029
MWRI-030
MWRI-032
MWRI-033
Subject

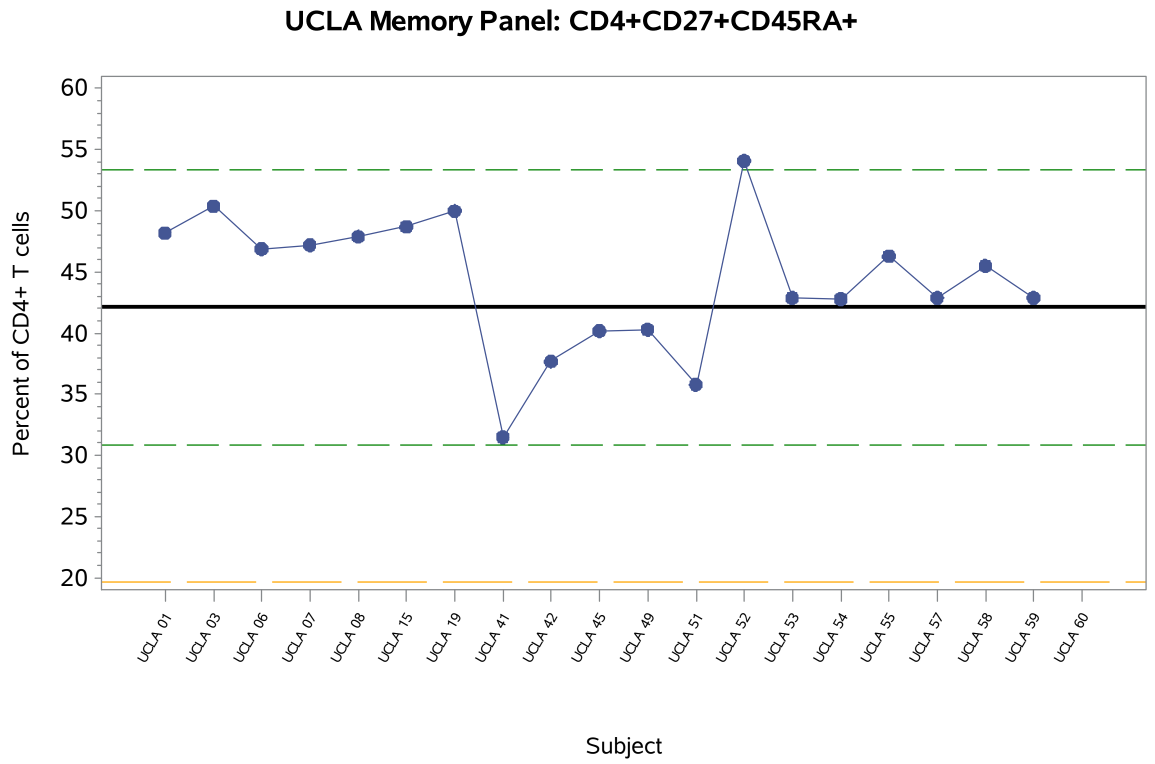
UCLA Memory Panel: CD4+CD27+CD45RA+
Percent of CD4+ T cells
60
55
50
45
40
35
30
25
20
UCLA 01
UCLA 03
UCLA 06
UCLA 07
UCLA 08
UCLA 15
UCLA 19
UCLA 41
UCLA 42
UCLA 45
UCLA 49
UCLA 51
UCLA 52
UCLA 53
UCLA 54
UCLA 55
UCLA 57
UCLA 58
UCLA 59
UCLA 60
Subject

MWRI Memory Panel: CD4+CD27+CD45RA-

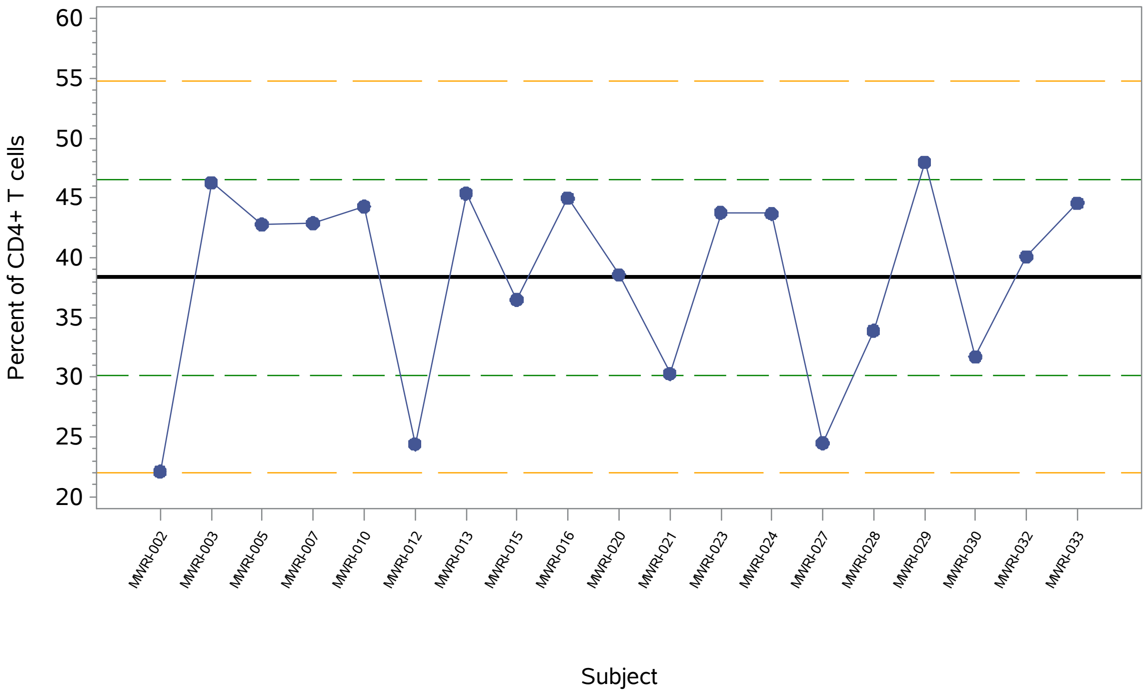

UCLA Memory Panel: CD4+CD27+CD45RA-

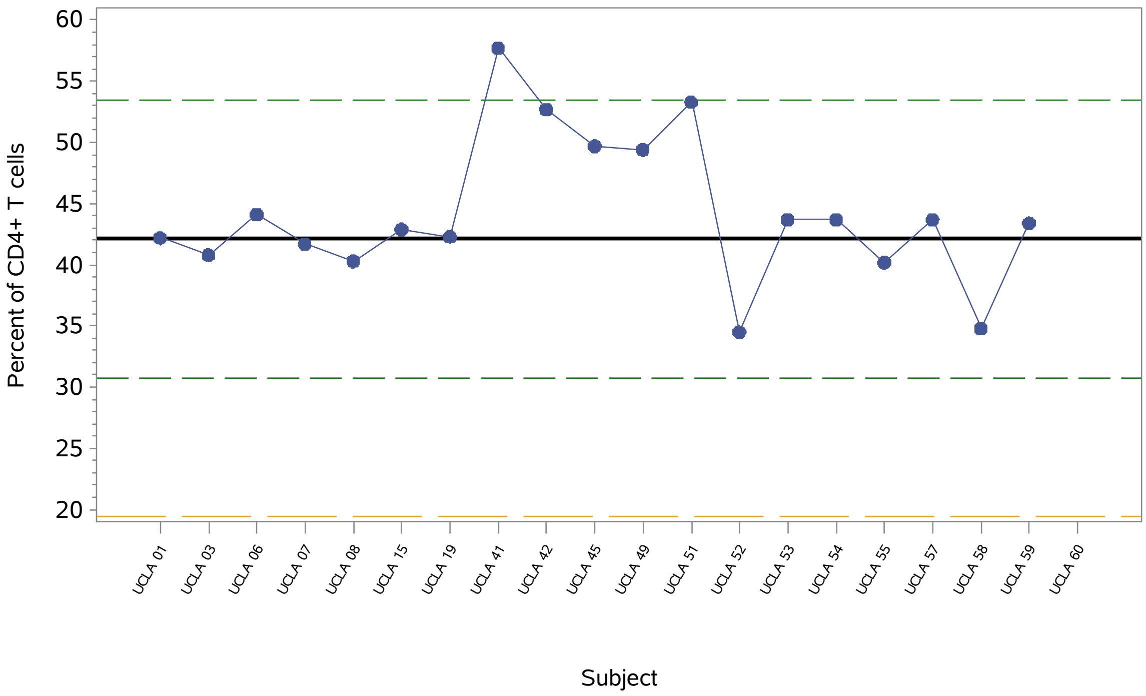

MWRI Memory Panel: CD4+CD27-CD45RA+

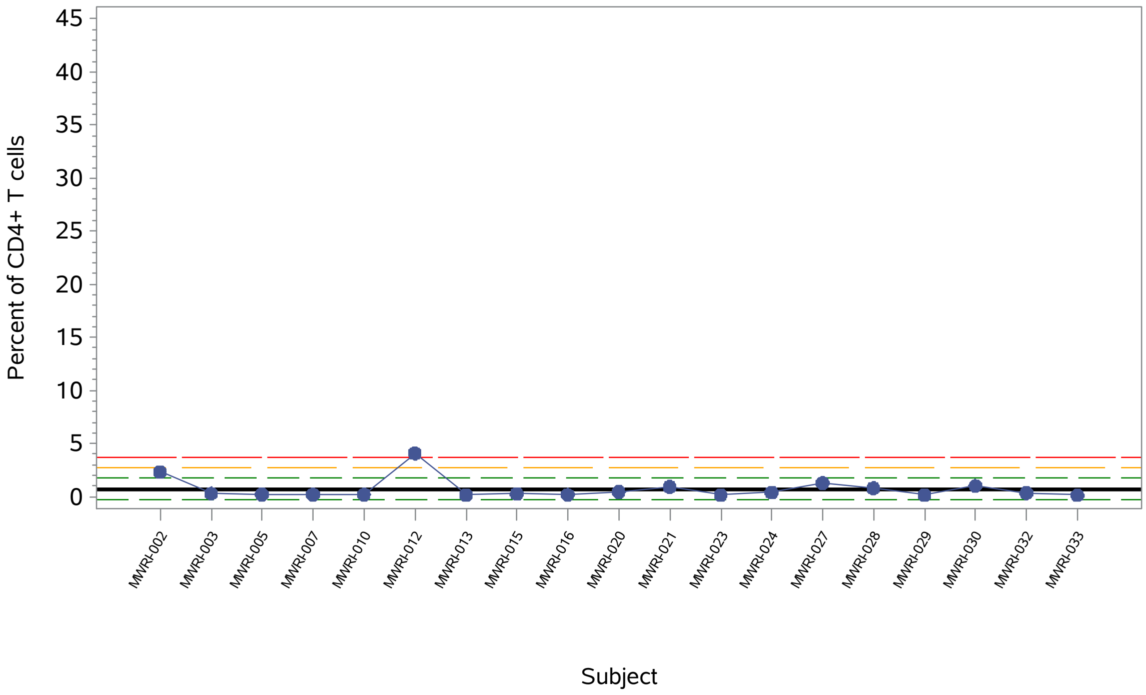

UCLA Memory Panel: CD4+CD27-CD45RA+

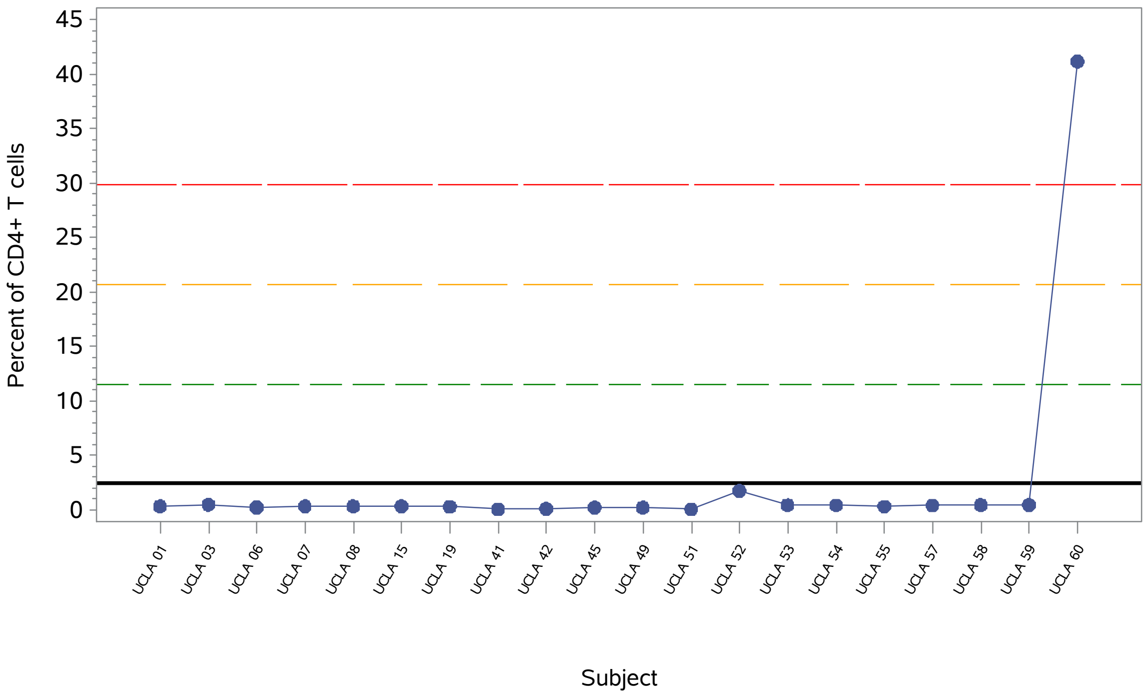

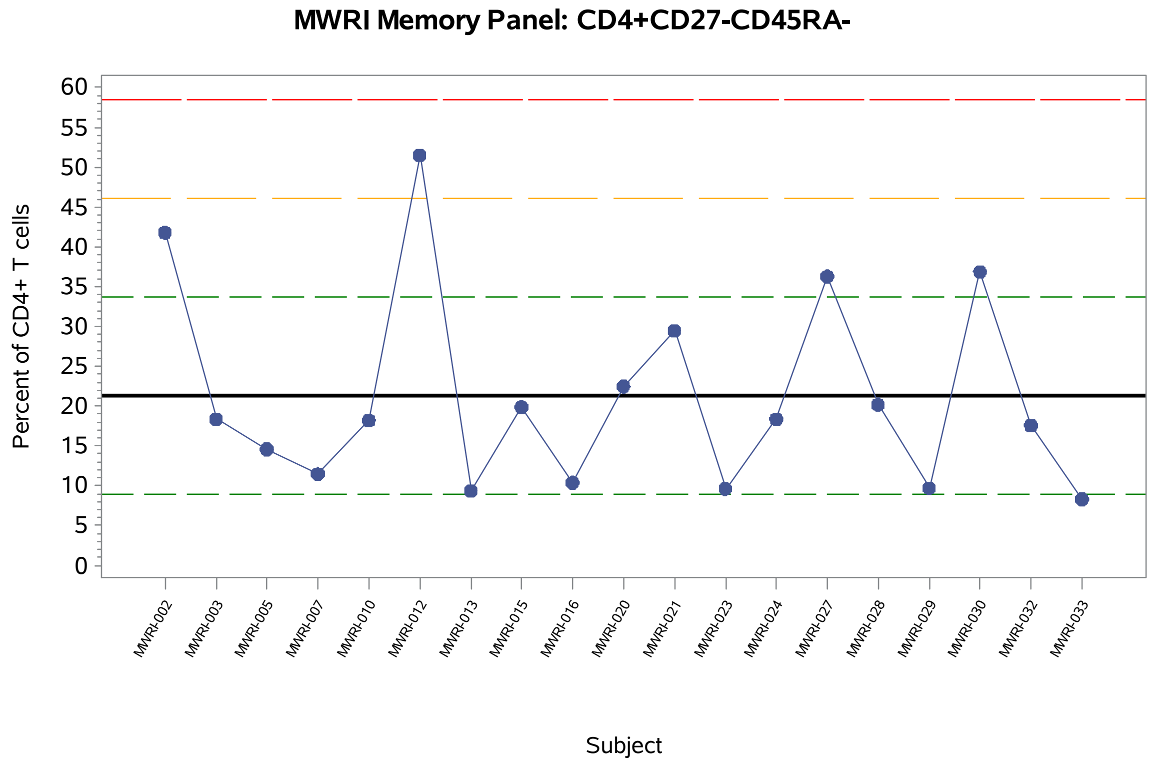
MWRI Memory Panel: CD4+CD27-CD45RA-
Percent of CD4+ T cells
60
55
50
45
40
35
30
25
20
15
10
5
0
MWRI-002
MWRI-003
MWRI-005
MWRI-007
MWRI-010
MWRI-012
MWRI-013
MWRI-015
MWRI-016
MWRI-020
MWRI-021
MWRI-023
MWRI-024
MWRI-027
MWRI-028
MWRI-029
MWRI-030
MWRI-032
MWRI-033
Subject

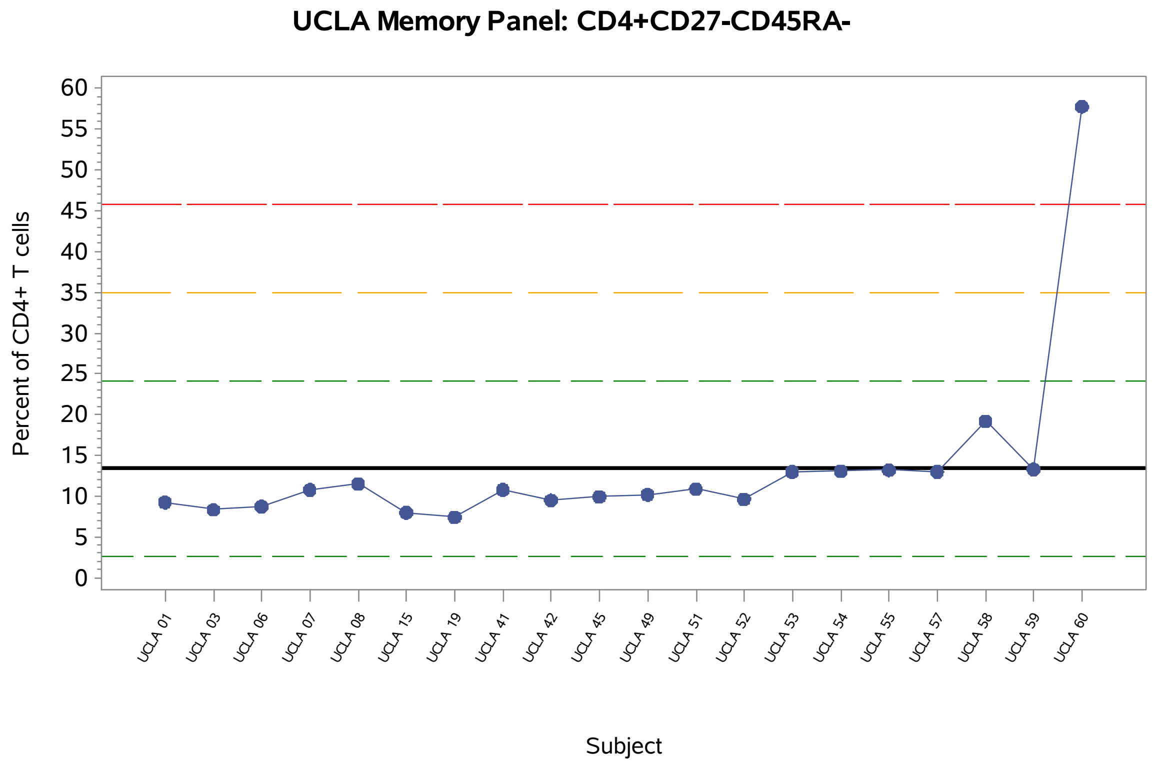
UCLA Memory Panel: CD4+CD27-CD45RA-
Percent of CD4+ T cells
60
55
50
45
40
35
30
25
20
15
10
5
0
UCLA 01
UCLA 03
UCLA 06
UCLA 07
UCLA 08
UCLA 15
UCLA 19
UCLA 41
UCLA 42
UCLA 45
UCLA 49
UCLA 51
UCLA 52
UCLA 53
UCLA 54
UCLA 55
UCLA 57
UCLA 58
UCLA 59
UCLA 60
Subject

MWRI Memory Panel: CD8+

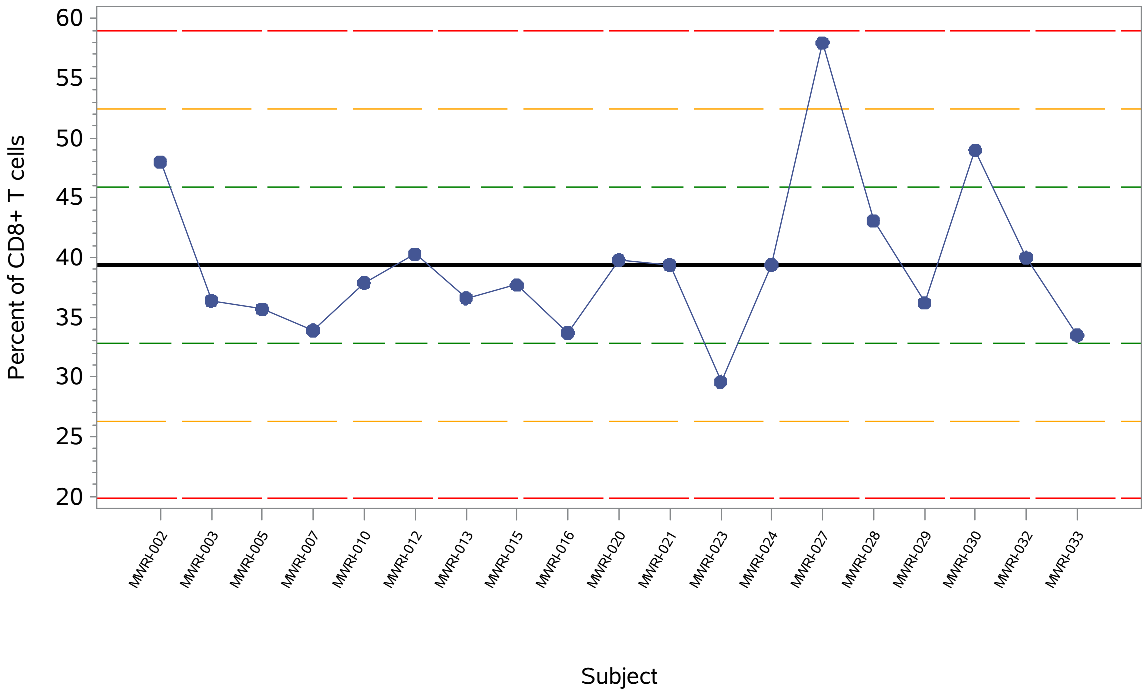

UCLA Memory Panel: CD8+

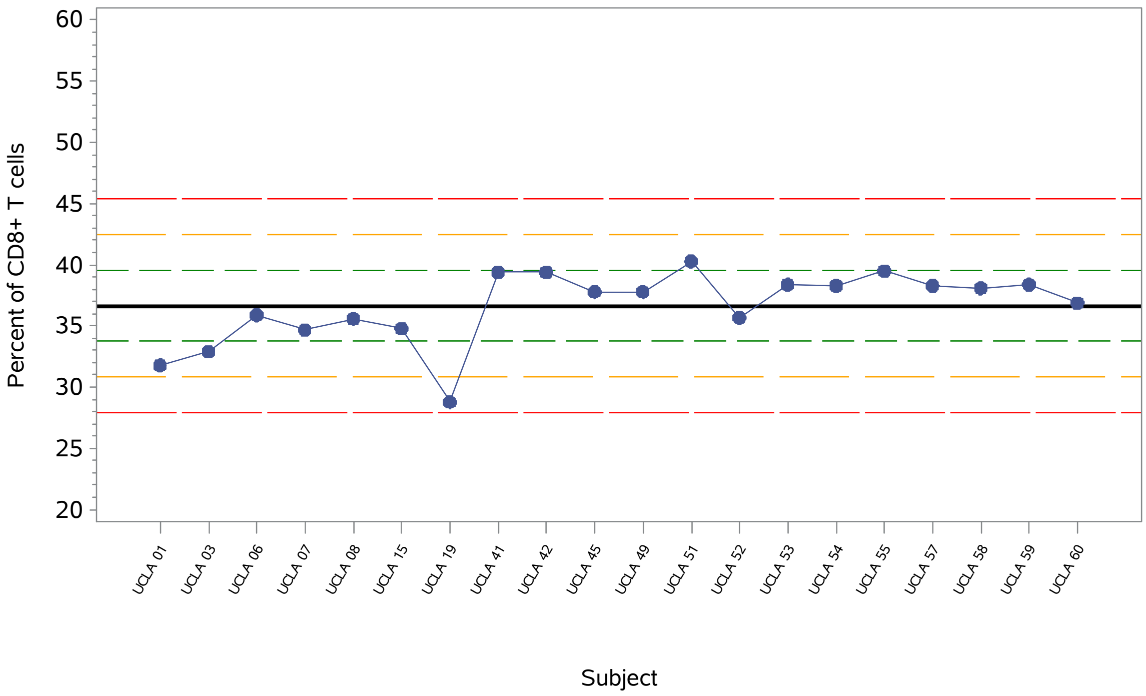

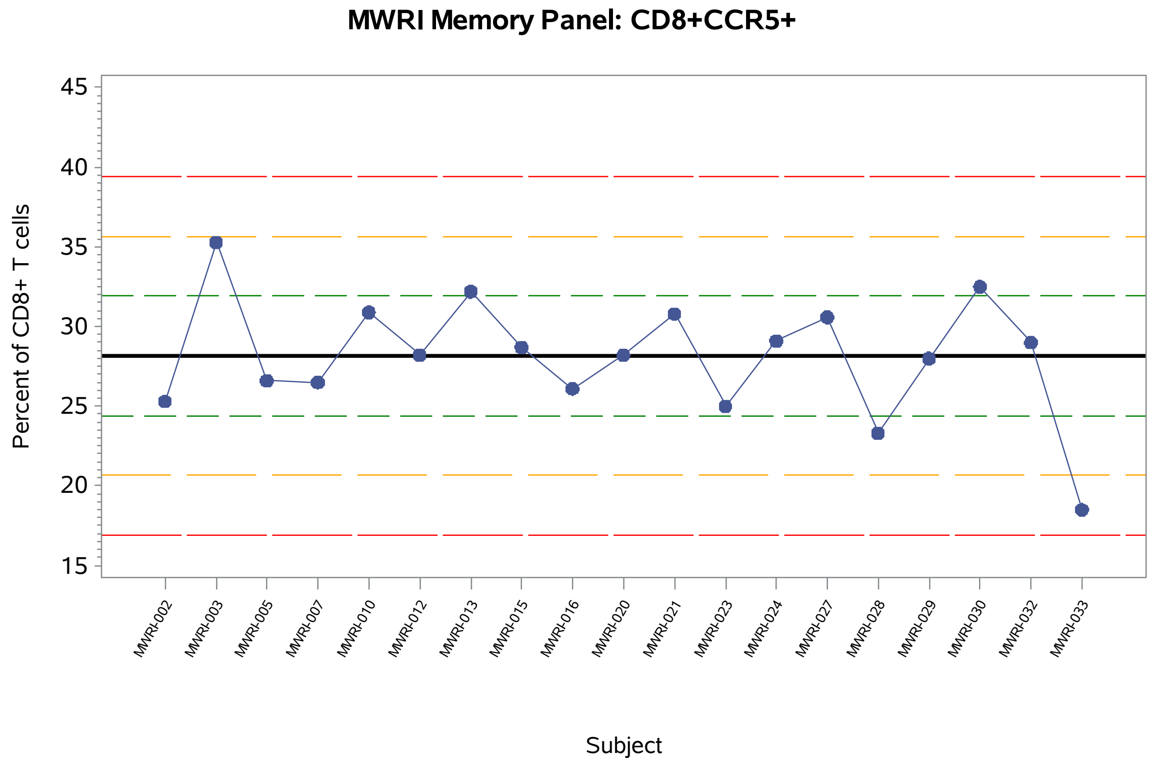
MWRI Memory Panel: CD8+CCR5+
Percent of CD8+ T cells
45
40
35
30
25
20
15
MWRI-002
MWRI-003
MWRI-005
MWRI-007
MWRI-010
MWRI-012
MWRI-013
MWRI-015
MWRI-016
MWRI-020
MWRI-021
MWRI-023
MWRI-024
MWRI-027
MWRI-028
MWRI-029
MWRI-030
MWRI-032
MWRI-033
Subject

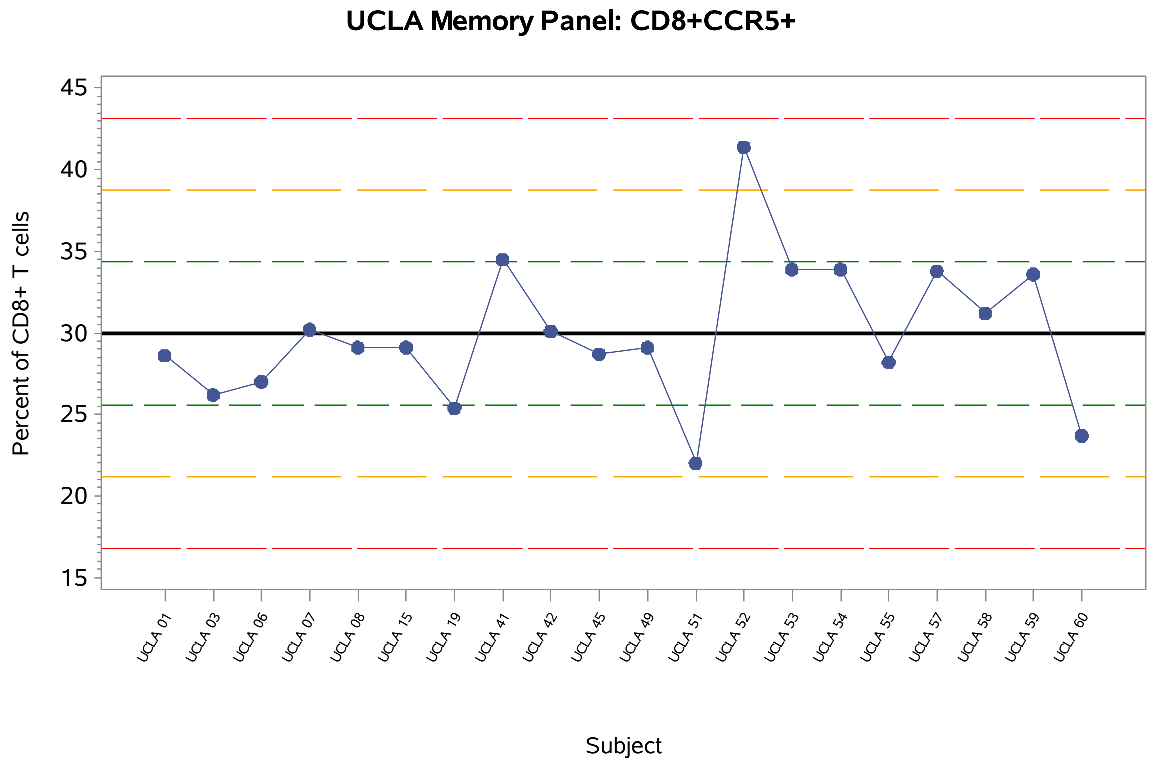
UCLA Memory Panel: CD8+CCR5+
Percent of CD8+ T cells
45
40
35
30
25
20
15
UCLA 01
UCLA 03
UCLA 06
UCLA 07
UCLA 08
UCLA 15
UCLA 19
UCLA 41
UCLA 42
UCLA 45
UCLA 49
UCLA 51
UCLA 52
UCLA 53
UCLA 54
UCLA 55
UCLA 57
UCLA 58
UCLA 59
UCLA 60
Subject

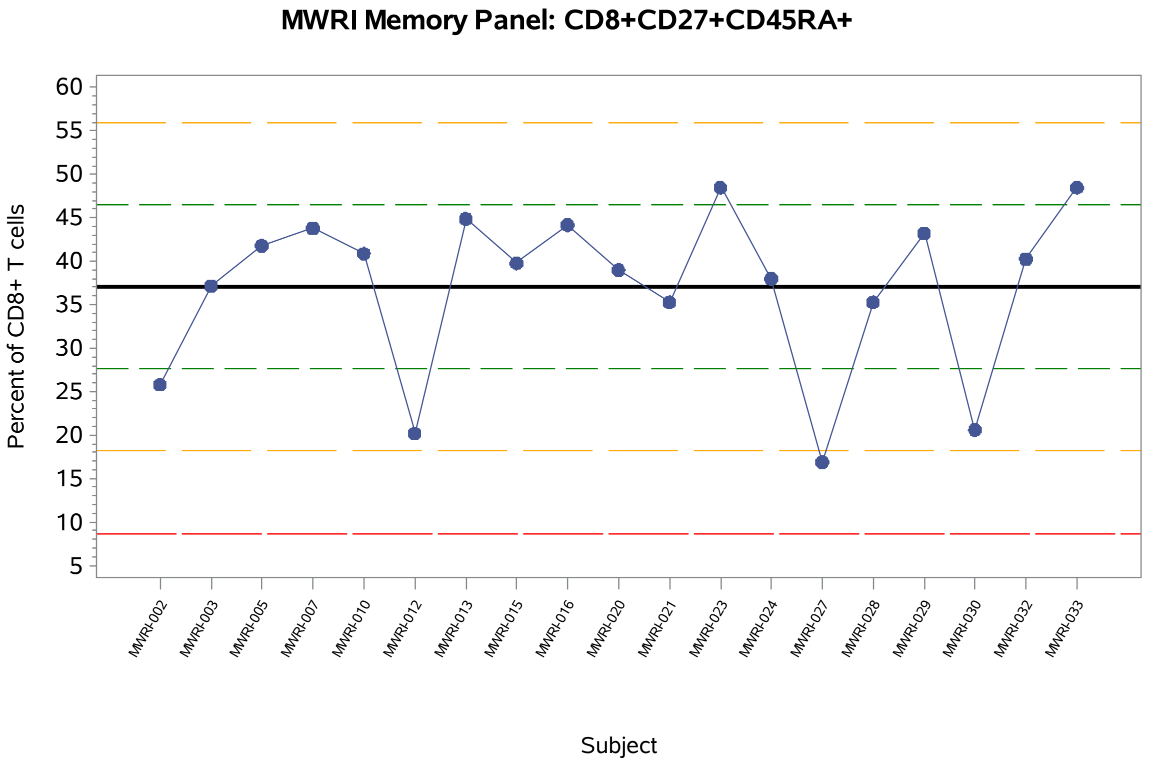
MWRI Memory Panel: CD8+CD27+CD45RA+
Percent of CD8+ T cells
60
55
50
45
40
35
30
25
20
15
10
5
MWRI-002
MWRI-003
MWRI-005
MWRI-007
MWRI-010
MWRI-012
MWRI-013
MWRI-015
MWRI-016
MWRI-020
MWRI-021
MWRI-023
MWRI-024
MWRI-027
MWRI-028
MWRI-029
MWRI-030
MWRI-032
MWRI-033
Subject

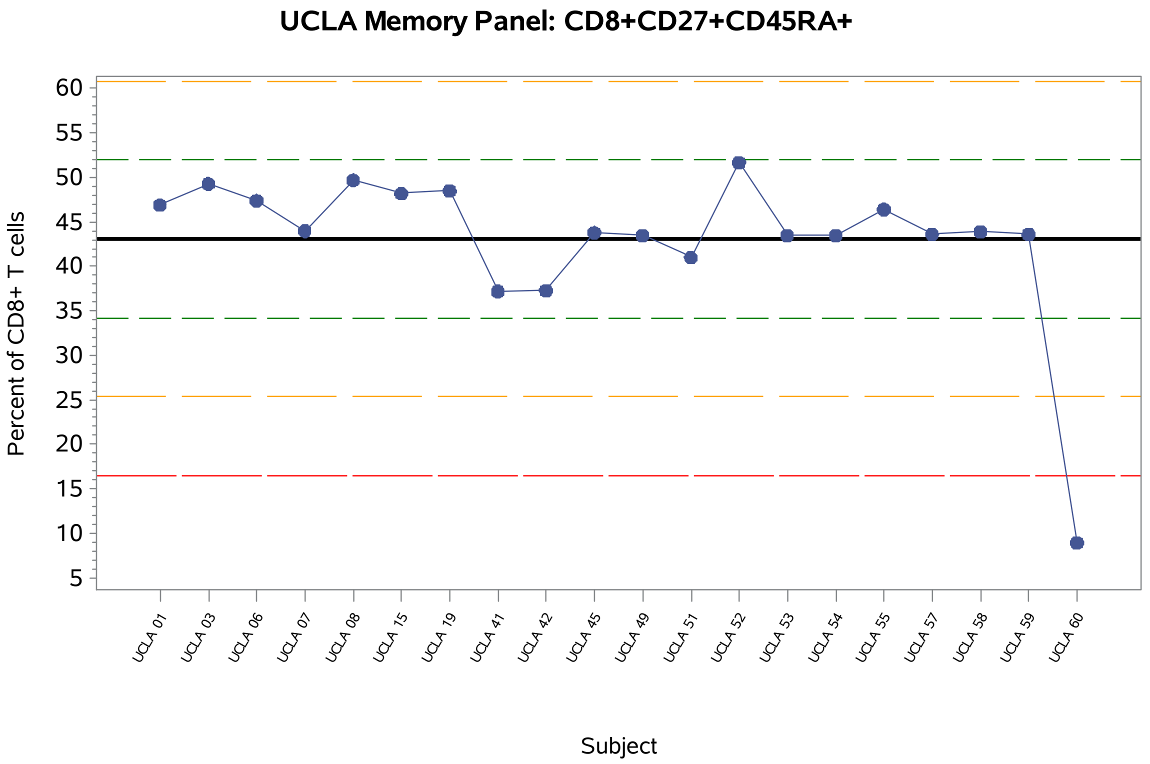
UCLA Memory Panel: CD8+CD27+CD45RA+
Percent of CD8+ T cells
60
55
50
45
40
35
30
25
20
15
10
5
UCLA 01
UCLA 03
UCLA 06
UCLA 07
UCLA 08
UCLA 15
UCLA 19
UCLA 41
UCLA 42
UCLA 45
UCLA 49
UCLA 51
UCLA 52
UCLA 53
UCLA 54
UCLA 55
UCLA 57
UCLA 58
UCLA 59
UCLA 60
Subject

MWRI Memory Panel: CD8+CD27+CD45RA-

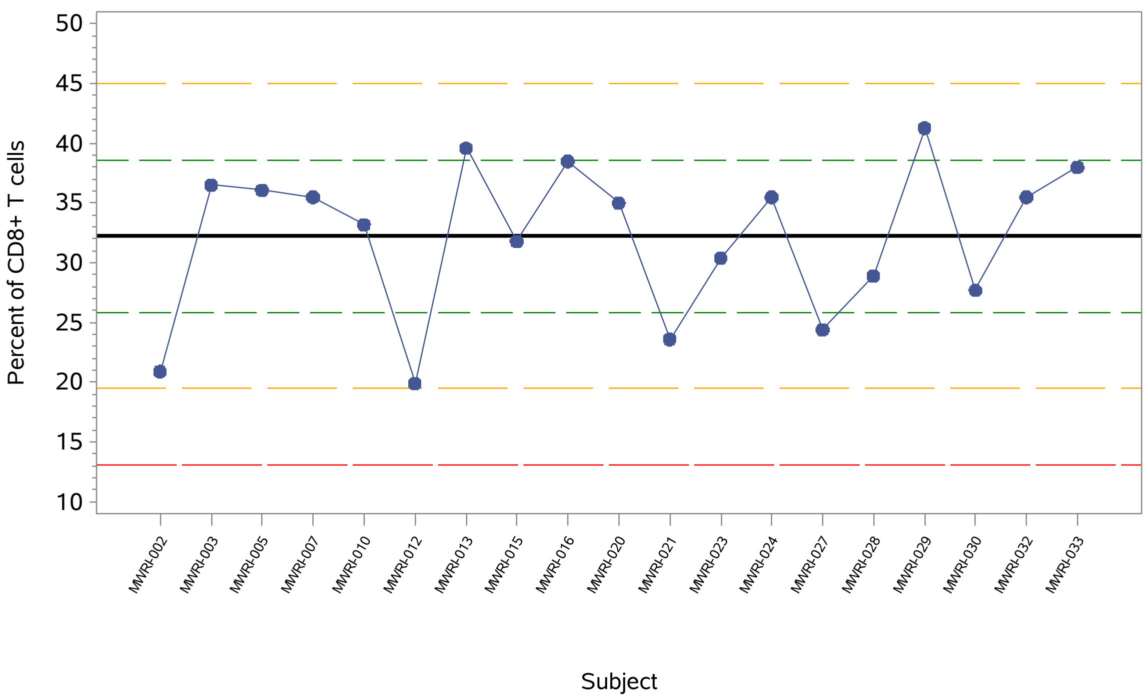

UCLA Memory Panel: CD8+CD27+CD45RA-

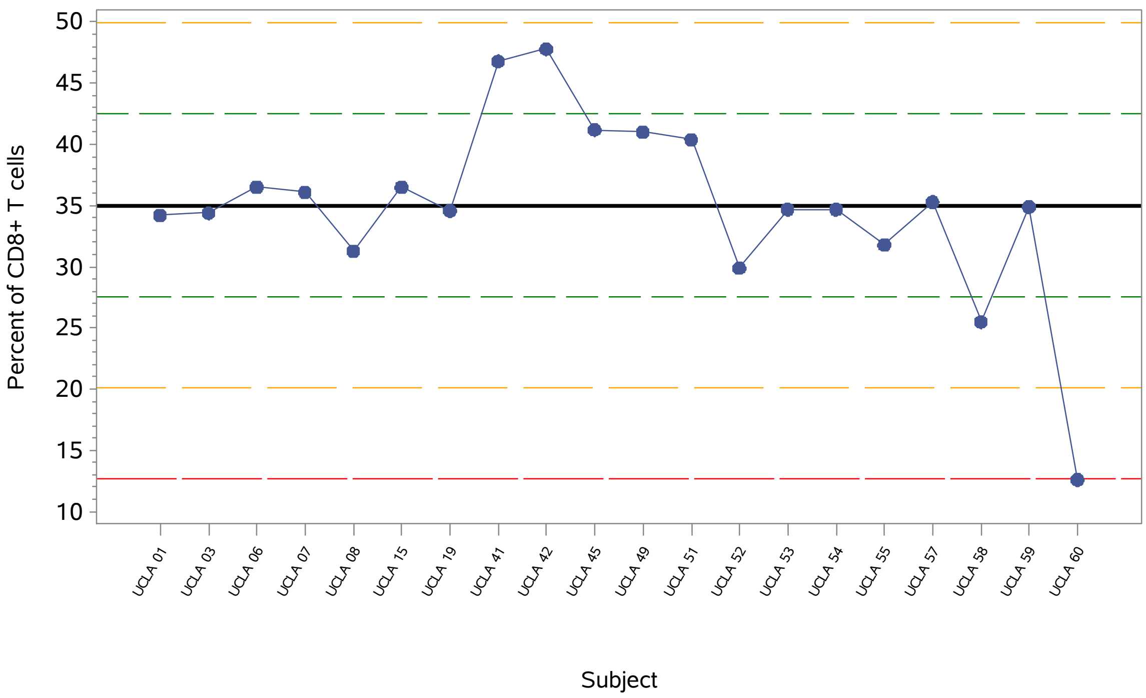

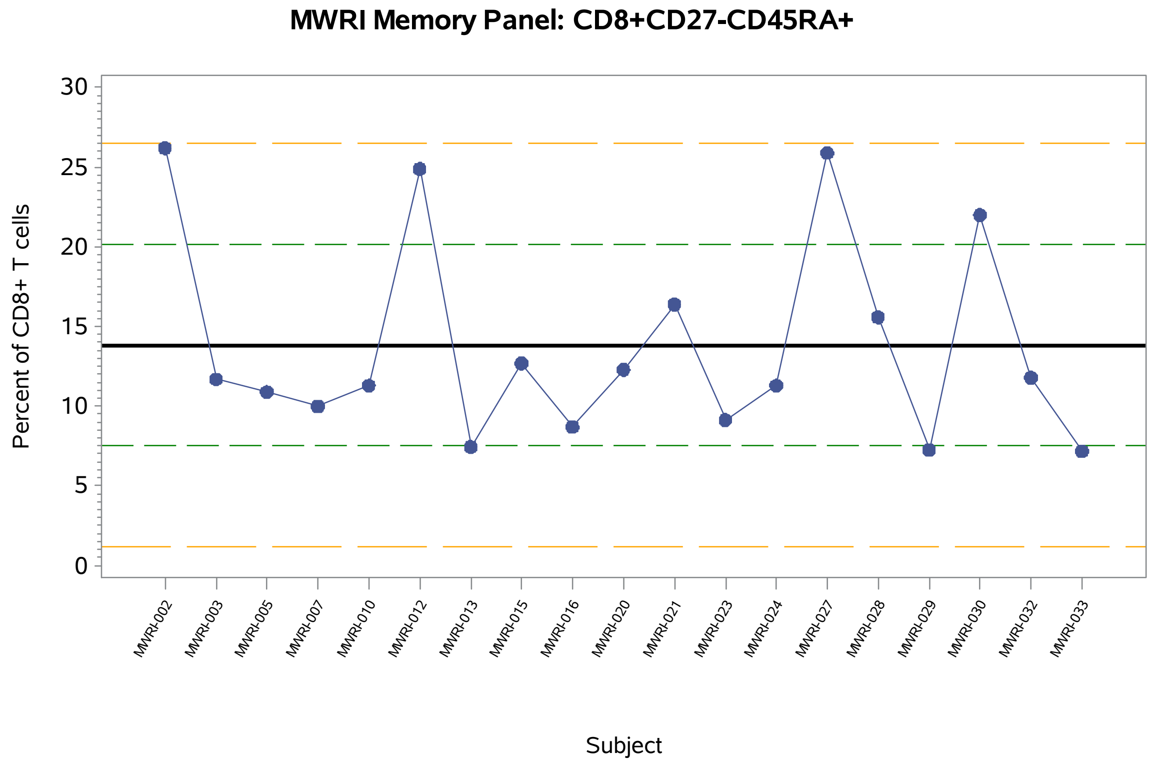
MWRI Memory Panel: CD8+CD27-CD45RA+
Percent of CD8+ T cells
30
25
20
15
10
5
0
MWRI-002
MWRI-003
MWRI-005
MWRI-007
MWRI-010
MWRI-012
MWRI-013
MWRI-015
MWRI-016
MWRI-020
MWRI-021
MWRI-023
MWRI-024
MWRI-027
MWRI-028
MWRI-029
MWRI-030
MWRI-032
MWRI-033
Subject

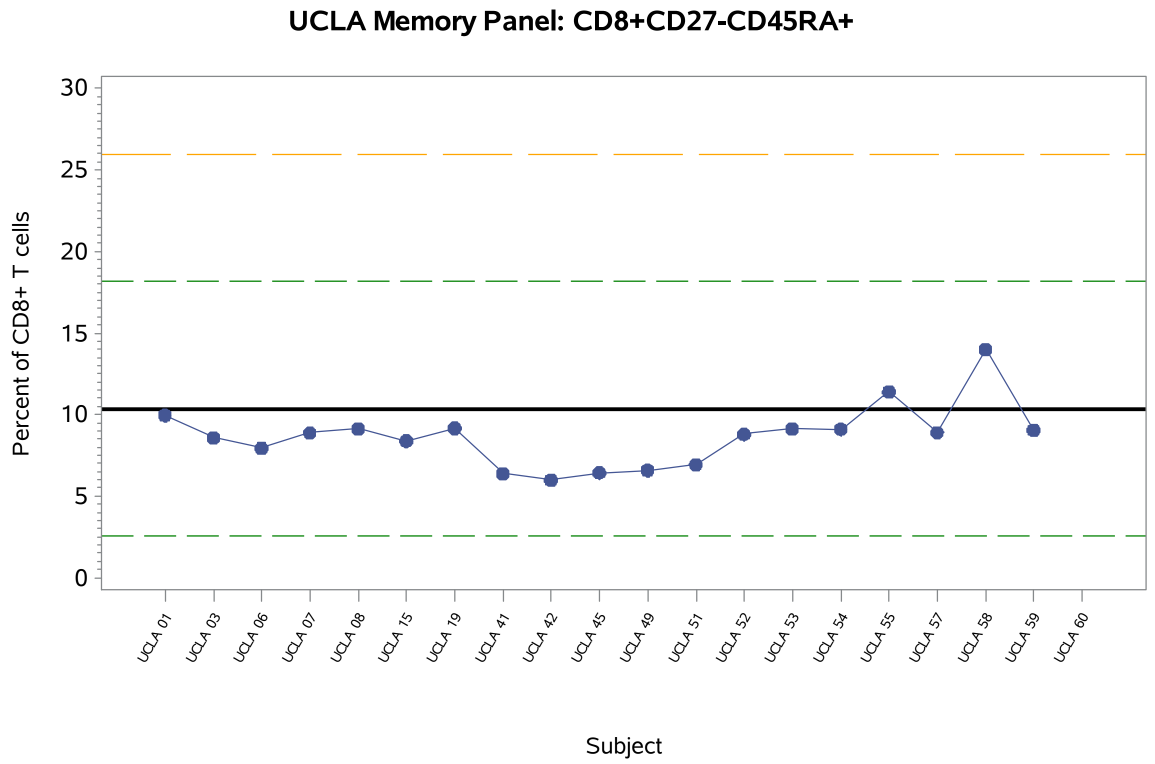
UCLA Memory Panel: CD8+CD27-CD45RA+
Percent of CD8+ T cells
30
25
20
15
10
5
0
UCLA 01
UCLA 03
UCLA 06
UCLA 07
UCLA 08
UCLA 15
UCLA 19
UCLA 41
UCLA 42
UCLA 45
UCLA 49
UCLA 51
UCLA 52
UCLA 53
UCLA 54
UCLA 55
UCLA 57
UCLA 58
UCLA 59
UCLA 60
Subject

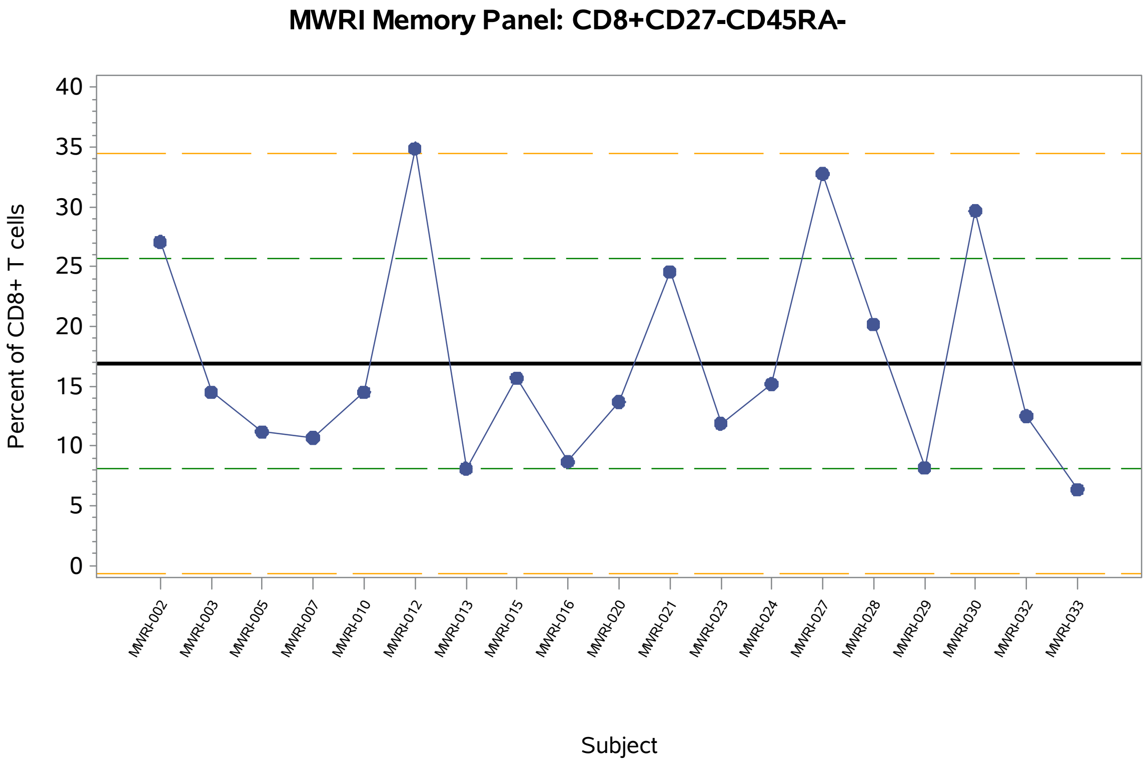

MWRI Memory Panel: CD8+CD27-CD45RA-
Percent of CD8+ T cells
40
35
30
25
20
15
10
5
0
MWRI-002
MWRI-003
MWRI-005
MWRI-007
MWRI-010
MWRI-012
MWRI-013
MWRI-015
MWRI-016
MWRI-020
MWRI-021
MWRI-023
MWRI-024
MWRI-027
MWRI-028
MWRI-029
MWRI-030
MWRI-032
MWRI-033
Subject

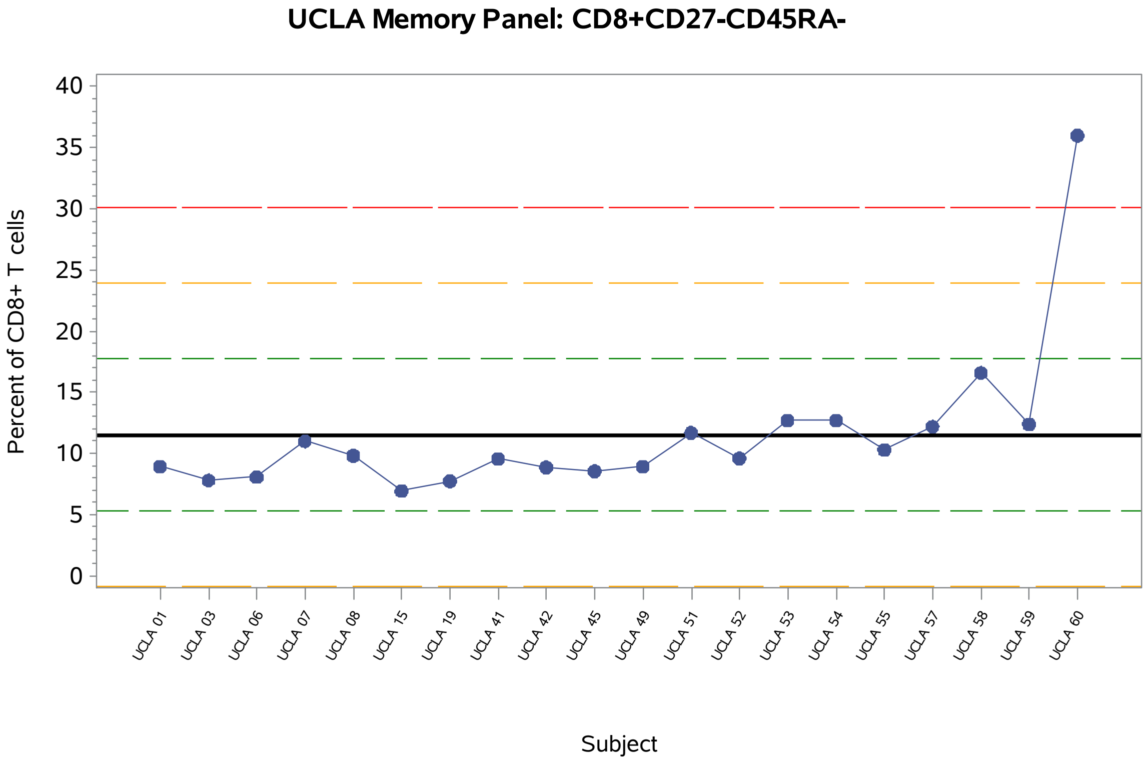

UCLA Memory Panel: CD8+CD27-CD45RA-
Percent of CD8+ T cells
40
35
30
25
20
15
10
5
0
UCLA 01
UCLA 03
UCLA 06
UCLA 07
UCLA 08
UCLA 15
UCLA 19
UCLA 41
UCLA 42
UCLA 45
UCLA 49
UCLA 51
UCLA 52
UCLA 53
UCLA 54
UCLA 55
UCLA 57
UCLA 58
UCLA 59
UCLA 60
Subject
