# Supplementary material for: Exploring the Feasibility of Multi-Site Flow Cytometric Processing of Gut Associated Lymphoid Tissue with Centralized Data Analysis for Multi-Site Clinical Trials
Source: PLoS One. 2015 May 26;10(5):e0126454. doi: 10.1371/journal.pone.0126454 (PMC4444258; doi:10.1371/journal.pone.0126454)
Supplement: S1 File — (PDF) [file pone.0126454.s003.pdf]

Mucosal Immunology Group

Consensus Protocol for Flow Cytometric Analysis of GALT T Cell Phenotype

Ian McGowan MD PhD
Peter Anton MD

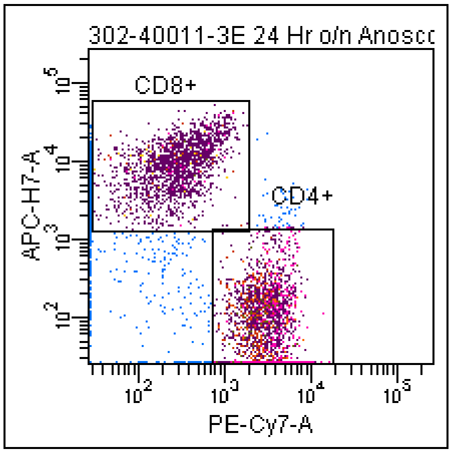

Table of Contents

1. Introduction ... 3
2. Study population ... 3
3. Tissue samples ... 3
4. Mononuclear Cell Isolation ... 3
   4.1. Mucosal Mononuclear Cells (MMC) for Flow Cytometric Analysis ... 3
   4.2. Peripheral Blood Mononuclear Cells (PBMC) for Flow Cytometric analysis ... 4
   4.3. Preparation of qPBMC for staining ... 4
      4.3.1. Thawing of qPBMC aliquots ... 4
5. In-vitro Stimulation (for ICS samples only) ... 5
6. Immunophenotyping ... 5
   6.1. Cell Viability Stain ... 5
   6.2. Cell Surface Antigen Staining ... 5
   6.3. Fixation and Permeabilization of cells ... 6
   6.4. Flow Cytometry Controls ... 6
      6.4.1. Flow Cytometer Compensation ... 7
      6.4.2. Viability Compensation: ... 7
      6.4.3. FMO Controls ... 7
      6.4.4. Biological Controls ... 8
7. LSR II and LSRFortessa Flow Cytometer Set-up ... 8
   7.1. Running the Cells and Compensation Beads: ... 9
   7.2. Gating Strategy for analysis: ... 9
8. Statistics ... 11
   8.1. Power Calculations ... 11
   8.2. Data Analysis ... 12

Mucosal Immunology Group Consensus Protocol for Flow Cytometric Analysis of GALT T Cell Phenotype

1. Introduction

Analysis of T cell populations derived from mucosal tissue samples is becoming a routine component of Phase 1 microbicide and vaccine studies [1-3]. Many of these studies are conducted at multiple sites and it is not known whether mucosal flow cytometric data collected at one clinical trial site can be compared with data generated at another site. Differences in clinical populations, tissue acquisition, and T cell isolation may prevent direct comparison between data sets. The problem is further exacerbated by use of different flow cytometer platforms and/or gating strategies.

The purpose of this pilot study is to develop a standardized approach to the collection of intestinal tissue, isolation of gut associated lymphoid tissue (GALT) mononuclear cells, and characterization of T cell phenotype. The two participating sites (the McGowan lab at the University of Pittsburgh School of Medicine and the Anton lab at the David Geffen School of Medicine at UCLA) will agree to use this approach to generate GALT T cell data even though the approach outlined in this protocol may differ from the experimental approach currently in use at their respective laboratories.

2. Study population

Each site will enroll up to 30 HIV, HSV-1/2, negative male participants without evidence of syphilis or anorectal GC/CT into the study. An interim analysis will be conducted after 15 participants have been enrolled at each site to determine whether sufficient data have been collected to demonstrate significant differences between the two sites.

3. Tissue samples

Fifteen endoscopic rectal biopsies will be collected at 10-15 cm from the anal margin. Ten ml of whole blood will also be collected from each participant. The study will therefore consist of the following populations: mucosal mononuclear cells (MMCs) isolated from rectal biopsies, study participant peripheral blood mononuclear cells (PBMC) isolated from whole blood and a standard biological control PBMC obtained from the HTVN laboratory in Seattle (qPBMC*).* Isolated mononuclear cells, whether MMC, PBMC, or qPBMC, will be referred to as “cells” in this protocol unless there is a need to identify a specific type of cell.

4. Mononuclear Cell Isolation

4.1. Mucosal Mononuclear Cells (MMC) for Flow Cytometric Analysis

MMC will be isolated from fifteen intestinal biopsies. Enzymatic digestion with a solution of Collagenase II and DNase 1 (hereafter referred to as “digest solution”) will be used to isolate cells from the intestinal biopsies. Collagenase II (50 mg, Sigma, #C-1764) and DNase 1 (2,000 U/ml, New England BioLabs, #M0303S) will be prepared fresh in RPMI 1640 medium containing L-glutamine and HEPES (Invitrogen, #22400-105) to a final concentration of 0.5 mg/ml and 0.83 U/ml, respectively. The RPMI is supplemented with 7.5% fetal bovine serum and 1% Antibiotic/Antimycotic (Invitrogen, #15240-104).

Biopsies will be scissor-minced 12 x 75 Falcon tubes (BD Falcon, #352058) in approximately 1 ml of digest solution, and then transferred to 50ml Corning plug-seal tubes containing 20 ml

fresh digest solution. Corning plug-seal tubes will be used because the tight fitting caps prevent leakage during incubation. The tubes will be secured horizontally to an orbital shaker and incubated for 30 min at 37°C at 350 rpm. After 30 min, the digests are passed through 40 uM Falcon cell strainers (BD, #21008-952) to collect the undigested tissue. The strainers fit inside the mouth of 50 ml Falcon tubes (BD, #21008-951) which are used to collect the dissociated cells. The cells are pelleted by centrifugation for 5 min at 2000 rpm (800 x g) and the supernatant fluid decanted. The cells are resuspended in Dulbecco's PBS supplemented with 2mM EDTA (D-PBS, Invitrogen, #14190-250) and kept on ice.

To retrieve additional cells, the strainer is held over a small Petri dish (VWR #82050-570) and a 1ml transfer pipette (Fisher Scientific #13-711-20) is used to rinse the remaining tissue pieces with several ml of digest solution. The tissue pieces are returned to the original 50 ml Corning conical tube to which 20 ml of fresh digest solution has been added. The tube is secured and incubated as described above for 30 min incubation at 37°C. The dissociated cells are again collected by passage through a cell strainer and centrifugation at 2000 rpm (800 x g) for 5 min.

The pooled cells from all digests will be collected by centrifugation at 2000 rpm (800 x g) for 5 min. Cell counts will be obtained using a hemocytometer and the concentrations recorded. The cells are resuspended in 500 ul Dulbecco's PBS supplemented with 2 mM EDTA (D-PBS, Invitrogen, #14190-250). Cell surface staining will be performed on 200ul of the cells; the remaining 300ul will be divided into a T-cell stimulation tube or an unstimulated control tube for intra-cellular cytokine staining (ICS), as described immediately below.

4.2. Peripheral Blood Mononuclear Cells (PBMC) for Flow Cytometric analysis

Whole blood will be diluted with an equal volume of D-PBS and placed in a 50 ml conical tube. The sample will be mixed by pipetting, underlayed carefully with 15 ml of Histopaque® 1077 (Sigma-Aldrich #10771-100ml) and then centrifuged for 1600 rpm (515 x g) for 30 minutes. The lymphocyte layer (interface) will be removed carefully with a Pasteur pipette while taking care not to disturb the layers and while avoiding the Histopaque® and supernatant layers as much as possible. The lymphocytes are then placed in a clean, sterile 50ml conical tube and washed with 50 ml of D-PBS. The cells are pelleted by centrifugation for 5 min at 2000 rpm (800 x g) and washed twice with 50 ml D-PBS. The cell pellet is resuspended in approximately 5 ml D-PBS and a 20 ul aliquot is reserved for obtaining cell counts using a hemocytomer.

4.3. Preparation of qPBMC for staining

The HVTN laboratory will supply each site with 45 frozen PBMC aliquots each containing 25 x 10^6 PBMC obtained from a single donor. qPBMC will be included as a quality control with each participant MMC and PBMC flow cytometric analysis. Flow data will be generated for the MMC, PBMC, and qPBMC. Hopefully, minimal variability will be seen in the qPBMC phenotypic data as the study proceeds.

4.3.1. Thawing of qPBMC aliquots

The vial of frozen qPBMCs will be immediately placed in a 37°C water bath after removal from the -80°C freezer. The vial will be held on the surface of the water and gently "flicked" during thawing. When a small amount of ice remains, the vial will be transferred to a bio-safety cabinet. The exterior of the vial is wiped with an alcohol swab prior to opening. About 1 ml of warmed cRPMI medium is then added in a slow, drop wise manner to each vial of thawed cells. The vial of cells is then transferred to 50 ml conical tubes containing 8 ml of cRPMI medium. The cells are centrifuged at 1600 rpm (515 x g) for 10 minutes. The supernatant fluid will be decanted and the remaining volume will be resuspended using a 200 ul volume sterile pipette. 10 ml of

cRPMI will be added to the tube which is centrifuged as described above. After decanting the supernatant fluid, the volume of remaining medium and cells is adjusted to 1 ml. Cell counts are obtained using a hemocytometer.

5. In-vitro Stimulation (for ICS samples only)

The cells destined for ICS (300ul of the total MMC prep) are centrifuged at 2000 rpm (800g) for 5 minutes prior to being resuspended in 1000ul of culture medium (RPMI 1640 with 10% FBS and antibiotic/antimycotic. 500ul is added to each of two wells of a 24-well tissue culture plate (BD Biosciences, #353047). To one well, PMA (Sigma, #P8139) is added at a final concentration of 10ug/ml, ionomycin (Sigma, #I0634) at a final concentration of 50ug/ml, and 0.5ul Golgi-Plug™ (BD Biosciences, #555028). Incubate the cells at 37°C in a humidified tissue culture incubator with 5% CO_2 for 4 hours. Harvest the stimulated (Tube 3) and non-stimulated (Tube 4) cells into 4ml Falcon round-bottom tubes (BD Biosciences #352058) and wash 2 x with D-PBS supplemented with 2mM EDTA. Spin for 7 minutes at 2000 rpm, (800 x g). Resuspend the cells from each tube in 100ul of D-PBS with 2 mM EDTA (referred to as wash solution) and proceed with the cell viability stain followed by cell surface staining protocol for CD45.

6. Immunophenotyping

Immunophenotyping by flow cytometry is to be performed by pre-staining with a viability dye followed by staining with one of three different eight-color study panels. Panel 1 will be stained for cell-surface antigens that characterize a memory phenotype. Panel 2 will be stained for surface antigens indicative of an activation phenotype. Panel 3 (Tubes 3 and 4, stimulated vs. non-stimulated cells, respectively) will be stained for a combination of cell surface and intracellular antigens to assess the presence of selected cytokines. Six Fluorescence Minus One (FMO) controls (i.e. a series of tubes where cells are stained with all mAb in a panel *except* the one that stains the population of interest) will be included to define the negative gates for those populations in Panels 1 and 2. Tube 4 (non-stimulated cells) will serve as the control to set the negative gate for Panel 3.

6.1. Cell Viability Stain

Cell viability will be assessed using the LIVE/DEAD® Fixable Dead Cell Stain Kit (Invitrogen, #L34957). The aqua fluorescent dye is reactive with cellular amines. In dead cells with compromised cell membranes, the dye reacts with the free amines in the interior of the cell, resulting in a 50-fold greater fluorescence in dead cells compared to live cells. One vial of reactive dye (Component A) is removed from the freezer and brought to room temperature prior to removal of the cap. A 50ul aliquot of DMSO (supplied in kit) is added and the vial is mixed well to dissolve the dye. The Aqua dye is placed into single use, 10ul aliquots and stored desiccated in the freezer at -20°C. For each experiment, an aliquot(s) of Aqua dye is removed from the -20°C freezer and placed in the dark to thaw at room temperature. The vial is vortexed and the appropriate volume (as determined by lot titration) of dye is added to each cell sample of approximately 200ul (1-2 million cells). The cells will be incubated with the dye in the dark at room temperature for 15 minutes.

6.2. Cell Surface Antigen Staining

While cells are incubating with the viability stain, a set of four tubes will be prepared for each sample. The tubes are set up with the following components listed in the Table 1 below. Exact mAb volumes will be determined by performing titration experiments on cells at each site laboratory prior to beginning the study. Reagents will be purchased in bulk so that the same lots are used for experiments at both sites. The reagents are vortexed prior to pipetting reagent into

the tubes. The following components (CD45, CD3, CD4 and CD8 mAb) are prepared and added as a cocktail while the other reagents are added individually.

Table 1: T cell memory and activation monoclonal panels

**Tube 1: T Cell Memory Tube**		**Tube 2: T Cell Activation Tube**	
CD45 PerCP	BD# 340665	CD45 PerCP	BD# 340665
CD3 Pac Blue	BD# 558117	CD3 Pac Blue	BD# 558117
CD4 PE-Cy7	BD # 557852	CD4 PE-Cy7	BD # 557852
CD8 APC-H7	BD# 557834	CD8 APC-H7	BD# 557834
CD45RA FITC	BD# 347723	HLA-DR FITC	BD # 347363
CCR5 PE	BD# 555993	CD38 PE	BD # 342371
CD27 APC	eBioscience#17-0279	CD69 APC	BD # 340560

After staining for viability, 100ul aliquot is added to the T Cell Memory Stain Tube. Similarly, a 100 ul volume of cells is added to the T Cell Activation Stain tube. For Tube 3 (ICS: stimulated MMC) and Tube 4 (ICS: unstimulated MMC), the dedicated cells (either PMA/ionomycin stimulated or unstimulated, 150 uL each) are first surface-stained for CD45. These samples are incubated at room temperature in the dark with the appropriate amount of CD45 mAb for 20 minutes. The cell pellets are washed twice with 1 ml wash buffer (D-PBS/2 mM EDTA) with vortexing. The cell pellets are obtained by centrifugation (800 x g for 7 minutes) and decanting the supernatant. Panels 1 & 2 with appropriate FMO controls are resuspended in 250ul of 1% formaldehyde solution (made from 20% stock, Electron Microscopy Sciences #15713-S) and stored at 4^0C in the dark for next-day analysis on the BD LSRII™ (UCLA) or the LSRFortessa™ (Pittsburgh) flow cytometers. Tubes 3 & 4 are processed as follows:

6.3. Fixation and Permeabilization of cells

Thoroughly resuspend the cells in 250ul of BD Cytofix/Cytoperm™ Fixation/Permeabilization Solution (BD Biosciences, # 554714) and incubate at 4°C for 20 minutes. Wash the cells twice in 1ml of BD Perm/Wash™ buffer (BD Biosciences, # 554714) After the final wash, resuspend the cell pellet thoroughly in 50ul of Perm/Wash™ buffer containing a cocktail of antibodies (Table 2) at optimal concentrations (previously determined) as follows:

Table 2: T cell intracellular cytokine stimulation monoclonal panel

**Monoclonal Antibody**	**Catalogue Number**
CD3 Pacific Blue	BD # 558117
CD4-PECy7	BD # 557852
CD8- APC-H7	BD # 557834
Anti- TNF–alpha FITC	BD # 340511
Anti-IL-2 PE	BD # 559334
Anti-Interferon-gamma APC	BD # 341117

Incubate for 30 minutes at 4°C in the dark. Wash cells twice with 1 mL Perm/Wash™ buffer as described above and resuspend in 200uL staining buffer (D-PBS/2% FBS) prior to flow cytometric analysis.

6.4. Flow Cytometry Controls

In parallel with the set up of investigational immunonophenotyping tubes, the following control tubes are required for each assay.

6.4.1. Flow Cytometer Compensation

Compensation will be set individually for each of the fluorochromes in the panels. Prepare one compensation set for each flow cytometry run, with appropriate volumes of each of the mAb conjugated fluorochromes listed below (Table 3). Each tube also receives one drop each of BD positive and BD negative compensation beads (Anti-Mouse Ig, κ/Negative Control (FBS) Compensation Particles Set, BD #552843). The set contains two populations of polystyrene microparticles which are used to optimize fluorescence compensation settings for multicolor flow cytometry. The positive Anti-Mouse Ig, κ particles bind any mouse κ light chain-bearing immunoglobulin while the negative particles have no binding capacity.

Table 3: Compensation sets for flow cytometry

**Fuorochrome**	**Mab**	**Fuorochrome**	**Mab**
PerCp	CD45 (20ul)	APC	CD69 (20 ul)
Pacific blue	CD3 (5 ul)	FITC	CD45RA (20 ul)
PECy7	CD4 (5 ul)	PE	CD38 (20 ul)
APCH7	CD8 (5 ul)		

6.4.2. Viability Compensation:

Compensation staining for the Aqua fluorescent dye is performed separately using the ArC™ Amine Reactive Compensation Bead Kit (Invitrogen Catalog #A10346). The ArC™ contains two types of specially modified polystyrene microspheres to allow compensation with the LIVE/DEAD® Fixable Dead Cell Stain Kit. The positive beads (Component A) will bind the amine-reactive dye while the ArC™ negative microspheres (Component B) have no reactivity. Gently vortex kit reagents for 30 seconds. Add one drop of ArC™ positive beads to the designated compensation tube and allow the beads to warm to room temperature for 5 minutes. Remove 3uL of Aqua dye, mix well and add to the beads which will be incubated in the dark at room temperature. After a 30-minute incubation, the beads will be washed with 3 ml of D-PBS and centrifuged for 5 minutes at 2000 rpm (500 x g). Resuspend the bead pellet in 0.5mL D-PBS and add one drop of ArC™ negative beads. The tubes will be mixed thoroughly before analysis by flow cytometry.

6.4.3. FMO Controls

Initially, PBMC will be used to establish cursor settings for those markers that are difficult to define as positive or negative. Once FMO's are developed for PBMC, they will be run on the PBMC and MMC samples from the first three participants to confirm their validity. If it is determined that the FMO gates are appropriate, the cells from the remaining participants will be stained for the full stain panels only. FMO panels are presented below (Table 4a, 4b & 4c)

Table 4a: Tube #1 FMO Controls-Memory Panel

	FMO **a**	FMO **b**	FMO **c**
CD45-PerCP	+	+	+
CD3-Pac Blue	+	+	+
CD4- PE-Cy7	+	+	+
CD8-APC-H7	+	+	+
CD45RA-FITC		+	+

CD27-APC	+		+
CCR5-PE	+	+	
AViD	+	+	+

Table 4b: Tube #2 FMO Controls-Activation Panel

	FMO **d**	FMO **e**	FMO **f**
CD45-PerCP	+	+	+
CD3-Pac Blue	+	+	+
CD4-PE-Cy7	+	+	+
CD8-APC-H7	+	+	+
HLA-DR-FITC		+	+
CD38-PE	+		+
CD69-APC	+	+	
AViD	+	+	+

Table 4c: Tube #3 FMO Controls-ICS Panel

	FMO **g**	FMO **h**	FMO **i**
CD45-PerCP	+	+	+
CD3-Pac Blue	+	+	+
CD4-PE-Cy7	+	+	+
CD8-APC-H7	+	+	+
Anti-TNFα-FITC		+	+
Anti-IL-2-PE	+		+
Anti-IFNγ-APC	+	+	
AViD	+	+	+

6.4.4. Biological Controls

A standard biological control (frozen PBMC from a single donor) will be supplied to each site by the HVTN Laboratory in Seattle (qPBMC). The HVTN laboratory will establish reference population frequencies for this sample. qPBMC will be stained alongside each new subject's sample (at each study site) and will serve as a quality control (QC) reference to aid in the validation of the data acquisition process and as a reference point to help characterize inter-subject variation.

7. LSR II and LSRFortessa Flow Cytometer Set-up

Cytometers at each site will be configured to be as identical as possible. Optimum Photomultiplier tube (PMT) settings for each flurochrome will be selected at one site using PBMCs stained with each flurochrome and target median fluorescent intensities (MFI) generated for each detector of interest using Cytometer Setup & Tracking (CST) beads (BD, San Jose, CA). Target MFI's will be duplicated at the alternate site using the same lots of beads. On an experimental basis, the target MFI's will be achieved by manual adjustment of the PMT voltages, to obtain the target medians in each channel. All acquired flow cytometric data will be analyzed by FlowJo software v9.0.1 (Tree Star, Inc., Ashland, OR).

7.1. Running the Cells and Compensation Beads:

Approximately 5,000 compensation beads per tube will be acquired. Approximately 20,000 CD8 positive events will be recorded per immunophenotyping tube.

7.2. Gating Strategy for analysis:

Common analysis templates will be developed for use at both study sites. The following hierarchical gating strategy to identify CD4 positive and CD8 positive T-cell subsets will be employed for all tubes:

i) CD45 gate (SSC-A vs CD45)
ii) Single cells (SSC-H vs SSC-W)
iii) CD3 Live (CD3 vs AViD)
iv) CD4 and CD8 T-cell gates

Example of gating strategy for MMC sample

i) **ii)** **iii)** **iv)**

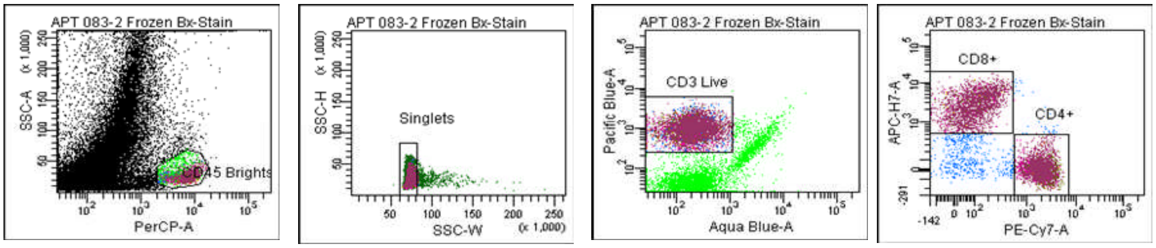

Tube 1 (Memory panel)

Plots for both CD4+ and CD8+T-cell subsets will be generated as above to display the following parameters shown below (cursor settings to be established using FMO controls for Tube 1).

CD45RA versus CD27 (PBMC sample)

CD4 Gate

CD45RA

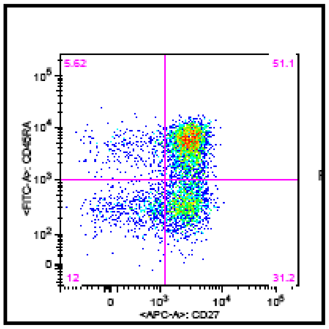

CD27

CD8 Gate

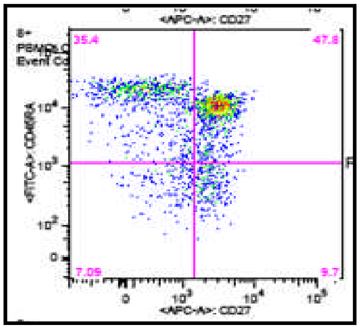

CD27

CD45RA versus CCR5 (PBMC sample)

CD4 Gate

CD8 Gate

CD45RA

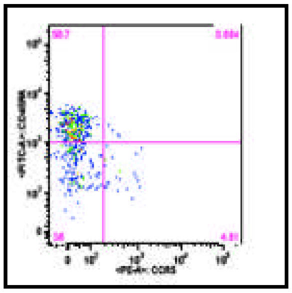

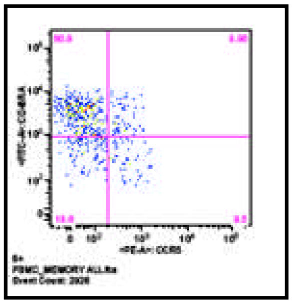

CCR5

CCR5

Tube 2 (Activation panel)

Plots for CD4+ and CD8+ T-cell subsets will be generated, as above, to display the following parameters (shown below) (cursor settings to be established using FMO controls for Tube 2):

CD38 versus HLA-DR (PBMC sample)

CD4 Gate

CD8 Gate

HLA-DR

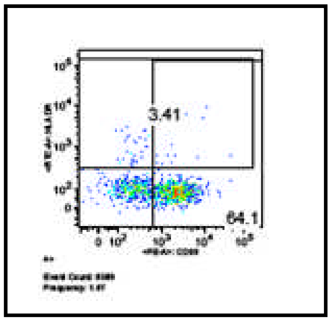

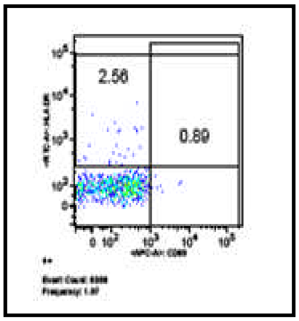

CD38

CD38

CD69 versus HLA-DR (PBMC sample)

CD4 Gate

CD8 Gate

HLA-DR

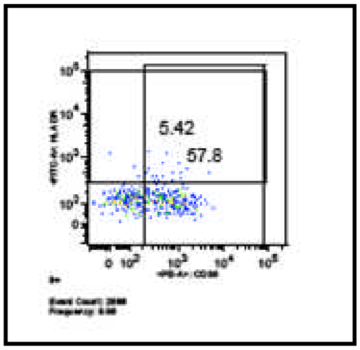

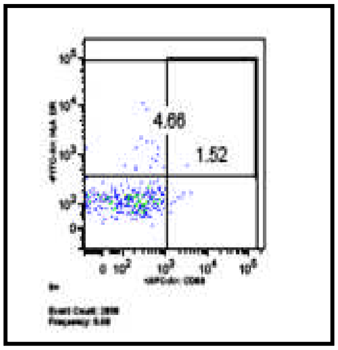

CD69

CD69

Tube 3 (ICS panel)

Plots for CD4+ and CD8+ T-cell subsets will be generated, as above, to display the following parameters shown below:

<u>Cursors to be set using unstimulated cells (Tube 4)</u>

i) IL-2-PE versus INF-γ-APC **(PBMC sample)**

ii) TNF-a FITC versus INF-γ-APC **(PBMC sample)**

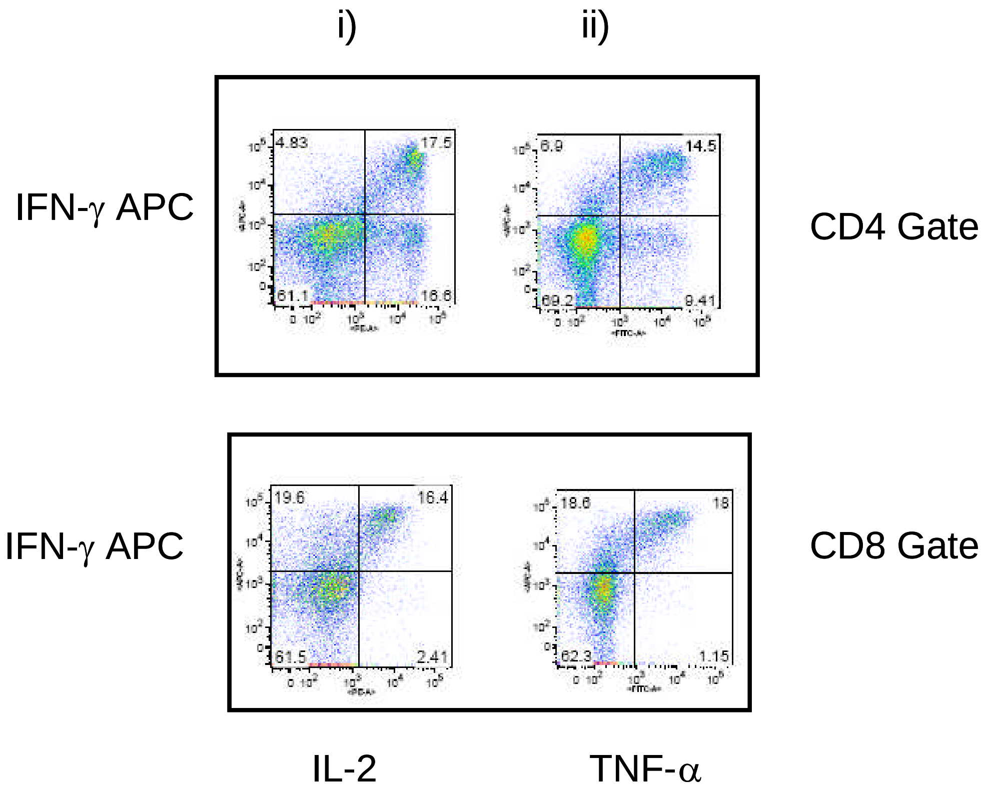

8. Statistics

8.1. Power Calculations

The Statistical Core at the University of Pittsburgh has conducted power calculations to determine the feasibility of determining site specific differences is T cell phenotype with sample size (at each site) varying between 10-30 participants. The power calculations were modeled using the variability seen in baseline GALT T cell phenotype data obtained from 25 participants in an ongoing Phase 1 rectal microbicide study (MTN-007). Prospective power calculations were performed using SAS/STAT 9.2 PROC POWER, characterizing the power to detect ratios of site mean measurements equal to 1.1, 1.2, 1.3, and 1.5 from sample sizes of 10, 20, and 30 at each site. The power to detect differences between sites varied by T cell phenotype (Table 5) and

was predominantly a reflection of the variability seen across T cell phenotypic markers. For common T cell markers such as the proportion of CD45 positive cells co-expressing the CD3 receptor, the study had 83-99% power to detect 1.5 fold differences across sites.

Table 5: Power calculation for ability to determine fold difference in T cell phenotype across study sites

**Fold Difference in Phenotype**	**1.1**	**1.2**	**1.3**	**1.5**
**N (each site)**				
**% CD69 from CD4 / % CD69 from CD8**				
**10**	**0.18**	**0.50**	**0.80**	**0.99**
**20**	**0.32**	**0.81**	**0.98**	**>0.99**
**30**	**0.45**	**0.94**	**0.99**	**>0.99**
**% CD3 from CD45 / % CD4 from CD3 / % CD8 from CD3 / % CCR5 from CD8**				
**10**	**0.11**	**0.26**	**0.47**	**0.83**
**20**	**0.17**	**0.48**	**0.79**	**0.99**
**30**	**0.24**	**0.66**	**0.93**	**>0.99**
**% CCR5 from CD4**				
**10**	**0.08**	**0.17**	**0.30**	**0.61**
**20**	**0.12**	**0.31**	**0.56**	**0.90**
**30**	**0.16**	**0.44**	**0.74**	**0.98**

8.2. Data Analysis

Tabulated statistical data for study MMC specimens and qPBMC will be generated in FlowJo and exported into Excel spreadsheets at each site. Raw data files and statistical tables, using pre-defined templates, will be exported to the MIG portal for the purposes of data monitoring and compilation. Data will be analyzed at both sites and will also be analyzed by HVTN (Dr Steve De Rosa). Potential differences between the two data sets (Pittsburgh and UCLA) will be explored using the Mann-Whitney test for non parametric data. The following parameters (Table 6) will be compared between sites:

Table 6: Summary of T cell flow parameter endpoints

T cell phenotype	T cell activation	T cell ICS
CD3||CD45	HLA-DR||CD4	IL-2||CD4
CD4||CD3	CD38||CD4	TNF-α||CD4
CD8||CD3	CD38+HLA-DR+||CD4	INF-γ||CD4
CD45RA||CD4	CD69||CD4	IL-2+INF-γ+||CD4
CD45RA+CCR5+||CD4	CD69+HLA-DR+||CD4	TNF-α+INF-γ+||CD4
CD45RA+CD27+||CD4	HLA-DR||CD8	IL-2||CD8
CD45RA||CD8	CD38||CD8	TNF-α||CD8
CD45RA+CCR5+||CD8	CD38+HLA-DR+||CD8	INF-γ||CD8
CD45RA+CD27+||CD8	CD69||CD8	IL-2+INF-γ+||CD8
	CD69+HLA-DR+||CD8	TNF-α+INF-γ+||CD8

References

1. McGowan I, Elliott J, Cortina G, Tanner K, Siboliban C, Adler A*, et al.* Characterization of baseline intestinal mucosal indices of injury and inflammation in men for use in rectal microbicide trials (HIV Prevention Trials Network-056). *J Acquir Immune Defic Syndr* 2007; **46(4)**:417-425.

2. Anton P, Adler A, Khanukova E, Elliott J, Cumberland W, Zhou Y*, et al.* A Phase 1 rectal safety and acceptability study of UC781 microbicide gel [Abstract]. *16th Conference on Retroviruses and Opportunistic Infections Montreal, Canada* 2009.

3. Jamieson BD, Ibarrondo FJ, Wong JT, Hausner MA, Ng HL, Fuerst M*, et al.* Transience of vaccine-induced HIV-1-specific CTL and definition of vaccine "response". *Vaccine* 2006; **24(17)**:3426-3431.
