# Supplementary material for: Exploring the Feasibility of Multi-Site Flow Cytometric Processing of Gut Associated Lymphoid Tissue with Centralized Data Analysis for Multi-Site Clinical Trials
Source: PLoS One. 2015 May 26;10(5):e0126454. doi: 10.1371/journal.pone.0126454 (PMC4444258; doi:10.1371/journal.pone.0126454)
Supplement: S1 Table — (DOCX) [file pone.0126454.s004.docx]

**Supplemental Table 1** PBMC comparisons stratified by HSV-1 status

| Characteristic | UCLA  (N=18) | HSV-1  negative  (N=12) | HSV-1  positive  (N=6) | t-test  p-value  (Mean) | Wilcoxon  p-value  (Median) |
| --- | --- | --- | --- | --- | --- |
|  | Mean ± SD | | |  |  |
| Activation Panel (%) |  |  |  |  |  |
| CD4+ % of CD3+ cells | 62.8 ± 11.7 | 62.3 ± 12.7 | 63.8 ± 10.4 | 0.8092 | 0.7431 |
| CD38+DR+ % of CD4+ cells | 2.8 ± 2.7 | 2.1 ± 1.0 | 4.1 ± 4.3 | 0.2969 | 0.1598 |
| CD69+ % of CD4+ cells | 4.2 ± 6.0 | 3.5 ± 5.6 | 5.5 ± 7.3 | 0.5296 | 0.3736 |
| CD8+ % of CD3+ cells | 30.6 ± 9.5 | 30.6 ± 10.5 | 30.7 ± 7.9 | 0.9853 | 0.9253 |
| CD38+DR+ % of CD8+ cells | 7.6 ± 5.7 | 6.1 ± 4.1 | 10.8 ± 7.6 | 0.1035 | 0.1112 |
| CD69+ % of CD8+ cells | 7.7 ± 9.0 | 7.0 ± 8.6 | 9.0 ± 10.5 | 0.6695 | 0.8149 |
| Memory Panel (%) |  |  |  |  |  |
| CD4+ % of CD3+ cells | 64.2 ± 11.5 | 63.8 ± 12.4 | 64.9 ± 10.5 | 0.8611 | 1 |
| CCR5+ % of CD4+ cells | 12.6 ± 9.5 | 9.7 ± 6.3 | 18.4 ± 12.5 | 0.0632 | 0.1338 |
| CD27+CD45RA+% of CD4+ cells | 48.8 ± 17.2 | 48.9 ± 16.3 | 48.6 ± 20.3 | 0.9792 | 0.8406 |
| CD27+CD45RA- % of CD4+ cells | 39.0 ± 12.8 | 40.4 ± 11.3 | 36.5 ± 15.9 | 0.5672 | 0.3654 |
| CD27-CD45RA+ % of CD4+ cells | 1.4 ± 1.9 | 0.6 ± 1.4 | 2.8 ± 2.1 | 0.0204 | 0.0237 |
| CD27-CD45RA- % of CD4+ cells | 10.8 ± 6.3 | 10.1 ± 6.9 | 12.1 ± 5.4 | 0.5624 | 0.546 |
| CD8+ % of CD3+ cells | 28.8 ± 9.8 | 28.8 ± 11.0 | 28.7 ± 8.0 | 0.9755 | 0.9253 |
| CCR5+ % of CD8+ cells | 25.7 ± 14.6 | 22.5 ± 8.6 | 32.1 ± 22.2 | 0.3501 | 0.5119 |
| CD27+CD45RA+ % of CD8+ cells | 48.5 ± 17.9 | 51.1 ± 19.8 | 43.8 ± 14.4 | 0.4372 | 0.546 |
| CD27+CD45RA- % of CD8+ cells | 21.2 ± 8.5 | 23.8 ± 8.8 | 16.5 ± 5.9 | 0.0916 | 0.1314 |
| CD27-CD45RA+ % of CD8+ cells | 17.0 ± 12.7 | 12.0 ± 10.2 | 26.2 ± 12.4 | 0.0221 | 0.0269 |
| CD27-CD45RA- % of CD8+ cells | 13.2 ± 7.7 | 13.0 ± 8.3 | 13.5 ± 7.0 | 0.9147 | 1 |
