# Supplementary material for: Exploring the Feasibility of Multi-Site Flow Cytometric Processing of Gut Associated Lymphoid Tissue with Centralized Data Analysis for Multi-Site Clinical Trials
Source: PLoS One. 2015 May 26;10(5):e0126454. doi: 10.1371/journal.pone.0126454 (PMC4444258; doi:10.1371/journal.pone.0126454)
Supplement: S2 Table — (DOCX) [file pone.0126454.s005.docx]

**Supplemental Table 2** MMC comparisons stratified by HSV-1 status

| Characteristic | UCLA  Total  (N=18) | HSV-1  negative  (N=12) | HSV-1  positive  (N=6) | t-test  p-value  (Mean) | Wilcoxon  p-value  (Median) |
| --- | --- | --- | --- | --- | --- |
|  | Mean ± SD | | |  |  |
| Activation Panel (%) |  |  |  |  |  |
| CD4+ % of CD3+ cells | 58.1 ± 11.0 | 58.8 ± 12.6 | 56.7 ± 7.4 | 0.712 | 0.6394 |
| CD38+DR+ % of CD4+ cells | 10.1 ± 7.0 | 6.5 ± 2.5 | 17.3 ± 7.7 | 0.0179 | 0.0032 |
| CD69+ % of CD4+ cells | 52.7 ± 15.5 | 54.2 ± 16.4 | 49.5 ± 14.5 | 0.5588 | 0.6065 |
| CD8+ % of CD3+ cells | 35.0 ± 7.6 | 33.9 ± 8.4 | 37.1 ± 5.8 | 0.4156 | 0.3726 |
| CD38+DR+ % of CD8+ cells | 24.4 ± 10.0 | 21.7 ± 8.7 | 29.9 ± 11.1 | 0.1057 | 0.1223 |
| CD69+ % of CD8+ cells | 65.5 ± 16.0 | 69.2 ± 16.1 | 58.1 ± 14.2 | 0.1715 | 0.1744 |
| Memory Panel (%) |  |  |  |  |  |
| CD4+ % of CD3+ cells | 56.5 ± 11.3 | 59.1 ± 12.1 | 51.4 ± 7.9 | 0.1786 | 0.0832 |
| CCR5+ % of CD4+ cells | 71.7 ± 15.2 | 76.7 ± 8.9 | 61.7 ± 20.8 | 0.1402 | 0.1338 |
| CD27+CD45RA+% of CD4+ cells | 5.5 ± 8.3 | 1.9 ± 4.1 | 13.6 ± 10.0 | 0.0572 | 0.0065 |
| CD27+CD45RA- % of CD4+ cells | 17.8 ± 8.5 | 14.7 ± 6.8 | 24.6 ± 8.6 | 0.0269 | 0.0539 |
| CD27-CD45RA+ % of CD4+ cells | 1.8 ± 2.1 | 0.9 ± 1.1 | 3.7 ± 2.7 | 0.0862 | 0.0174 |
| CD27-CD45RA- % of CD4+ cells | 74.9 ± 15.8 | 82.5 ± 7.8 | 58.2 ± 16.5 | 0.0011 | 0.0127 |
| CD8+ % of CD3+ cells | 35.5 ± 8.3 | 33.8 ± 7.9 | 39.1 ± 8.7 | 0.2079 | 0.2415 |
| CCR5+ % of CD8+ cells | 81.3 ± 12.5 | 83.9 ± 12.2 | 76.1 ± 12.4 | 0.22 | 0.2061 |
| CD27+CD45RA+ % of CD8+ cells | 4.0 ± 4.8 | 1.7 ± 2.3 | 9.0 ± 5.4 | 0.035 | 0.0091 |
| CD27+CD45RA- % of CD8+ cells | 15.7 ± 9.1 | 14.3 ± 7.6 | 18.9 ± 12.3 | 0.3687 | 0.6915 |
| CD27-CD45RA % of CD8+ cells | 4.6 ± 9.1 | 1.2 ± 1.1 | 12.1 ± 14.3 | 0.1643 | 0.0699 |
| CD27-CD45RA- % of CD8+ cells | 75.7 ± 16.1 | 82.8 ± 9.0 | 60.0 ± 17.7 | 0.0036 | 0.0127 |
